# Unveiling the Collision-Induced Dissociation Pathways of Protonated PO3G Dimer: An Integrated Energy-Resolved MS and Quantum Chemical Study

**DOI:** 10.1021/acs.jpca.6c04525

**Published:** 2026-10-09

**Authors:** Vinicius Kuchenbecker, Nelson H. Morgon

**Affiliations:** † Institute of Chemistry, Universidade Estadual de Campinas (UNICAMP), Campinas, São Paulo 13083-970, Brazil; ‡ Chemyunion LTDA, Sorocaba, São Paulo 18087-101, Brazil

## Abstract

Poly­(trimethylene ether) glycol (PO3G) is a relevant biobased polyether whose combination of terminal hydroxyl groups and ether linkages enables multiple competing gas-phase dissociation pathways, yet its energy-dependent collision-induced dissociation (CID) remains poorly understood at the mechanistic level. Here, the protonated PO3G dimer (*m*/*z* 135) was investigated by energy-resolved mass spectrometry (ERMS) and high-level quantum chemical calculations, focusing on the main first-order fragments at *m*/*z* 117, 77, and 59. The lowest energy pathways identified presented barriers of 164.7, 176.7, and 187.3 kJ/mol, respectively, a progression qualitatively consistent with the experimental fragmentation trends. The calculations further suggest that kinetically accessible structures may differ from the thermodynamically most stable products. In particular, a protonated epoxide intermediate provides a lower-energy route to *m*/*z* 59 than the formation of protonated allyl alcohol previously suggested by automated spectral prediction approaches. A semiquantitative energy transfer model was used to assess the compatibility of the calculated barriers with the experimentally accessible collision-energy range. Within the limitations of this qualitative treatment, the combined results constrain plausible first-order dissociation pathways and provide a mechanistic framework for future quantitative studies based on precursor internal-energy distributions and RRKM kinetics.

## Graphical abstract

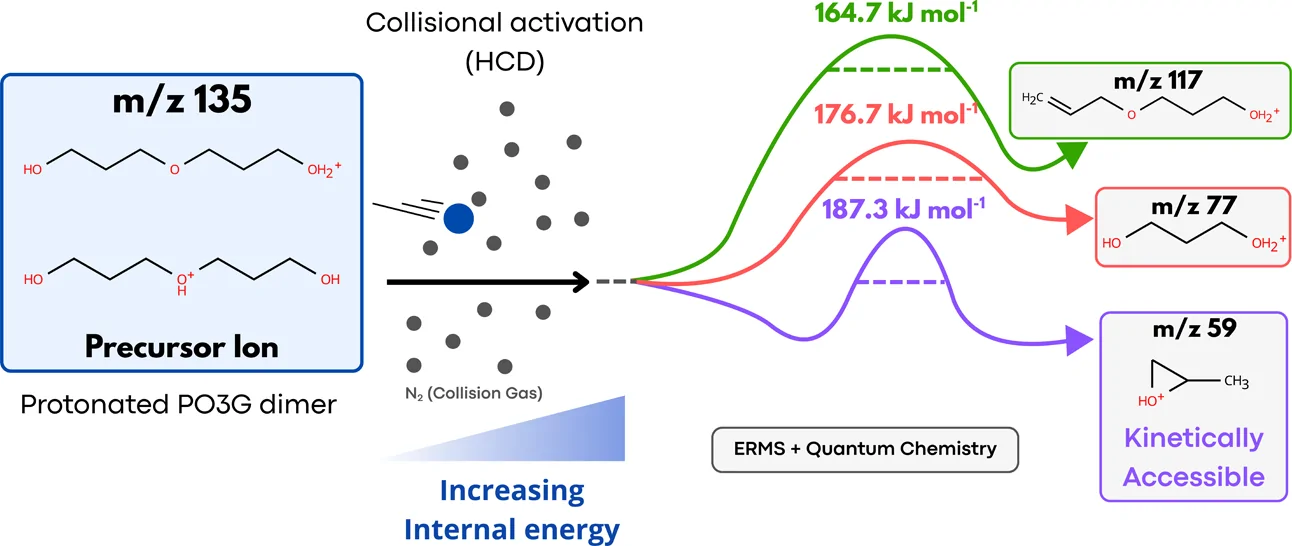

## Introduction

Arguably, the most important aspect of tandem mass spectrometry (*MS* ^2^) experiments is the dissociation reaction that converts the precursor ion into one or more fragment ions.

One of the main strengths of the *MS* ^2^ technique is its ability to add an additional layer of information to chemical analysis through the fragmentation of analyte precursor ions.^1–3^ This process enables a wide range of software and computational approaches, especially those employing artificial intelligence, to construct large compound libraries and search databases, thereby facilitating the detection and even quantification of substances in complex samples. A further layer of insight is provided by energetic information, particularly the collision energy required to induce precursor fragmentation, which greatly enhances the analytical capabilities of *MS* ^2^ experiments.^1,4,5^

Among the various methods available for precursor ion fragmentation, collision-induced dissociation (CID) is one of the most widely applied.^1,6,7^

Despite its prevalence in chemical analysis laboratories, many factors governing the formation and relative abundance of fragment ions remain uncertain. In other words, there is still a significant gap in our understanding of the reaction mechanisms underlying energy-dependent dissociation processes. A unified theoretical framework relating instrumental parameters, collision physics, energy redistribution, and reaction-coordinate evolution is still lacking. This is due, in part, to the considerable diversity in instrument design, as well as to the high cost and limited feasibility of modifying equipment to directly investigate processes occurring inside the collision cell.^1,4,6,8^

One of the available approaches for investigating these reactions is energy-resolved mass spectrometry (ERMS), which evaluates how ion intensities vary as a function of collision energy and generates breakdown curves describing the survival yield (SY) of the precursor and fragment ions.^4,6,9–11^

This technique provides an initial understanding of how different collision-energy regimes influence ion survival and fragmentation pathways. However, because the detailed conversion of kinetic energy into internal energy within the collision chamber is not yet fully understood, direct measurement of fragmentation barriers and molecular energy parameters remains inaccessible.^4,12^ Therefore, theoretical modeling and appropriate computational methods for energy and mechanism prediction are required.^1,4,6,13,14^

Since its emergence, quantum chemistry has become an essential theoretical tool for modeling reaction mechanisms and calculating energy barriers, particularly under gas-phase conditions.^15^

Complementing quantum chemistry, transition-state theory and Rice–Ramsperger–Kassel–Marcus (RRKM) theory have become standard frameworks for modeling CID processes.^16^ The latter, in particular, is closely associated with *MS* ^2^ because of its success in predicting the microcanonical rate constant *k*(*E*) for reaction channels through consideration of the sum and density of vibrational states.^1,4,6,16^

Despite significant advances in computational modeling, the prediction of theoretical *MS* ^2^ spectra remains a major challenge. A noteworthy example of a quantum-chemistry-based simulation tool is the QCxMS project by Grimme and Gorges,^13,17^ which provides two CID prediction methodologies: the original approach employing Born–Oppenheimer molecular dynamics (Version 1) and the more recent statistical implementation (Version 2), which employs conventional transition-state theory in a substantially less computationally expensive approach.

Another rapidly expanding field in *MS* ^2^ prediction is artificial intelligence, including machine learning and artificial neural networks.^18,19^ These approaches are typically developed with analytical applications in mind, prioritizing spectral prediction and compound annotation rather than mechanistic interpretation of the fragmentation process itself. Nonetheless, advances in quantum chemistry and data-driven modeling are increasingly converging toward complementary objectives.

A notable example within this category is CFM-ID,^20,21^ which is publicly accessible through a web browser and provides predicted *MS* ^2^ fragmentation spectra across multiple energy regimes for qTOF instruments, with extensions to Orbitrap systems. Although highly user-friendly, the performance of such machine-learning tools is inherently dependent on the data used for training. In many cases, no explicit mechanistic rationale is provided for a predicted fragment or for its relationship to collision energy. One approach to validating these predictions is to combine quantum chemical calculations with experimental data and assess their mechanistic and energetic consistency.

To advance the fundamental understanding of CID and improve predictive artificial intelligence tools, the publication and consolidation of ERMS data sets and mechanistic studies across a wide range of molecules are essential, particularly for revealing hidden patterns and generalized fragmentation rules.

Although *MS* ^2^ libraries are steadily expanding, comprehensive ERMS data sets are still lacking even for relatively small molecules. A representative case is poly­(trimethylene ether) glycol dimer (PO3G-d), along with other oligomeric species.

PO3G comprises an important family of polymeric materials based on 1,3-propanediol, a biobased monomer derived from corn and sugar feedstocks.^22,23^ Its synthesis is highly consistent with green-chemistry principles because it is a solvent-free, one-pot process. Recently, new protocols enabling an even greener synthesis using microwave-assisted methods have been reported.^24^

PO3G-d is the smallest oligomer that simultaneously retains the terminal hydroxyl groups and the ether linkage characteristic of the polymer. Consequently, it provides a computationally tractable model that can exhibit several fundamental fragmentation motifs of oxygen-rich polymers, including dehydration, heterolytic ether bond cleavage, proton migration, and rearrangement. Its small size makes extensive high level reaction pathway analysis feasible, while its competing dissociation channels make it sufficiently complex to serve as a mechanistic benchmark for polyether CID, future RRKM calculations, and data-driven *MS* ^2^ prediction.

In this work, we report previously unavailable ERMS data for the main characteristic fragment ions of PO3G-d, the simplest oligomer of PO3G. Alongside the experimental data, we propose reaction mechanisms for the formation of these fragment ions, considering both thermodynamic and potential kinetic products

In addition to elucidating these mechanisms, and with the aim of establishing a foundation for future quantitative studies, we use a semiquantitative model to assess the feasibility of the proposed reaction pathways. Specifically, we evaluate whether any of the calculated energy barriers are inconsistent with the ERMS data. The mechanisms are then discussed in the context of the overall survival-yield (SY) curves and idealized energy-conversion regimes.

## Materials and Methods

### Experimental Tandem Mass Spectrometry

Tandem mass spectrometry (MS^2^) data were acquired using a Thermo Fisher Scientific Exploris 120 mass spectrometer equipped with an Orbitrap mass analyzer and an electrospray ionization (ESI) source.

All samples were prepared at a concentration of 1 ppm in LC-MS grade methanol containing 0.1% (w/w) formic acid and introduced via direct infusion. The ESI source was operated in positive ion mode with a spray voltage of 3900 V. The sheath, auxiliary, and sweep gas flow rates were set to 20, 5, and 1 arbitrary units, respectively. The sample injection flow rate was maintained at 50 μL/min. The Orbitrap mass resolution was set to 30,000.

The higher-energy collisional dissociation (HCD) collision energy was manually varied using the absolute energy scale in volts, and a corresponding spectrum was recorded at each collision energy. Breakdown curves were constructed by converting the raw data files and processing them using a custom Python script for data extraction, plotting, and curve fitting.

For acquisition of the *MS* ^2^ data, the precursor ion was isolated in the quadrupole using an isolation window of ± 1 *m*/*z* prior to transfer to the C-trap and ion routing multipole (IRM), i.e., the collision cell. This procedure ensured the selective isolation of the precursor ion and minimized contributions from fragment ions generated by in-source fragmentation.

Throughout the experiment, the Automatic Gain Control (AGC) target was maintained at 100% (1 × 10^5^ charges), with a maximum injection time of 50 ms and one microscan. Data were acquired for 1 min by flow injection analysis (FIA) without a chromatographic column.

A full-scan spectrum was also recorded to assess the presence of fragmentation occurring prior to the IRM.

For the pseudo-*MS* ^3^ experiments, the same instrumental parameters were employed, except that the target mass-to-charge ratio for precursor isolation was changed to that of the ion of interest, i.e., one of the fragment ions also detected in the full-scan spectrum. After changing the precursor-ion target, the same quadrupole isolation and fragmentation procedure described above was applied.

The collision energy in the laboratory frame, *E*_lab_ (or *CE*_lab_), was calculated according to eq 1, where *z* is the charge state of the precursor ion, as measured by the instrument, and *V* is the applied potential difference in volts.

1 $$E_{\mathrm{lab}}=z\times V$$

The fractional yield (%yield), *Y*_*i*_, of a given ion *i* was calculated according to eq 2. A particular case is the fractional yield of the precursor ion, which is referred to as the survival yield (SY).

2 $$\%Y_{i}=\frac{I_{i}\left(E_{\mathrm{lab}}\right)}{\underset{j}{\sum }I_{j}\left(E_{\mathrm{lab}}\right)}\times 100$$

where *I*_*i*_(*E*_lab_) is the intensity of ion *i* at a given laboratory-frame collision energy, *E*_lab_, and ∑_*j*_ *I*_*j*_(*E*_lab_) represents the summed intensities of all ions considered at the same collision energy.

The value of *E*_lab_ at which *Y*_*i*_(*E*_lab_) = 0.5 is conventionally referred to as *CE*_50_.^11^ This definition is directly applicable when the precursor ion initially exhibits *Y*_*p*_ = 1. If the precursor ion already exhibits *Y*_*p*_ < 1 at the lowest experimental collision energy, the corresponding parameter is herein denoted as *E*_1/2_ and is defined as the energy at which

$$Y_{p}\left(E_{1/2}\right)=0.5\times Y_{p}\left(E_{\mathrm{lab},0}\right)$$

where *E*_lab,0_ is the lowest laboratory-frame collision energy employed experimentally, which was 1 eV in the present work.

To determine *E*_1/2_, a four-parameter logistic (4PL) function was fitted to the experimental SY data over the range from the highest to the lowest observed *Y*_*p*_ values, and the energy corresponding to the midpoint of this response range was calculated.

This approach follows the same general principle used to determine *CE*_50_ but is conceptually more appropriate when the precursor-ion survival yield is already below unity at the lowest experimentally accessible collision energy. Although an analogous *E*_1/2_ value may be determined for other ions, in the present work it was calculated exclusively for the precursor ion on the basis of the survival-yield curve.

### Theoretical Chemistry

#### Generation of Fragment Ion Structures

To propose thermodynamically stable product structures, the ForMileS^25^ program was utilized to generate potential constitutional isomers based on the experimental data. The generated structural ensemble was initially optimized using the Merck Molecular Force Field (MMFF). Subsequently, these structures were subjected to geometry optimization and frequency calculations using the ORCA 6.0 software package at the B3LYP-D4/def2-TZVP level of theory.

The isomers were ranked according to their relative electronic energies, and the lowest-energy structure was selected as the proposed thermodynamic product. For the ions at *m*/*z* 77 and 59, full structural generation was performed without defining a Base Molecular Scaffold (BMS).

For the ion at *m*/*z* 117, a single BMS constraint was applied to prevent the generation of chemically unrealistic structures resulting from excessive rearrangements. Following the protocol outlined in the original ForMileS publication, the BMS was selected based on the monomer precursor structure and defined as a moiety expected to remain intact during the fragmentation reaction, in this case involving water loss (−H_2_O), as suggested by the experimental data.

Potential kinetic products were proposed based on the mass spectrometry^1^ and organic chemistry^26^ literature for molecules sharing similar structural features. In the case of the *m*/*z* 59 ion, the CFM-ID prediction was used as the initial structural guess for the kinetic product.

#### Quantum Chemical Calculations and Reaction Mechanisms

Conformational sampling of the precursor ion was performed using the GOAT algorithm combined with the GFN2-xTB^27^ semiempirical method for both anticipated protomers (protonated at the ether oxygen and at the terminal hydroxyl group). A semiempirical method was chosen because of the size of the system and the nature of the constituent atoms (C, O, and H).

Transition states (TS) were located using the nudged elastic band method tailored for TS searches (NEB-TS)^28^ implemented in ORCA. All transition states were verified by the presence of exactly one imaginary frequency, and intrinsic reaction coordinate (IRC) calculations were performed to confirm that the TS connected the intended reactant and product minima. All ground-state minima were confirmed to have zero imaginary frequencies.

Final electronic single point energies (SPE) were obtained using DLPNO–CCSD­(T) with two-point complete basis set (CBS) extrapolation from the def2-TZVPP and def2-QZVPP basis sets [CBS­(3,4)], as implemented through the ORCA Extrapolate­(3/4,def2) procedure. The Self-Consistent Field (SCF) and correlation contributions were extrapolated separately according to the standard ORCA CBS extrapolation scheme.

Zero-point energy (ZPE) corrections were computed from harmonic vibrational frequencies evaluated at the same level of the geometry optimization. All calculations utilizing Density Functional Theory (DFT) incorporated Grimme’s D4 dispersion correction.^29^ Choice of functional and basis set for geometry optimization was based on previously published work.^30^

As recommended in the ORCA manual, the resolution of identity and chain-of-spheres approximations for the Coulomb and exchange integrals, respectively (RIJCOSX), were employed during geometry optimizations and frequency evaluations, utilizing matching Ahlrichs-type auxiliary basis sets.^31^ For the DLPNO–CCSD­(T) calculations, the def2-QZVPP/C auxiliary basis set was used.

Basis set superposition error (BSSE) corrections were not applied systematically throughout this work. Instead, a representative calculation was performed to assess the magnitude of the BSSE for the most strongly stabilized dimeric product complex. The Boys–Bernardi counterpoise (CP) method^32^ was employed according to eq 3, and the corresponding counterpoise-corrected interaction energy was obtained using eq 4. All energies required for the BSSE evaluation were calculated at the same level of theory employed for the single-point energy (SPE) calculations described above.

3 $$E_{\mathrm{BSSE}}\left(X\right)=\delta_{A}+\delta_{B}=\left[E_{A}^{A}\left(X\right)-E_{A}^{AB}\left(X\right)\right]+\left[E_{B}^{B}\left(X\right)-E_{B}^{AB}\left(X\right)\right]$$

4 $$\Delta E_{\mathrm{intc}}^{\mathrm{CP}}\left(X\right)=\Delta E_{\mathrm{intc}}\left(X\right)+E_{\mathrm{BSSE}}\left(X\right)$$

where *E*_BSSE_(*X*) is the BSSE correction evaluated at geometry *X*. In the present work, the correction was evaluated for a transition-state (TS) geometry and a product reaction-complex (RC) geometry. $E_{A}^{A}\left(X\right)$ and $E_{B}^{B}\left(X\right)$ are the energies of fragments A and B, respectively, at geometry *X*, calculated using only the basis functions centered on the corresponding fragment. In contrast, $E_{A}^{AB}\left(X\right)$ and $E_{B}^{AB}\left(X\right)$ are the energies of fragments A and B, respectively, at the same geometry, calculated in the full dimer basis set by including the basis functions of the complementary fragment as ghost functions. Thus, fragment A is calculated using its own basis functions together with the ghost basis functions of fragment B, whereas fragment B is calculated using its own basis functions together with the ghost basis functions of fragment A. Finally, $\Delta E_{\mathrm{intc}}^{\mathrm{CP}}\left(X\right)$ denotes the interaction energy corrected using the Boys–Bernardi counterpoise method, whereas Δ*E*_intc_(*X*) is the corresponding uncorrected interaction energy.

All quantum chemical calculations were performed using the ORCA 6.0 program.^33^

## Results and Discussion

### Energy-Resolved Mass Spectrometry

A first-order dissociation refers to a fragmentation process that generates ions directly from dissociation of the precursor ion. Second-order dissociation arises from the dissociation of a fragment ion generated in a first-order process, that is, from the accumulation of internal energy within the fragment ion leading to further dissociation, with analogous processes occurring for higher-order events.^1^ This work focuses on the reaction mechanisms leading to first-order fragment ions.

Figure 1 provides an overview of the ERMS data acquisition, including the *MS* ^2^ spectrum acquired at 1 eV *CE*_lab_ and the evolution of the relative and absolute intensities of the main ions as a function of *CE*_lab_. The full set of spectra is available in the Supporting Information (Figure S3–S22).

**Figure 1:**
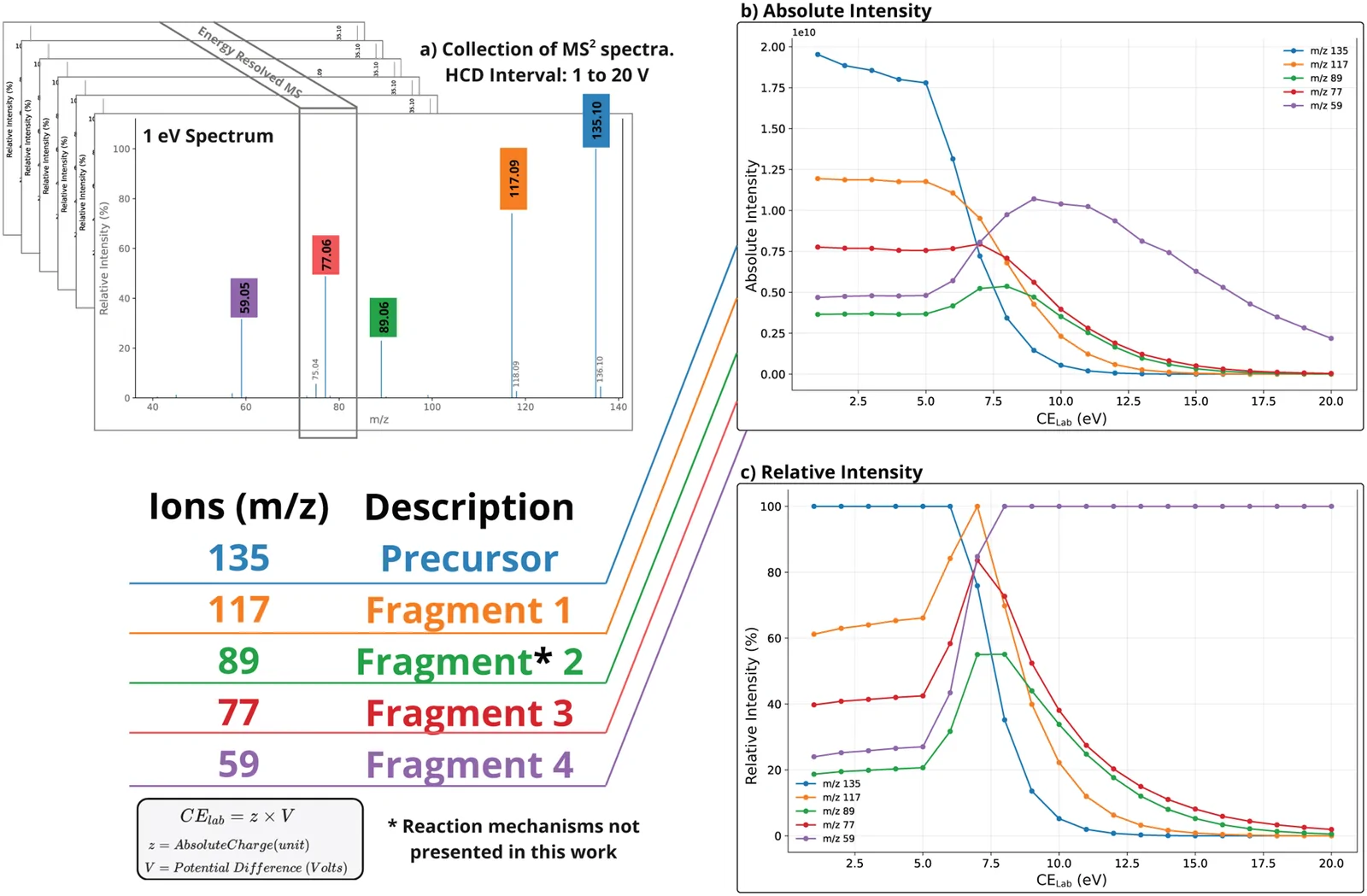
(a) Illustration of spectral acquisition for ERMS analysis. The *MS* ^2^ spectrum acquired at 1 eV collision energy is shown together with the list of main ions. Breakdown curves are shown for (b) absolute and (c) relative intensities.

The principal ions selected as the fragment ions characterizing the dissociation pattern of the PO3G-d precursor ion are *m*/*z* 117, 77 and 59.

Among the structures generated by ForMileS for *m*/*z* 89, the plausible products involved the loss of CO_2_ and H_2_O. Attempts to model a reaction mechanism yielding a fragment consistent with this neutral loss invariably led to the formation of *m*/*z* 117 as an intermediate. Supporting this interpretation, a pseudo-*MS* ^3^ experiment showed that *m*/*z* 89 is also formed by fragmentation of *m*/*z* 117 (Figure S26 in SI). In the ERMS experiment using *m*/*z* 135 as the precursor, the fractional yield of *m*/*z* 89 was lower than that of the other monitored fragment ions. Taken together, these observations suggest that future studies should examine the experimental and theoretical aspects of *m*/*z* 89 formation in greater detail, together with second and higher order dissociation processes.

Figure 2 presents the fractional yield (%yield) information on the main ions.

**Figure 2:**
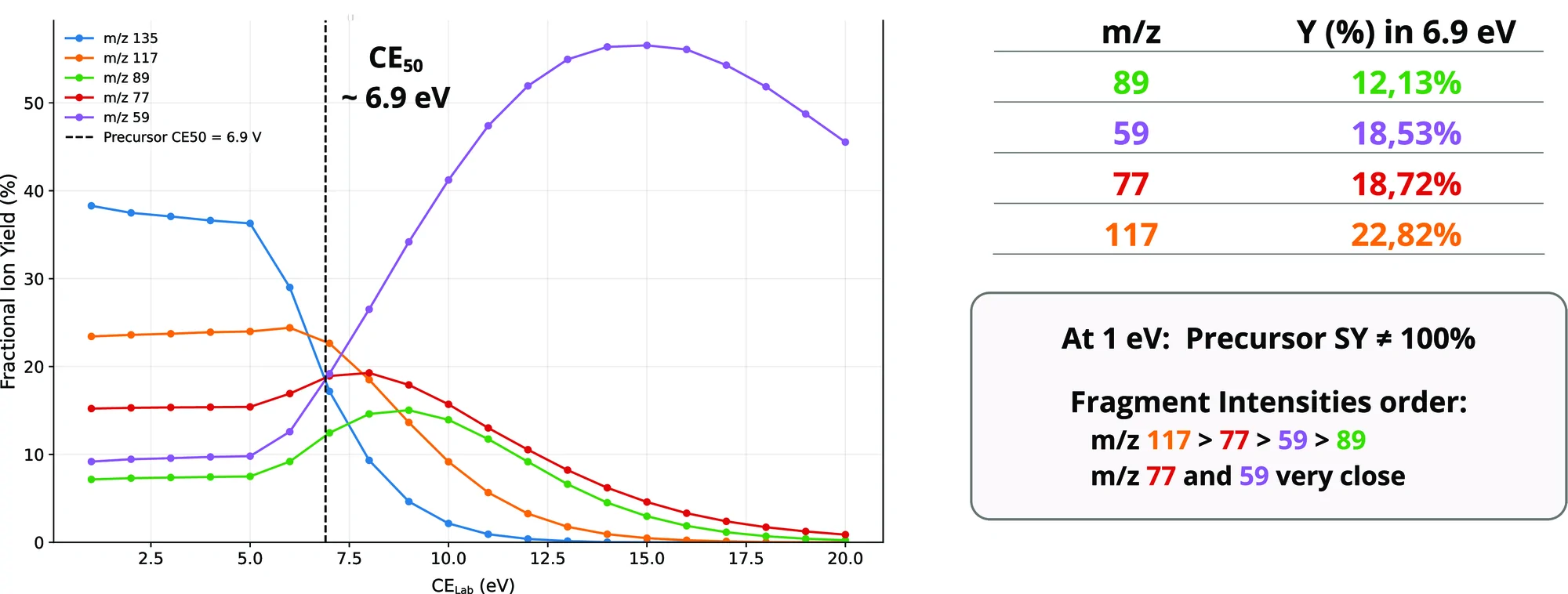
Fractional yield (%yield) plot for the main ions. An ordered list of fragment-ion %yield values in the region of the precursor-ion *E*_1/2_ (or *CE*_50_) is shown.

Table S9 in the Supporting Information reports the injection time for each fragment ion throughout the ERMS experiment.

As shown in the full-scan spectrum in Figure S23 of the Supporting Information, as well as in the %yield plot, in-source fragmentation occurs. Despite quadrupole isolation of the precursor, at the lowest available collision energy, i.e., 1 eV, both precursor and fragment ions are present. Consequently, the precursor SY does not begin at 1 (or 100%). Similar behavior has been reported in other studies^11,34–36^ and indicates that the precursor is highly labile toward fragmentation. Lower collision energies could have provided additional data but were not experimentally accessible because of instrumental limitations.

There is also a region up to approximately 5 eV in which the changes in %yield are subtle, visually resembling a plateau. Within this nearly linear region, the increase in *m*/*z* 117 is slightly more pronounced than those of the other monitored fragment ions.

The *E*_1/2_ for the precursor is the equivalent of a *CE*_50_ in the physical sense, considering the starting population of the precursor ion.

Notably, in the precursor-dissociation region, the %yield values follow the order *m*/*z* 117, 77, 59, and 89, with the values for *m*/*z* 77 and 59 being very close.

Because the AGC target was fixed and the maximum injection time was sufficient to reach the target ion population, the observed %yield order may provide a rough indication of the relative formation of the ions. However, several uncontrolled parameters may also affect this ordering. Therefore, this interpretation is valid only if all other experimental conditions are comparable across injections and analyses.

Beyond the *E*_1/2_ region of the *m*/*z* 135 ion, the overall %yield ordering changes, most likely because of secondary fragmentation. Indeed, the %yield data, together with the pseudo-*MS* ^3^ spectra presented in the Supporting Information, show that secondary reactions, i.e., second and higher order dissociation processes, contribute to the overall CID behavior of PO3G-d.

The SY also indicates that higher collision energies could have been used to characterize the complete dissociation profile of the *m*/*z* 59 ion. However, because this was not the primary focus of the present work, the experimental range was limited to a maximum of 20 eV, beyond the point at which the precursor ion SY reaches zero.

Further details and discussion pertaining to the experimental data are provided in conjunction with the reaction mechanism analysis.

### Fragment Ion Molecular Structures Using ForMileS

The ForMileS program was applied to help systematize structural hypotheses for the three fragment ions with respect to the available experimental data.

For *m*/*z* 117, the BMS used was the SMILES string “occco”, representing the core part of the ion expected to remain unchanged. Both the branched (″with ramification″) and unbranched (″without ramification″) options were evaluated, and their relative energies were calculated to identify the lowest-energy structure as the thermodynamic product candidate.

Upon inspection, the low-relative-energy branched structures generated by the program involved excessive rearrangements that were chemically unexpected for the *m*/*z* 117 ion. Conversely, when the ramification option was disabled, the resulting set of structures was far more chemically plausible; from this set, the thermodynamic structure was determined and labeled as 117B, which corresponds to a protonated secondary alkene ether structure. Because previous studies^37–39^ and literature^1,26^ suggest that the kinetic structure for polyether glycol ions generated via H_2_O loss could be a primary alkene with a protonated alcohol or ether, this pathway was labeled as 117A.

For the ion at *m*/*z* 77, the lowest-relative-energy isomer is indeed the expected protonated monomer structure, implying that the kinetic and thermodynamic products share the same structure.

For the ion at *m*/*z* 59, the lowest-energy isomer generated was a protonated ketone oxonium ion, labeled as 59B. The kinetic product, on the other hand, is expected to be a protonated allylic alcohol (following the alkene + protonated oxygen atom pattern mentioned for *m*/*z* 117) and was labeled as 59A.

These are the core structures utilized for the reaction mechanism study, selected based on a combination of experimental data, ForMileS outputs, and literature precedents. Any additional structural insights were expected to be elucidated during the reaction coordinate modeling as discussed below.

### Reaction Mechanisms

The reaction coordinate diagrams are consistently plotted with the precursor ion defined as the initial state. Light green arrows indicate steps included in the reaction coordinate that do not correspond to explicit transition states, such as transitions between conformational states or steps following elimination of a neutral molecule from the system.

BSSE was evaluated for both TS1 and Int1 (RC) of the 135–59A pathway because this channel produces the most strongly stabilized dimeric interaction. Tables S11–S12 in the Supporting Information (SI) present the corresponding values and differences between states. Full details of the calculation are provided in Section 6 of the SI. As previously discussed,^40,41^ for small systems treated with large basis sets and a CBS extrapolation scheme, the BSSE correction may not significantly improve the final energy. This is supported by the values obtained for the two cases examined here. Therefore, extensive BSSE corrections were not applied to all geometries in this work. A comprehensive assessment of BSSE effects on the remaining geometries could be undertaken in future quantitative studies.

#### Fragment Ion *m*/*z* 117 Formation

Previous studies have reported water elimination as a common and expected neutral loss from protonated glycols and other oxygen rich species.^38,39^ After separation of the van der Waals complex, the final product equilibrates to a structure protonated at the ether oxygen.

Regarding the conformers, the searches identified p1c1 as the reactive structure, whereas p2c1 corresponds to the lowest-energy precursor conformer. Both are included in the reaction coordinate. Searches initiated from other conformers converged to the same structure, and this procedure was adopted for all other mechanisms.

An alternative two-step pathway involving the formation and opening of a protonated epoxide ring was also identified and is shown in Figure S1 of the Supporting Information. Nevertheless, the calculated barriers support the concerted water-elimination pathway Figure 3.

**Figure 3:**
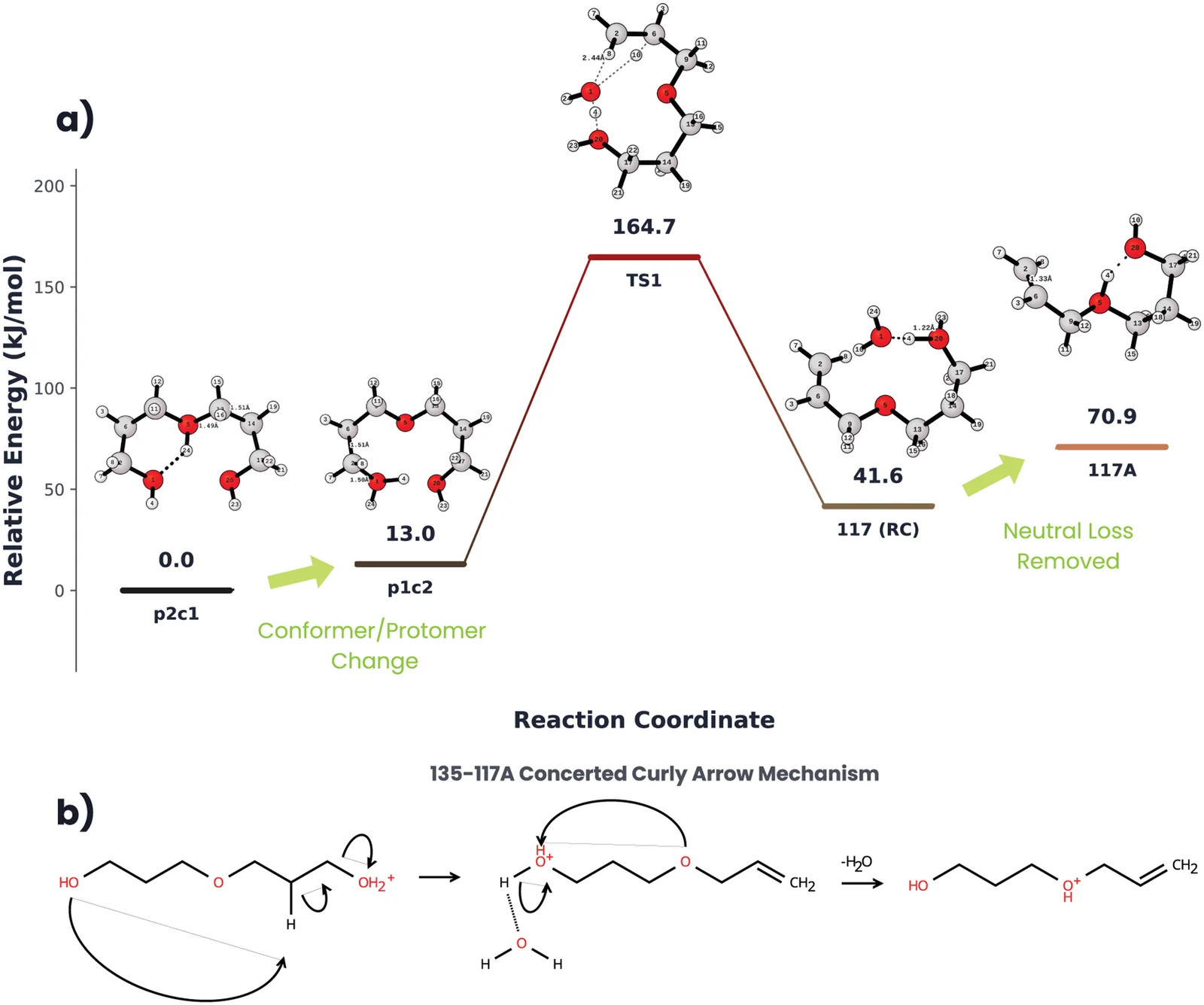
Proposed dissociation mechanism connecting the precursor ion at *m*/*z* 135 with product ion 117A via concerted pathway. Panel (a) shows the computed reaction coordinate profile and panel (b) shows the corresponding curly arrow mechanism.

After dissociation, the neutral species is considered separated from the fragment ion in subsequent steps of the reaction coordinate.^1^ Because the system is microcanonical, the energy available for dissociation corresponds to the internal energy of the precursor ion. Therefore, all energies are referenced to the lowest-energy conformer/protomer.

For the stepwise mechanism leading to 117A, although only 82 kJ/mol is required for ring opening of the epoxide intermediate, the precursor ion would require an increase of approximately 208 kJ/mol in internal energy to overcome this barrier and form the 117A structure.

Another possibility involves the formation of the thermodynamically favored product. The identified reaction pathway is shown in Figure 4. Because the only identified reaction coordinate proceeds through the initial formation of 117A, the proposed pathway involves concerted formation of 117A followed by isomerization, whereas the stepwise pathway is shown in Figure S2 in SI.

**Figure 4:**
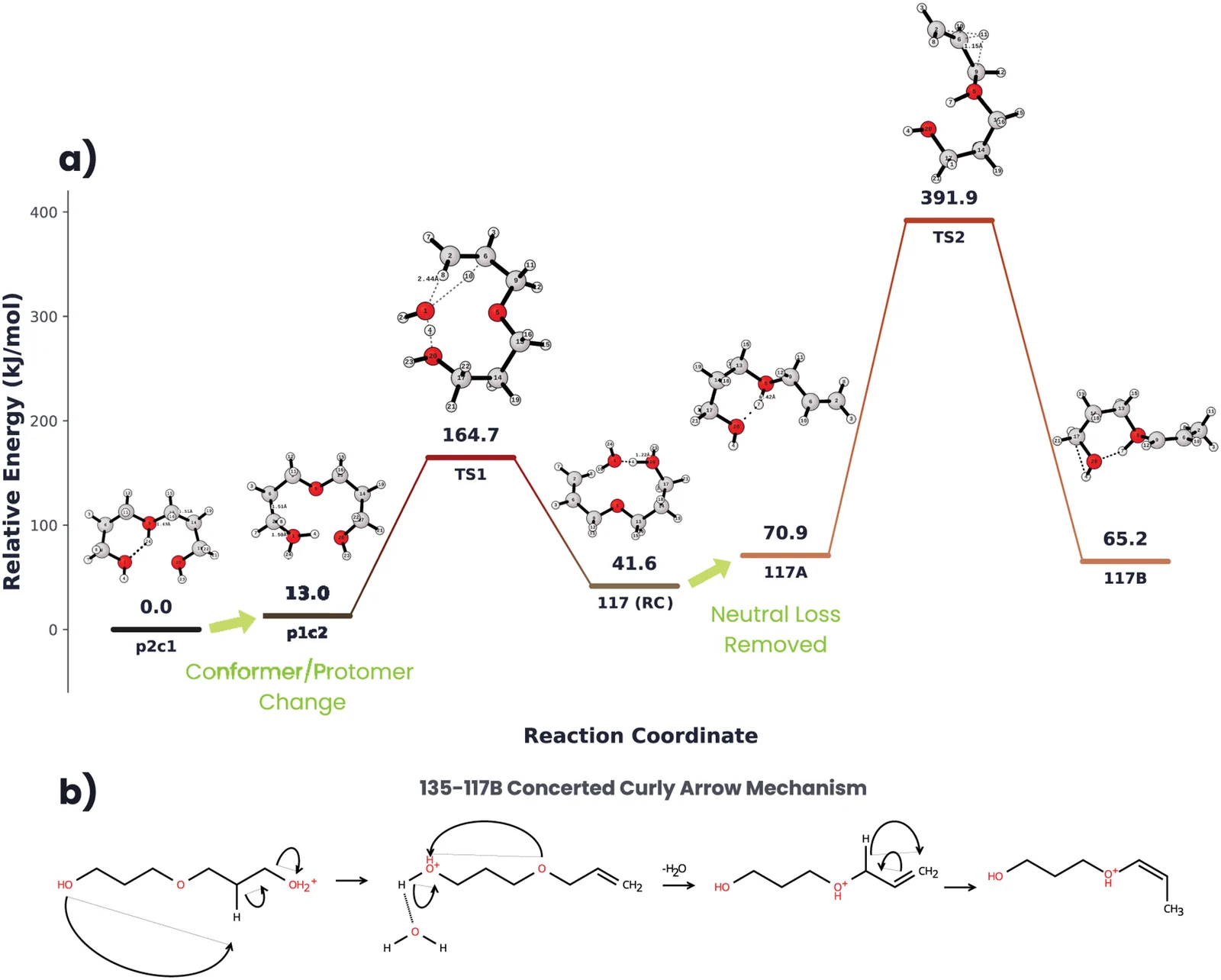
Proposed dissociation mechanism connecting the precursor ion at *m*/*z* 135 with product ion 117B via concerted/isomerization pathway. Panel (a) shows the computed reaction coordinate profile and panel (b) shows the corresponding curly arrow mechanism.

Regardless, the reaction barrier to form 117B is found to be significantly higher than that for 117A.

#### Fragment Ion 77 Formation

The only mechanistic pathway found for this fragment is presented in Figure 5.

**Figure 5:**
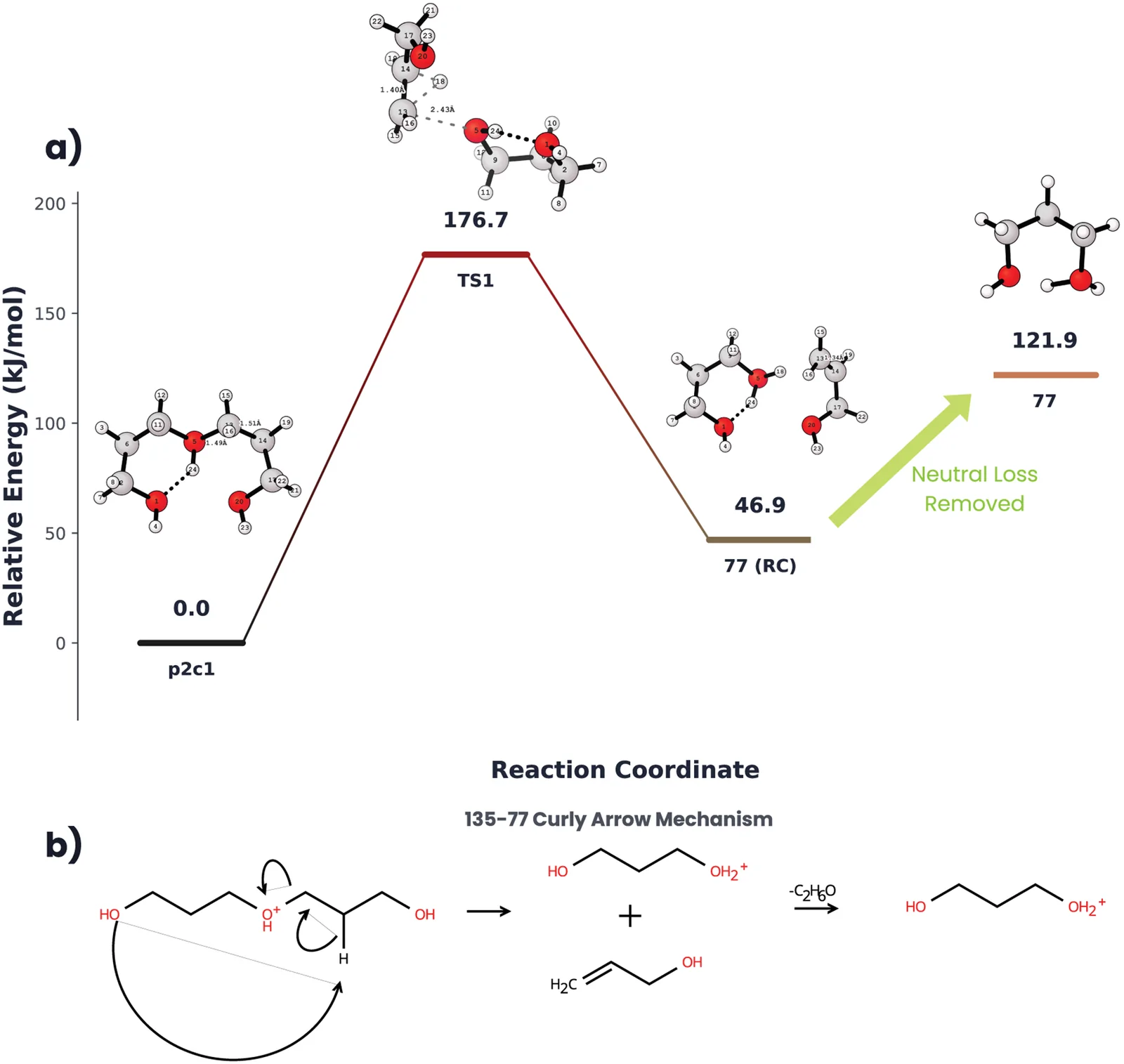
Proposed dissociation mechanism connecting the precursor ion at *m*/*z* 135 with product ion 77. Panel (a) shows the computed reaction coordinate profile and panel (b) shows the corresponding curly arrow mechanism.

This mechanism presents an interesting case in which direct cleavage yields the PO3G monomer as a protonated 1,3-propanediol species. Previous studies^38,42,43^ have similarly reported that oligomeric and polymeric protomers can fragment to produce a protonated monomer, which aligns with our observations here.

The mechanism proceeds via heterolytic cleavage of the ether bond. The resulting energy barrier is slightly higher than that for formation of 117A from *m*/*z* 135, indicating that the two pathways have very similar energetic requirements.

#### Fragment Ion 59 Formation

The mechanism in Figure 6, as well as that shown in Figure 7, exhibits an important feature: the high energy required to separate the neutral species from the ion–neutral complex following bond cleavage.

**Figure 6:**
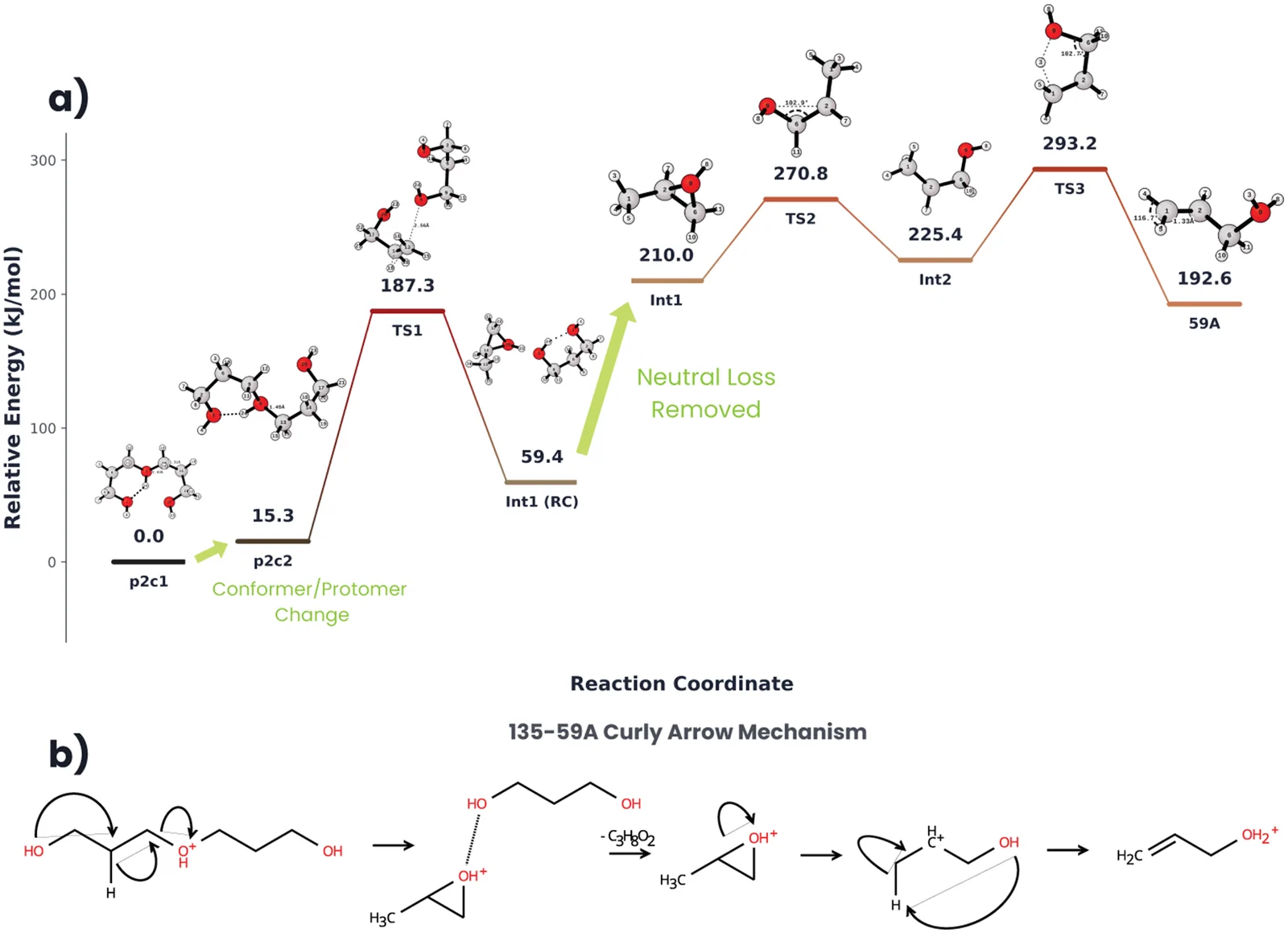
Proposed dissociation mechanism connecting the precursor ion at *m*/*z* 135 with product ion 59A. Panel (a) shows the computed reaction coordinate profile and panel (b) shows the corresponding curly arrow mechanism.

**Figure 7:**
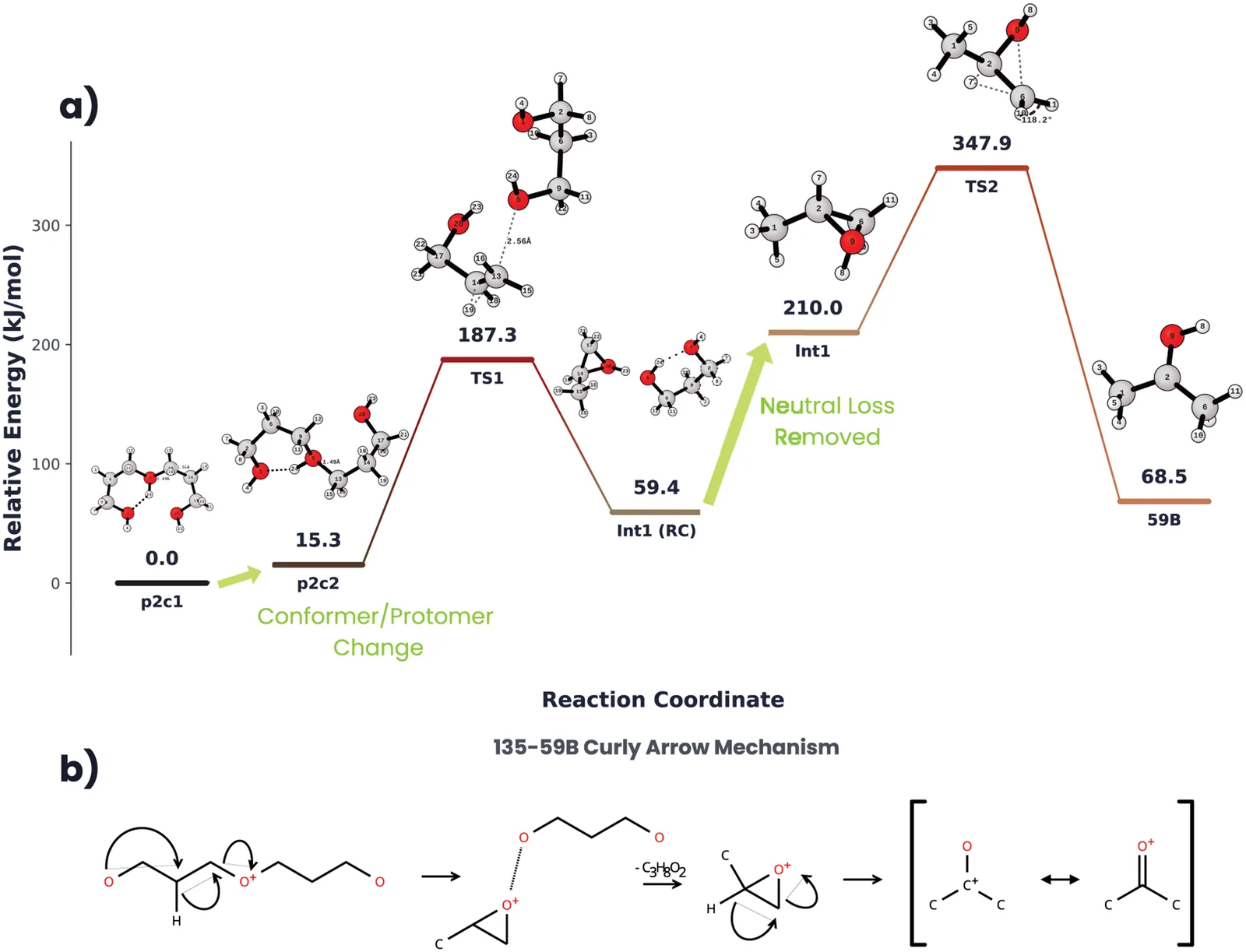
Proposed dissociation mechanism connecting the precursor ion at *m*/*z* 135 with product ion 59B. Panel (a) shows the computed reaction coordinate profile and panel (b) shows the corresponding curly arrow mechanism.

This behavior is mainly associated with the need to constrain the proton on the *m*/*z* 59 ion in order to study the mechanism. Although the vibrational frequencies confirm the presence of a local minimum, the system readily rearranges toward a more stable minimum: the formation of the *m*/*z* 77 ion. In other words, upon dissociation of the complex, the formation of the *m*/*z* 77 ion is energetically more favorable than that of the *m*/*z* 59 ion.

This is reasonable because (a) formation of a constrained epoxide ring is less favorable than formation of the unconstrained protonated alcohol corresponding to *m*/*z* 77, and (b) the additional nonprotonated oxygen atom in the *m*/*z* 77 ion provides greater electron density and therefore better stabilization of the charged site.

Therefore, while the first barrier is not particularly high and remains within the same order of magnitude as the other barriers discussed so far, dissociation of the van der Waals complex provides a clearer view of the thermodynamic preference for the *m*/*z* 77 ion over the initially formed *m*/*z* 59 structure, referred to as intermediate 1. Consequently, the highest energetic cost along this reaction coordinate corresponds to separation of the neutral fragment, leaving only the *m*/*z* 59 Int1 species in the gas phase.

After this step, the barriers relative to the *m*/*z* 59 ion, int1, are no higher than 61 and 88 kJ/mol. However, when referenced to the initial energy of the precursor ion, these barriers reach approximately 270 and 298 kJ/mol.

There is evidence in the literature that *m*/*z* 59A may correspond to protonated allyl alcohol. This is also the structure proposed by CFM-ID. In the CFM-ID prediction, protonated allyl alcohol at *m*/*z* 59 is expected to form at approximately 10 eV, although the model was originally developed for qTOF instruments and later extended to Orbitrap systems.

Therefore, the first kinetically accessible *m*/*z* 59 structure along the identified pathway is a protonated epoxide intermediate (Int1). Because this is an intermediate, whether it reaches the detector depends on its lifetime and on the residence time of the fragment ion in the IRM before detection. Here, only its role within the reaction mechanism can be evaluated.

### Experimental Validity of the Theoretical Mechanisms

Based on the fractional yield data, all fragment ions increase in intensity near the precursor *E*_1/2_, while the precursor intensity decreases.

In the pseudo-*MS* ^3^ experiments (Figures S24–S26), *m*/*z* 117 generates *m*/*z* 89 and 59, but not *m*/*z* 77. As expected, *m*/*z* 117 can only be generated through precursor dissociation. The *m*/*z* 77 ion does not appear to be generated from *m*/*z* 89. *m*/*z* 89, in turn, is formed from both *m*/*z* 135 and 117, whereas *m*/*z* 59 is generated from *m*/*z* 135, 117, 89, and 77. Although this work focuses on first order dissociation products, these experimental findings help interpret the fractional-yield breakdown curve shown in Figure 2.

The decrease in %yield from *m*/*z* 117 to *m*/*z* 59 could suggest a lower barrier for formation of the former than of the latter because, assuming all other experimental parameters are equal, the %yield depends in part on the number of ions generated.^1^ In this respect, the calculated barrier trend is consistent with the experimental observation, even when the lowest barrier for *m*/*z* 59 formation from *m*/*z* 135 is considered.

To check if the calculated barriers are compatible with the experimental conditions in terms of order of magnitude, the energy transfer model of Kuki et al.,^4,37^ previously applied to peptides and polyethers, was used. The model provides an upper limit for the conversion of collision kinetic energy into internal energy, according to eq 5

5 $$E_{\mathrm{int},\mathrm{inc}}=\overset{Z}{\underset{i=1}{\sum }}\Delta E_{\mathrm{int},i}=\frac{\eta E_{\mathrm{kin},0}}{\gamma }\left[1-{\left(1-\beta \gamma \right)}^{Z}\right]$$

where η is the overall collision inelasticity, while γ and β are center-of-mass terms related to collisions between bodies of different masses, defined, respectively, as (2*m*_*p*_ + η*m*_*g*_)/(*m*_*p*_ + *m*_*g*_) and *m*_*g*_/(*m*_*g*_ + *m*_*p*_), and *Z* represents the number of collisions. *E*_kin,0_ and *E*_int,inc_ represent, respectively, the initial kinetic energy in the laboratory frame and the increment in internal energy after the *Z*-th collision. The parameters *m*_*g*_ and *m*_*p*_ correspond, respectively, to the masses of the collision gas and the precursor ion molecule.

The two critical variables that are nontrivial to measure or calculate^4,12,44^ are η and *Z*. The remaining variables depend exclusively on the masses, which are known for the experiment under consideration. As *Z* increases, the geometric sequence [1 – (1 – βγ)^*Z*^] approaches 1 when *m*_*p*_ ≫ *m*_*g*_, as is generally the case in MS^2^ experiments.^4^ Under perfectly inelastic conditions, γ approaches 2. Therefore, the maximum amount of internal energy that the precursor ion can acquire in a CID experiment does not exceed one-half of its kinetic energy in the laboratory frame.^4,37^

For PO3G-d, assuming a completely inelastic process, the maximum increase in internal energy is *E*_int,inc,PO3G‑d_ = *E*_cin,0_/1.85. Evaluation of progressively increasing values of *Z* showed that the geometric sequence converges to a plateau after 30 collisions. Because no theoretical or experimental study has, to date, reported collision cross-section values for PO3G-d, calculation of the average number of collisions occurring over the length and time scale of the experimental collision cell remains beyond the scope of this work.^37^

The maximum increment in internal energy is added to the initial theoretical internal energy, calculated according to eq 6 using the vibrational modes of p2c1, the lowest-energy conformer/protomer of the precursor ion, obtained from the frequency calculation. The internal energy calculation includes only the vibrational modes and excludes rotational contributions, consistent with previous studies and with the RRKM assumption that vibrational modes provide the most significant contribution to the internal energy associated with dissociation.^4,12^

6 $$E_{int,m}\left(T\right)=N_{A}\overset{3N-6}{\underset{i=1}{\sum }}\frac{hc{\mathrm{\nu ̃}}_{i}}{\exp\left(\frac{hc{\mathrm{\nu ̃}}_{i}}{k_{B}T}\right)-1}$$

*h* is Planck’s constant, *c* is the speed of light, *k*_*B*_ is the Boltzmann constant, *T* is the absolute temperature (298 K in this case), *N*_*A*_ is Avogadro’s constant, and *ν̃*_*i*_ is the frequency of the *i*-th vibrational mode. The summation is performed over the total number of vibrational modes in the molecule, which is 3*N* – 6 for a nonlinear molecule with *N* atoms, resulting in the molar vibrational internal energy *E*_int,*m*_(*T*).^16,45^

The effective temperature of the precursor ion population is unknown experimentally, but it cannot be lower than the ambient temperature at which the experiment was performed. Table S4 presents a series of calculations from 298 to 1000 K. Because the incremental-energy model is used here to define theoretical regimes for a qualitative discussion, temperature can be regarded as a parameter representing the degree of precursor excitation prior to the first collision. Increasing the assumed temperature from 298 to 400 K raises the initial internal energy from approximately 25 to 43 kJ/mol. As discussed below, this difference does not alter the qualitative conclusions. Nevertheless, this theoretical treatment highlights both the difficulty and the importance of modeling the precursor ion internal energy distribution for quantitative mechanistic evaluation, particularly when thermal excitation prior to collision is considered.

The following discussion uses these values only to assess whether some reaction products would require highly idealized collision conditions and/or substantial thermal excitation before collision to become favorable, given the lack of precise values for the aforementioned parameters.

To establish boundary conditions for evaluating the proposed mechanisms, four ″collision energy regimes″ were defined according to the aforementioned physical model, which are summarized in Table 1.

**Table 1:** Theoretical Internal Energy Conversion Regimes for the *m*/*z* 135 Ion at 298 K

*m*/*z*	Region (kJ/mol)	(**A**) η = 1; *Z* = 100	(**B**) η = 0.5; *Z* = 100	(**C**) η = 1; *Z* = 3	(**D**) η = 0.5; *Z* = 3
135	Dissociation	381.1	212.1	267.6	146.3

The regimes labeled A and B are calculated assuming that the experimental conditions allow a large number of collisions. In contrast, regimes C and D represent plausible scenarios during the early, approximately linear stage of the process, when only a few collisions are expected to have occurred.

#### Comparing Reaction Barriers Against Calculated Energy Regimes

Table 2 presents the reaction barriers determined for the aforementioned mechanisms, along with brief assessments of each.

**Table 2:** Comparison of Transition State Energies (Reaction Barriers) and Their Qualitative Evaluation

Barrier	Δ*E* ^0^(kJ/mol)	Evaluation
135–117A Concerted – TS1	164.7	1st lowest – Leads to 117A
135–117B Concerted/Isomerization – TS1	164.7	1st lowest – Leads initially to 117A
135–77 – TS1	176.7	2nd lowest – Leads to *m*/*z* 77
135–117A Stepwise – TS1	177.4	3rd lowest – Leads to Int1
135–117B Stepwise – TS1	177.4	3rd lowest – Leads to Int1
135–59A – TS1	187.3	4th lowest – Leads to Int1
135–59B – TS1	187.3	4th lowest – Leads to Int1
135–117A Stepwise – TS2	209.7	5th lowest – Leads to 117A
135–117B Stepwise – TS2	209.7	5th lowest – Leads to 117A
135–59A – TS2	270.8	6th lowest – Leads to Int2
135–59A – TS3	293.2	High-energy barrier – Leads to 59A
135–59B – TS2	347.9	High-energy barrier – Leads to 59B
135–117B Concerted/Isomerization – TS2	391.9	High-energy barrier – Leads to 117B
135–117B Stepwise – TS3	391.9	High-energy barrier – Leads to 117B

Although the inelasticity of PO3G-d remains unknown, studies on peptides larger than PO3G-d have reported inelasticity values between 50% and 60%.^4,37^ While this range should not be taken as definitively applicable to the present work, it serves as a valuable illustration.

The full-scan MS1 spectrum acquired before fragmentation (Figure S23) and the absence of clear formation curves for *m*/*z* 117 and 77 (Figure 2) suggest that PO3G-d is highly metastable.^1^ It fragments readily even when ESI parameters are adjusted to minimize in-source fragmentation. Thus, the reaction barriers leading to these ions are expected to be readily accessible.

Even the regime D with a low effective temperature for internal energy provides around 146 kJ/mol of internal energy, not very far from 164 and 176 kJ/mol required to form ion *m*/*z* 117A and 77, respectively. In theory, with slightly more collisions and/or more thermal excitation the precursor ion population could have a internal energy distribution around these values, i.e., there is feasibility in these barriers compared with the experimental data.

If, at *E*_1/2_, the precursor had sufficient energy to form *m*/*z* 59A (293.2 kJ/mol), a steeper decrease in the *m*/*z* 117%yield might be expected, at least in the region where the *m*/*z* 59%yield increases sharply (Figure 2). If the precursor internal-energy distribution does not extend to approximately 300 kJ/mol, the early formation of *m*/*z* 59 would require an alternative kinetically favorable pathway. However, because *m*/*z* 117 and 77 are present from the beginning of the experiment and both generate *m*/*z* 59 (Figures S24 and S26), these ions may also contribute to the early formation of *m*/*z* 59. Therefore, without further investigation of second and third order dissociation processes, this interpretation remains a hypothesis supported by the combined experimental and theoretical observations.

In theory, the maximum internal energy gained in a perfectly efficient collisional process (regime A, Table 1) with room-temperature thermal excitation is approximately 381 kJ/mol. If a higher effective temperature were reached, this value could increase to approximately 404 and 587 kJ/mol at 500 and 1000 K, respectively.

Under these idealized conditions, all barriers listed in Table 2 could, in principle, be surmounted. However, the values discussed above correspond to highly idealized and likely unrealistic collision energy conversion conditions. Even with η = 0.5, no more than approximately 350 kJ/mol would be available at 1000 K under the additional idealization of *Z* → ∞. This value is only slightly above the barrier for formation of *m*/*z* 59B (347.9 kJ/mol, Table 2) and remains below that required to form *m*/*z* 117B (391.9 kJ/mol).

The literature^1,6,7,12,46,47^ also indicates that HCD systems are designed such that precursor ions undergo only a few collision events. Therefore, even the *Z* → ∞ extrapolation is likely to be highly idealized.

Accordingly, the final four entries in Table 2 are labeled ″High-energy barrier″ to emphasize that these reaction pathways would require substantial thermal excitation before collision and, in the case of *m*/*z* 117B, very efficient conversion of kinetic energy into internal energy during the collisional process.

Thus, although we do not conclude that these products are not formed, they appear to be substantially less favorable. This may indicate that (a) other reaction mechanisms not identified here contribute to their formation directly from *m*/*z* 135; (b) in the case of *m*/*z* 59, the product is also formed through secondary reactions, and/or (c) more kinetically favorable structures are formed as the major products of precursor dissociation for these ions, such as 117A and one of the 59A intermediates.

Given that the pseudo-*MS* ^3^ spectrum of *m*/*z* 117 shows no peak at *m*/*z* 77, together with the discussion of relative %yield ordering and activation barriers and the chemically reasonable and simple dissociation mechanism, we consider the *m*/*z* 135–77 pathway a strong mechanistic proposal for future quantitative evaluation.

Furthermore, because of the statistical nature of the reaction process and the likely occurrence of higher order dissociation events, an ensemble of molecular structures may represent a given ion rather than a single structure. For example, the structure of the *m*/*z* 59 ion formed within a low energy regime may differ from that formed at higher energies, and competing pathways may generate mixed isomer populations.

Here, the discussion is restricted to the energy region in which the precursor dissociates and to the possible precursor-dissociation reactions themselves.

These results highlight that the analysis should extend beyond activation barriers to include rate constants, such as those calculated using RRKM theory.^1,4,44,48^ The internal-energy distribution must also be considered for a more rigorous quantitative assessment, together with mechanistic modeling of second and higher order dissociation processes.

Nonetheless, RRKM rate constant calculations require an initial set of reaction mechanisms and associated transition states. In this regard, the mechanisms proposed here provide a sound starting point for a quantitative study of PO3G-d precursor dissociation.

## Conclusion

This work combined energy resolved mass spectrometry and high level quantum chemical calculations to examine plausible first order CID pathways of protonated PO3G-d (*m*/*z* 135). Reaction mechanisms were evaluated for the formation of the main fragment ions at *m*/*z* 117, 77, and 59, with calculated barriers of 164.7, 176.7, and 187.3 kJ/mol for the lowest energy pathways identified. The ordering of these barriers is qualitatively consistent with the experimental ERMS behavior observed during precursor dissociation.

The calculations indicate that kinetically accessible structures may differ from the thermodynamically most stable products. For *m*/*z* 117, formation of 117A is substantially less demanding than subsequent conversion to 117B. For *m*/*z* 59, the protonated epoxide intermediate is reached at considerably lower energy than protonated allyl alcohol. These results therefore provide plausible mechanistic alternatives to assignments based solely on thermodynamic stability or automated spectral prediction.

The energy-transfer model was used only as a qualitative boundary analysis and does not define the actual internal energy distribution of the precursor ion population. Within this limitation, it helps distinguish pathways that are compatible with the experimentally accessible energy range from those requiring comparatively demanding conditions. The present results should therefore be regarded as mechanistically and energetically consistent proposals rather than definitive structural assignments.

Overall, the combined experimental and theoretical analysis narrows the set of plausible first order dissociation pathways for PO3G-d and establishes a basis for more quantitative treatment. Future work incorporating precursor internal-energy distributions, RRKM rate constants, and second and higher order fragmentation pathways will be required to test these assignments more rigorously and to describe the energy dependent CID behavior of the system quantitatively.

## Supplementary Material



## Notes

Quantum-chemical reaction-coordinate energies and proposed dissociation mechanisms; energy-resolved MS/MS data and individual spectra; full-scan MS1 and pseudo-MS3 spectra; BSSE calculations; Cartesian coordinates of optimized structures and transition states; and ForMileS molecular-graph output (PDF)

## Abbreviations

PO3G-d: Poly­(trimethylene ether) glycol dimer
CID: Collision-induced dissociation
*MS* ^2^: Tandem mass spectrometry
ERMS: Energy resolved mass spectrometry
SY: Survival yield
*CE*_50_: Collision energy for 50% intensity reduction of precursor ion
BMS: Base molecular Scaffold
ZPE: Zero-point energy
ESI: Electrospray interface
HCD: Higher-collisional energy dissociation
IRC: Intrinsic reaction coordinate
PES: Potential energy surface
