# Supplementary material for: Unveiling the Collision-Induced Dissociation Pathways of Protonated PO3G Dimer: An Integrated Energy-Resolved MS and Quantum Chemical Study
Source: J Phys Chem A. 2026 Oct 9;130(42):8361–73. doi: 10.1021/acs.jpca.6c04525 (PMC13700093; doi:10.1021/acs.jpca.6c04525)
Supplement: Supplementary file 1 [file jp6c04525_si_001.pdf]

Supporting Information

Unveiling the Collision-Induced Dissociation Pathways of Protonated PO3G Dimer: An Integrated Energy-Resolved MS and Quantum Chemical Study

Vinicius Kuchenbecker[1,2,*] and Nelson H. Morgon[1]

[1]Institute of Chemistry, University of Campinas (UNICAMP), Campinas, São Paulo 13083-970, Brazil
[2]Chemyunion Ltda., Sorocaba, São Paulo 18087-101, Brazil
[*]Corresponding author. E-mail: vinicius.kuchenbecker@gmail.com

Proposed dissociation mechanisms, energy-resolved MS/MS data, full-scan MS1 and pseudo-MS3 spectra, quantum-chemical energetics, BSSE analysis, Cartesian coordinates, and structure-generation records

Contents

S1 Quantum-chemical energetic data **S5**
S1.1 Master reaction-coordinate energies . . . S5
S1.2 Absolute and relative energy inventory . . . S6
S1.3 Collision-model validation data . . . S9
S1.4 Data-consistency note . . . S9

S2 Proposed mechanisms for formation of *m/z* 117A and 117B **S10**

S3 Energy-resolved MS/MS data **S12**

S4 Individual energy-resolved MS/MS spectra **S15**

S5 Full-scan MS1 and pseudo-MS3 spectra **S35**

S6 Basis-set superposition error (BSSE) analysis **S40**
S6.1 Raw electronic energies . . . S40
S6.2 BSSE and counterpoise-corrected interaction energies . . . S40
S6.3 Reference . . . S41

S7 Overview of coordinate files **S42**

S8 Cartesian coordinates used in ORCA calculations **S43**
S8.1 135-117A concerted . . . S43
S8.2 135-117A stepwise . . . S45

S8.3 135-117B concerted S49
S8.4 135-117B stepwise S53
S8.5 135-59A S57
S8.6 135-59B S62
S8.7 135-77 . . . . S65

S9 ForMileS output used to generate molecular graphic structures **S67**
S9.1 ForMileS run summary: C3O (59.05 +/- 0.5) S67
S9.2 ForMileS run summary: C3O2 (77.06 +/- 0.5) S68
S9.3 ForMileS run summary: C6O2 (117.092 +/- 0.5) S69

List of Tables

S1 Master relative energies for all proposed PO3G-d dissociation pathways. All values are ΔE^0 in kJ mol^{-1} relative to 135 p2c1. . . . . S5

S2 Detailed energetic inventory used for the PO3G-d mechanistic analysis. SPE, ZPE, and E^0 are in Hartree (E_h); ΔE^0 is in kJ mol^{-1}. S7

S3 Collision-model conversion values. The last two columns reproduce the corresponding values after the internal energy increment. S9

S4 Initial internal-energy values as a function of temperature in the collision-model worksheet. S9

S5 Fractional ion yields for the selected ions as a function of absolute HCD collision voltage. S12

S6 Sensitivity of fractional ion yields at 6.9 V to the peak-picking prominence threshold. S12

S7 Fractional ion yields interpolated at the precursor transition midpoint (6.9 V). . . . S13

S8 Post-maximum half-decay collision energies for fragment-ion fractional-yield curves. . S13

S9 Ion injection-time summary for the 1–20 V absolute-HCD series. S13

S10 Operational energy metrics used in the interpretation of the selected precursor and fragment ions. S14

S11 Electronic energies used in the Boys–Bernardi counterpoise analysis. S40

S12 BSSE contributions and interaction energies in kJ mol^{-1}. S41

S13 Overview of Cartesian-coordinate files and associated ORCA electronic energies. . . S42

S14 Summary of ForMileS molecular-graph generation records used for the selected product-ion formulas. S67

List of Figures

S1 Proposed dissociation mechanism connecting the precursor ion at m/z 135 with product ion 117A. Panel (a) shows the computed reaction-coordinate profile and panel (b) shows the corresponding curly-arrow mechanism. S10

S2 Proposed dissociation mechanism connecting the precursor ion at m/z 135 with product ion 117B. Panel (a) shows the computed reaction-coordinate profile and panel (b) shows the corresponding curly-arrow mechanism. S11

S3 ERMS spectrum at an absolute HCD collision voltage of 1 V. S15

S4 ERMS spectrum at an absolute HCD collision voltage of 2 V. S16

S5 ERMS spectrum at an absolute HCD collision voltage of 3 V. S17

S6 ERMS spectrum at an absolute HCD collision voltage of 4 V. S18

S7 ERMS spectrum at an absolute HCD collision voltage of 5 V. S19

S8 ERMS spectrum at an absolute HCD collision voltage of 6 V. S20

S9 ERMS spectrum at an absolute HCD collision voltage of 7 V. S21

S10 ERMS spectrum at an absolute HCD collision voltage of 8 V. S22

S11 ERMS spectrum at an absolute HCD collision voltage of 9 V. S23

S12 ERMS spectrum at an absolute HCD collision voltage of 10 V. S24

S13 ERMS spectrum at an absolute HCD collision voltage of 11 V. S25

S14 ERMS spectrum at an absolute HCD collision voltage of 12 V. S26

S15 ERMS spectrum at an absolute HCD collision voltage of 13 V. S27

S16 ERMS spectrum at an absolute HCD collision voltage of 14 V. S28

S17 ERMS spectrum at an absolute HCD collision voltage of 15 V. S29

S18 ERMS spectrum at an absolute HCD collision voltage of 16 V. S30

S19 ERMS spectrum at an absolute HCD collision voltage of 17 V. S31

S20 ERMS spectrum at an absolute HCD collision voltage of 18 V. S32

S21 ERMS spectrum at an absolute HCD collision voltage of 19 V. S33

S22 ERMS spectrum at an absolute HCD collision voltage of 20 V. S34

S23 Full-scan MS1 spectrum. S36

S24 Pseudo-MS3 spectrum of m/z 77 at 4 eV. S37

S25 Pseudo-MS3 spectrum of m/z 89 at 4 eV. S38

S26 Pseudo-MS3 spectrum of m/z 117 at 4 eV. S39

S1 Quantum-chemical energetic data

S1.1 Master reaction-coordinate energies

Table S1: Master relative energies for all proposed PO3G-d dissociation pathways. All values are ΔE^0 in kJ mol^{-1} relative to 135 p2c1.

**Pathway**	**State**	**Description**	ΔE^0
m/z 117A – concerted pathway	135 p2c1	Reference precursor conformer	0.000
	135 p1c2	Precursor protomer/conformer	12.982
	TS1	Concerted E2 dissociation	164.702
	117$\cdots H_2O$ (RC)	Post-transition-state reaction complex	41.648
	117A + H_2O	Separated product state	70.853
m/z 117A – stepwise pathway	135 p2c1	Reference precursor conformer	0.000
	135 p1c1	Precursor protomer/conformer	3.518
	TS1	1,2-H shift + epoxide-ring formation	177.362
	Int1$\cdots H_2O$ (RC)	Epoxide reaction complex	104.849
	Int1 + H_2O	Separated epoxide intermediate	126.693
	TS2	Ring opening / 1,2-H shift	209.687
	117A + H_2O	Separated product state	68.644
m/z 117B – concerted formation of 117A followed by isomerization	135 p2c1	Reference precursor conformer	0.000
	135 p1c2	Precursor protomer/conformer	12.982
	TS1	Concerted E2 dissociation	164.702
	117$\cdots H_2O$ (RC)	Post-transition-state reaction complex	41.648
	117A + H_2O	First separated 117 isomer	70.853
	TS2	1,2-H shift (π-bond migration)	391.927
	117B + H_2O	Secondary-alkene protonated ether	65.227
m/z 117B – stepwise pathway followed by isomerization	135 p2c1	Reference precursor conformer	0.000
	135 p1c1	Precursor protomer/conformer	3.518
	TS1	1,2-H shift + epoxide-ring formation	177.362
	Int1$\cdots H_2O$ (RC)	Epoxide reaction complex	104.849

Continued on next page

Table S1 continued

**Pathway**	**State**	**Description**	ΔE^0
	Int1 + H_2O	Separated epoxide intermediate	126.693
	TS2	Ring opening / 1,2-H shift	209.687
	117A + H_2O	First separated 117 isomer	68.644
	TS3	1,2-H shift (π-bond migration)	391.927
	117B + H_2O	Secondary-alkene protonated ether	65.227
m/z 77 pathway	135 p2c1	Reference precursor conformer	0.000
	TS1	Dissociation + migration	176.674
	$77 \cdots$ NL (RC)	Protonated-monomer reaction complex	46.895
	77 + NL	Separated product state	121.932
m/z 59A pathway	135 p2c1	Reference precursor conformer	0.000
	135 p2c2	Precursor protomer/conformer	15.255
	TS1	Dissociation + epoxide-ring formation	187.266
	Int1$\cdots$NL (RC)	Protonated-epoxide reaction complex	59.406
	Int1 + NL	Separated epoxide intermediate	209.999
	TS2	Ring opening	270.811
	Int2 + NL	Secondary carbocation intermediate	225.365
	TS3	1,2-hydride shift	293.150
	59A + NL	Allylic-alcohol ion product	192.617
m/z 59B pathway	135 p2c1	Reference precursor conformer	0.000
	135 p2c2	Precursor protomer/conformer	15.255
	TS1	Dissociation + epoxide-ring formation	187.266
	Int1$\cdots$NL (RC)	Protonated-epoxide reaction complex	59.406
	Int1 + NL	Separated epoxide intermediate	209.999
	TS2	Ring opening	347.883
	59B + NL	Protonated ketone-oxonium product	68.454

S1.2 Absolute and relative energy inventory

Table S2: Detailed energetic inventory used for the PO3G-d mechanistic analysis. SPE, ZPE, and E^0 are in Hartree (E_h); ΔE^0 is in kJ mol^{-1}.

**Channel**	**Species**	**SPE (E_h)**	**ZPE (E_h)**	E^0 (E_h)	ΔE^0	**Use**
PO3G-d Protomer 2 Conformer 1	135 p2c1	-462.579904732178	0.21610564	-462.36379910	0.000	117A-C, 117A-S, 117B-C, 117B-S, 77, 59A, 59B
PO3G-d Protomer 1 Conformer 2	135 p1c2	-462.573600351220	0.21474568	-462.35885470	12.982	117A-C, 117B-C
Dissociation (E2, concerted)	TS1	-462.510162853302	0.20909531	-462.30106750	164.702	117A-C, 117B-C
Protonated Allylic Alcohol (RC)	117—H2O (RC)	-462.557432344351	0.20949624	-462.34793610	41.648	117A-C, 117B-C
Neutral Loss - Water	H2O	-76.376607676615	0.02114499	-76.35546269	–	117A-C, 117A-S, 117B-C, 117B-S
Protonated Allylic Alcohol (Alone)	117 A	-386.168984362789	0.18763454	-385.98134980	–	117A-C, 117B-C
Protonated Allylic Alcohol - Protomer 1 + H2O	117 A + [H2O]	-462.545592000000	0.20877953	-462.33681250	70.853	117A-C, 117B-C
PO3G-d Protomer 1 Conformer 1	135 p1c1	-462.579636005228	0.21717679	-462.36245920	3.518	117A-S, 117B-S
Dissociation (1,2-H Shift + epoxide ring)	TS1	-462.504199145504	0.20795352	-462.29624560	177.362	117A-S, 117B-S
Epoxide Ring (RC)	Int1—NL (RC)	-462.536170871767	0.21230682	-462.32386410	104.849	117A-S
Epoxide Ring (Alone)	Int1	-386.147606578599	0.18752507	-385.96008150	–	117A-S, 117B-S
Epoxide Ring + H2O	Int1 + [NL]	-462.524214300000	0.20867006	-462.31554420	126.693	117A-S, 117B-S
Ring opening (1,2H-shift) + H2O	TS2	-462.488219100000	0.20428543	-462.28393360	209.687	117A-S, 117B-S
Protonated Allylic Alcohol - Protomer 2 + H2O	117A + [H2O]	-462.546725000000	0.20907110	-462.33765390	68.644	117A-S
1,2-H shift (pi-bond migration)	TS2	-462.415983900000	0.20146185	-462.21452210	391.927	117B-C, 117B-S
Secondary Alkene protonated ether	117B	-462.546935700000	0.20798012	-462.33895560	65.227	117B-C, 117B-S
Epoxide Ring (RC)	Int1—NL (RC)	-462.536170871767	0.21230682	-462.32386410	104.849	117B-S
Protonated Allylic Alcohol - Protomer 2 + H2O	117A	-462.546725000000	0.20907110	-462.33765390	68.644	117B-S
Dissociation + migration	TS1	-462.505762142065	0.20925462	-462.29650750	176.674	77
Protonated Monomer (RC)	77—NL (RC)	-462.558033847171	0.21209614	-462.34593770	46.895	77
Neutral Loss - 58 Da	NL	-192.895396488847	0.08450015	-192.81089630	–	77
Protonated Monomer (Alone)	77	-269.631041933806	0.12458081	-269.50646110	–	77
Protonated Monomer	77 + [NL]	-462.526438400000	0.20908096	-462.31735750	121.932	77
PO3G-d Protomer 2 Conformer 2	135 p2c2	-462.574536611310	0.21654794	-462.35798870	15.255	59A, 59B
Dissociation + epoxide ring formation	TS1	-462.502195635633	0.20972253	-462.29247310	187.266	59A, 59B
Protonated Epoxide Ring (RC)	Int1—NL (RC)	-462.553851554211	0.21267919	-462.34117240	59.406	59A, 59B
Neutral Loss - 76 Da	NL	-269.296614849046	0.11385182	-269.18276300	–	59A, 59B

Continued on next page

Table S2 continued

**Channel**	**Species**	**SPE (E_h)**	**ZPE (E_h)**	E^0 (E_h)	ΔE^0	**Use**
Protonated Epoxide Ring (Alone)	Int1	-193.198550892299	0.09749925	-193.10105160	–	59A, 59B
Protonated Epoxide Ring + NL	Int1 + [NL]	-462.495165700000	0.21135107	-462.28381470	209.999	59A, 59B
Ring opening + NL	TS2	-462.467711800000	0.20705901	-462.26065280	270.811	59A
Secondary Carbocation + NL	Int2 + [NL]	-462.484115500000	0.20615334	-462.27796220	225.365	59A
1,2-Hydride Shift + NL	TS3	-462.457085200000	0.20494108	-462.25214410	293.150	59A
Allylic Alcohol Ion + NL	59A + [NL]	-462.500839800000	0.21040449	-462.29043530	192.617	59A
Ring Opening + NL	TS2	-462.438293300000	0.20699568	-462.23129760	347.883	59B
Protonated Ketone Oxonium	59B + [NL]	-462.547395300000	0.20966910	-462.33772620	68.454	59B

S1.3 Collision-model validation data

Table S3: Collision-model conversion values. The last two columns reproduce the corresponding values after the internal energy increment.

**Scenario**	**135 Diss.**
$\eta = 1,\ Z = 100$	386.7
$\eta = 0.5,\ Z = 100$	213.6
$\eta = 1,\ Z = 3$	269.2
$\eta = 0.5,\ Z = 3$	147.9

Table S4: Initial internal-energy values as a function of temperature in the collision-model worksheet.

**Temperature (K)**	**Initial internal energy ($kJ\ mol^{-1}$)**	**Maximum increment ($kJ\ mol^{-1}$)**	**Maximum internal energy ($kJ\ mol^{-1}$)**
298.15	22.56	364.1	386.7
400	39.87	364.1	404.0
500	61.74	364.1	425.9
600	87.87	364.1	452.0
800	150.24	364.1	514.4
1000	222.91	364.1	587.1

S1.4 Data-consistency note

S2 Proposed mechanisms for formation of m/z 117A and 117B

The two mechanistic schemes below summarize the proposed pathways connecting the protonated precursor at m/z 135 with the two m/z 117 product-ion structures considered in the mechanistic analysis. In each figure, panel (a) presents the corresponding computed reaction-coordinate profile, whereas panel (b) presents the curly-arrow representation of the proposed elementary steps.

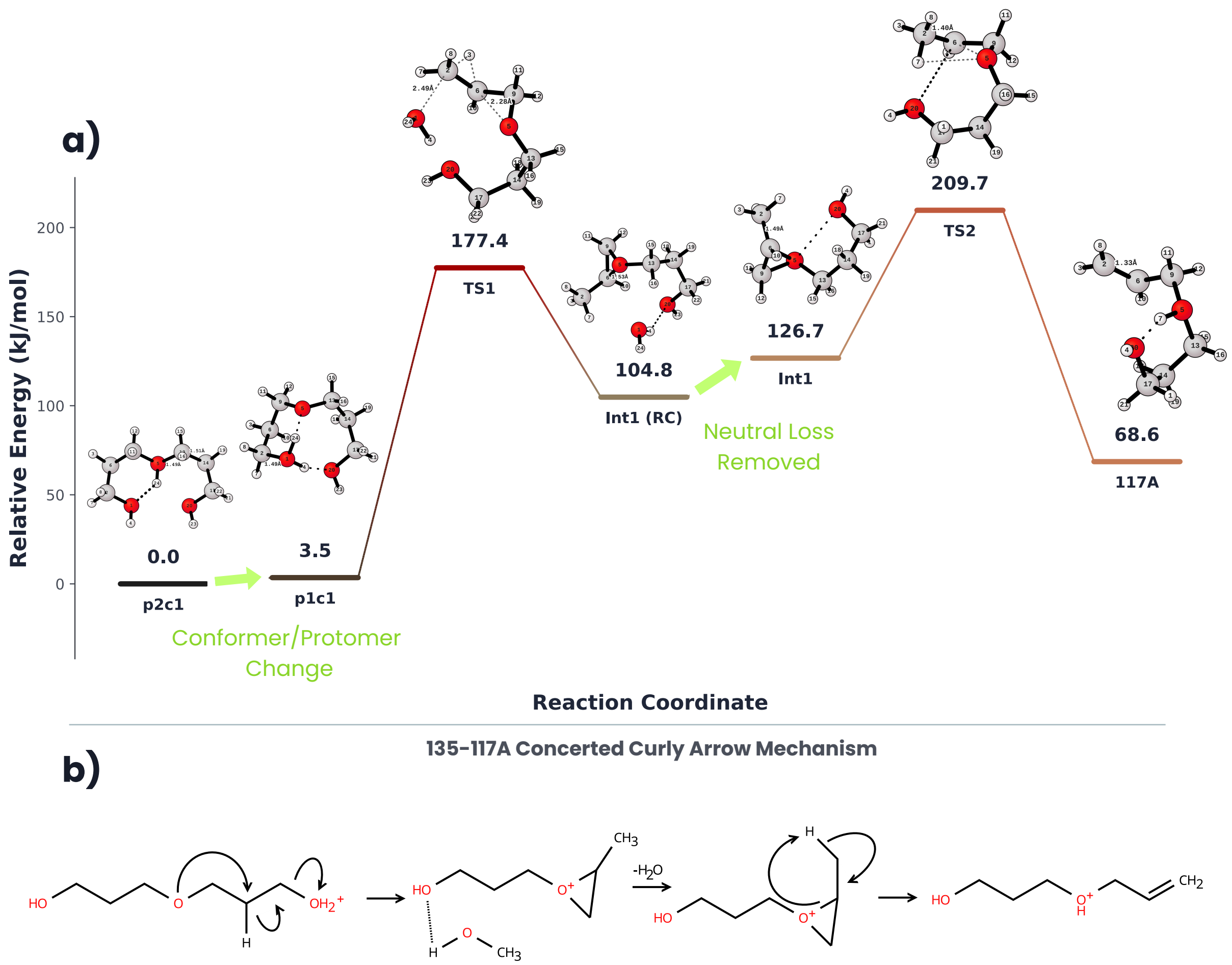

Figure S1: Proposed dissociation mechanism connecting the precursor ion at m/z 135 with product ion 117A. Panel (a) shows the computed reaction-coordinate profile and panel (b) shows the corresponding curly-arrow mechanism.

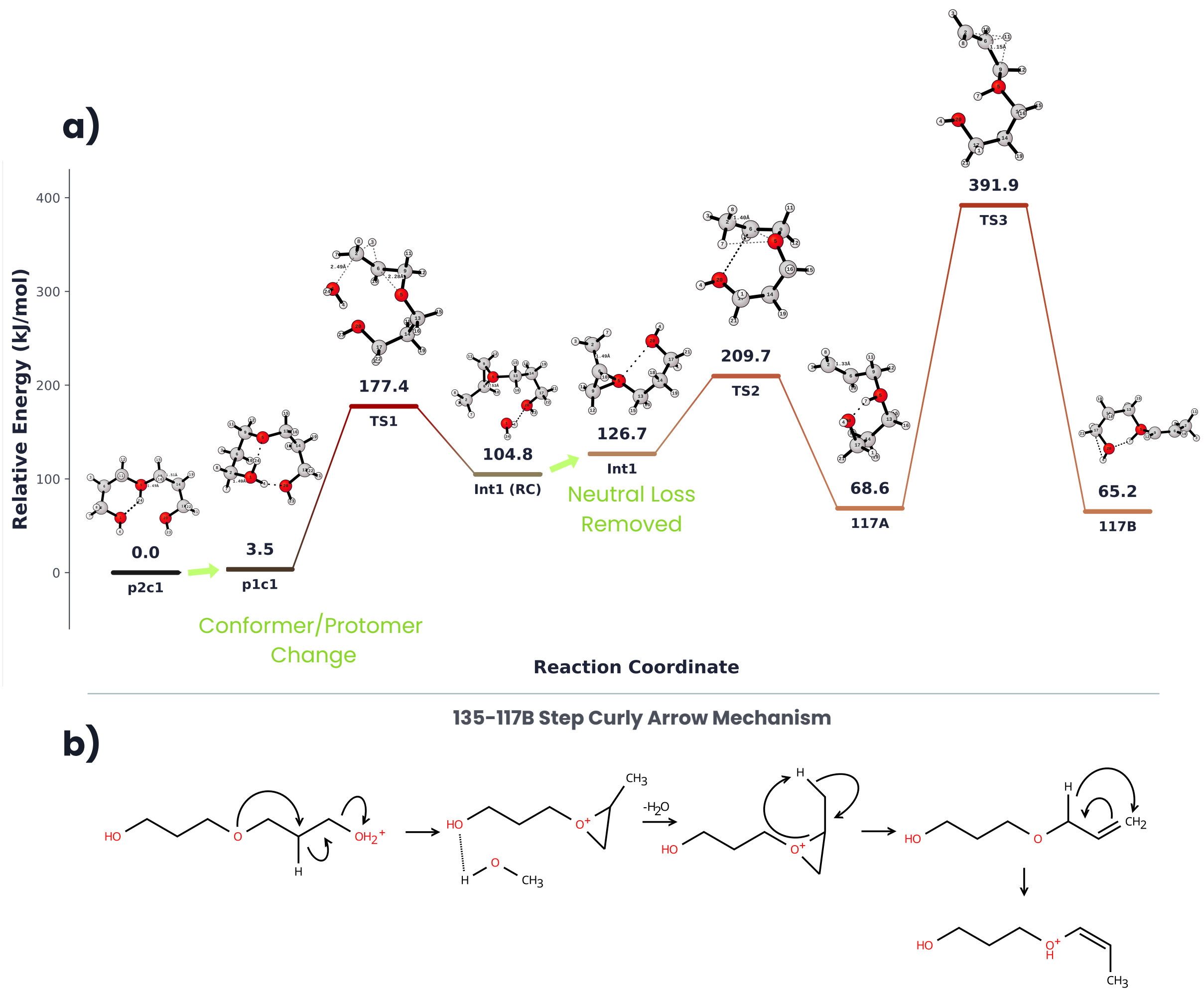

Figure S2: Proposed dissociation mechanism connecting the precursor ion at m/z 135 with product ion 117B. Panel (a) shows the computed reaction-coordinate profile and panel (b) shows the corresponding curly-arrow mechanism.

S3 Energy-resolved MS/MS data

Fractional ion yields were calculated as $Y_i = I_i / \sum_j I_j \times 100$, where the denominator corresponds to the summed intensity of all detected MS/MS ions after peak picking. Unless otherwise stated, the peak-picking prominence threshold was 0.5% of the base-peak intensity.

Table S5: Fractional ion yields for the selected ions as a function of absolute HCD collision voltage.

Voltage (V)	Y_{135} (%)	Y_{117} (%)	Y_{89} (%)	Y_{77} (%)	Y_{59} (%)
1	38.289	23.425	7.156	15.218	9.186
2	37.486	23.604	7.300	15.300	9.452
3	37.076	23.732	7.372	15.349	9.573
4	36.616	23.909	7.437	15.378	9.718
5	36.285	23.990	7.498	15.412	9.801
6	29.003	24.411	9.192	16.921	12.589
7	17.179	22.640	12.454	18.925	19.185
8	9.329	18.504	14.605	19.274	26.517
9	4.636	13.637	15.035	17.911	34.182
10	2.146	9.161	13.938	15.701	41.226
11	0.919	5.664	11.746	13.013	47.391
12	0.384	3.258	9.169	10.539	51.927
13	0.144	1.766	6.610	8.217	54.948
14	0.023	0.922	4.506	6.208	56.368
15	0.004	0.474	2.965	4.587	56.539
16	0.004	0.235	1.877	3.312	56.070
17	0.002	0.109	1.152	2.387	54.290
18	0.002	0.034	0.694	1.719	51.835
19	0.003	0.009	0.410	1.238	48.733
20	0.003	0.006	0.239	0.873	45.534

Table S6: Sensitivity of fractional ion yields at 6.9 V to the peak-picking prominence threshold.

Threshold (% BP)	Y_{135}	Y_{117}	Y_{89}	Y_{77}	Y_{59}	Sum selected
0.5	18.361	22.817	12.128	18.725	18.526	90.557
1.0	18.481	22.959	12.201	18.839	18.637	91.117
2.0	18.774	23.329	12.400	19.145	18.941	92.590
5.0	19.254	23.911	12.704	19.618	19.404	94.891

Table S7: Fractional ion yields interpolated at the precursor transition midpoint (6.9 V).

Ion (m/z)	Fractional yield (%)
89	12.128
135	18.361
59	18.526
77	18.725
117	22.817

Table S8: Post-maximum half-decay collision energies for fragment-ion fractional-yield curves.

Ion (m/z)	V_{max} (V)	Y_{max} (%)	$Y_{\mathrm{max}}/2$ (%)	$E_{1/2,\mathrm{decay}}$ (V)
59	15	56.539	28.270	> 20
77	8	19.274	9.637	12.388
89	9	15.035	7.517	12.645
117	6	24.411	12.206	9.320

Table S9: Ion injection-time summary for the 1–20 V absolute-HCD series.

Voltage (V)	Median IT (ms)	Mean IT (ms)	Min IT (ms)	Max IT (ms)
1	1.342	2.081	0.310	55.000
2	1.344	2.721	0.311	55.000
3	1.351	2.734	0.302	55.000
4	1.333	2.177	0.297	55.000
5	1.365	2.771	0.292	55.000
6	1.415	2.930	0.334	55.000
7	1.620	2.736	0.354	55.000
8	1.844	2.615	0.474	55.000
9	2.163	3.292	0.585	55.000
10	2.524	3.258	0.792	55.000
11	3.006	4.241	0.829	55.000
12	3.464	4.730	1.109	55.000
13	4.061	5.301	1.315	55.000
14	4.594	6.012	1.095	55.000
15	5.411	6.319	1.034	55.000
16	6.058	7.012	2.438	55.000
17	7.465	8.780	1.617	55.000
18	8.557	9.604	2.138	55.000
19	10.495	11.215	1.257	55.000
20	12.056	12.939	1.827	55.000

Table S10: Operational energy metrics used in the interpretation of the selected precursor and fragment ions.

Metric	Value	Interpretation
Precursor transition midpoint from FY/SY-style fit	6.9 V	Midpoint of precursor decline
Precursor half-intensity in base-peak-relative breakdown curve	7.65 V	50% of relative precursor intensity
Precursor half-intensity in absolute-intensity breakdown curve	6.64 V	50% of absolute precursor intensity range
$E_{1/2,\mathrm{decay}}$ for m/z 117	9.32 V	Post-maximum half-decay in fractional yield
$E_{1/2,\mathrm{decay}}$ for m/z 89	12.65 V	Post-maximum half-decay in fractional yield
$E_{1/2,\mathrm{decay}}$ for m/z 77	12.39 V	Post-maximum half-decay in fractional yield
$E_{1/2,\mathrm{decay}}$ for m/z 59	> 20 V	Half-decay not reached within the studied range

S4 Individual energy-resolved MS/MS spectra

Figures S3–S22 show the individual MS/MS spectra acquired across the 1–20 V absolute-HCD collision-voltage series used for the energy-resolved MS/MS analysis. Spectral intensities are presented relative to the base peak (100%).

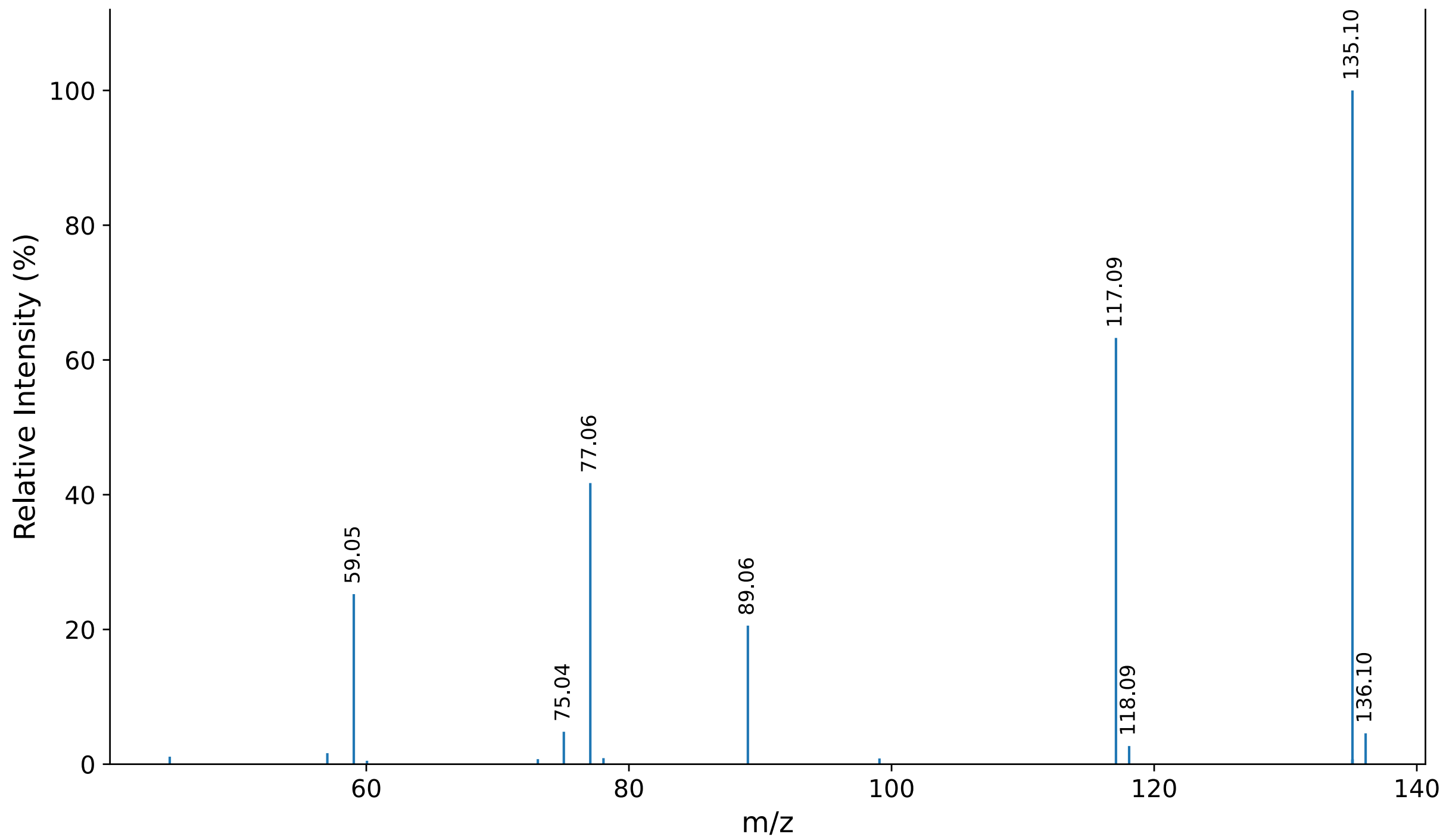

Figure S3: Energy-resolved MS/MS spectrum acquired at an absolute HCD collision voltage of 1 V. Intensities are reported relative to the base peak (100%).

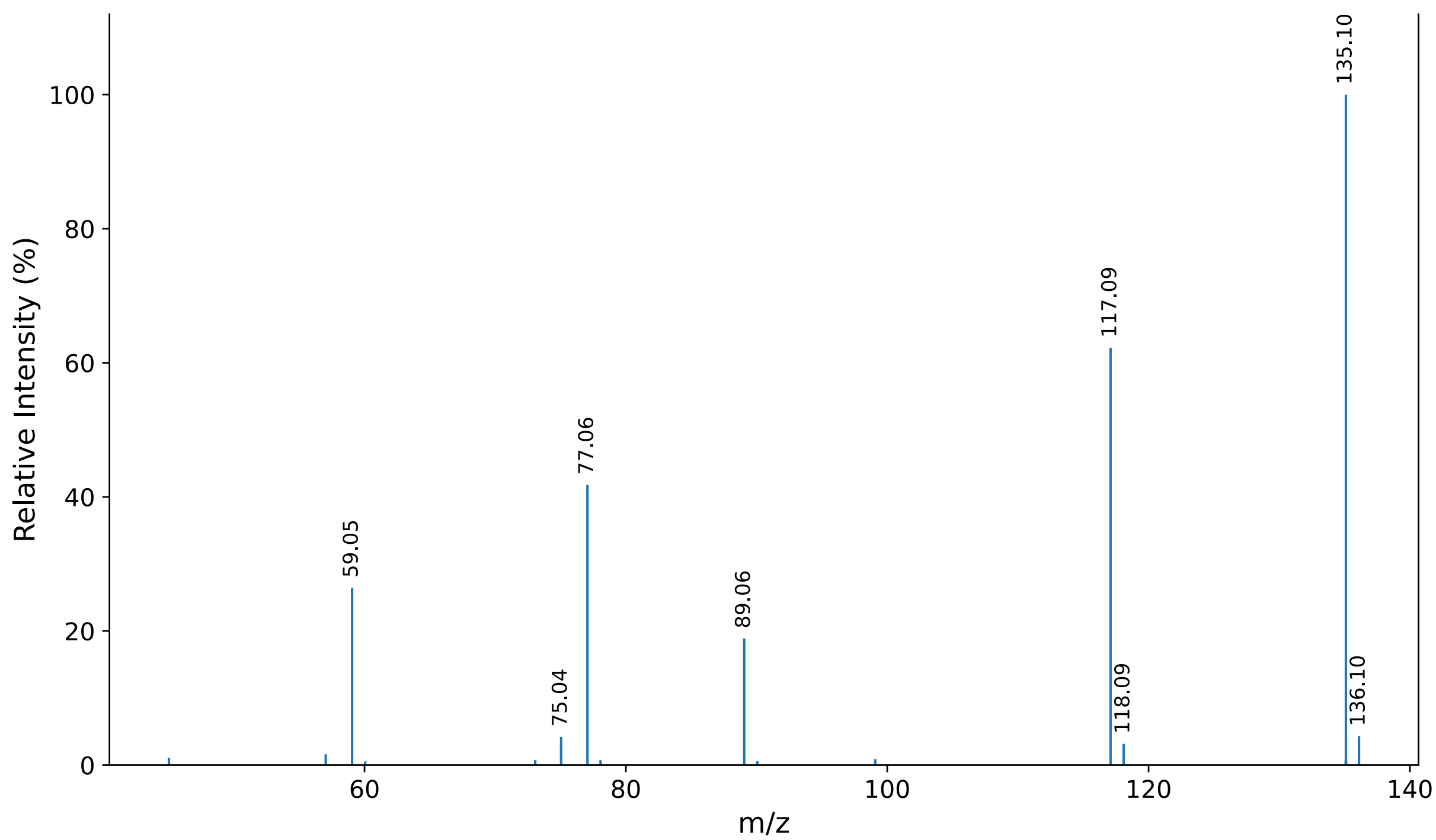

Figure S4: Energy-resolved MS/MS spectrum acquired at an absolute HCD collision voltage of 2 V. Intensities are reported relative to the base peak (100%).

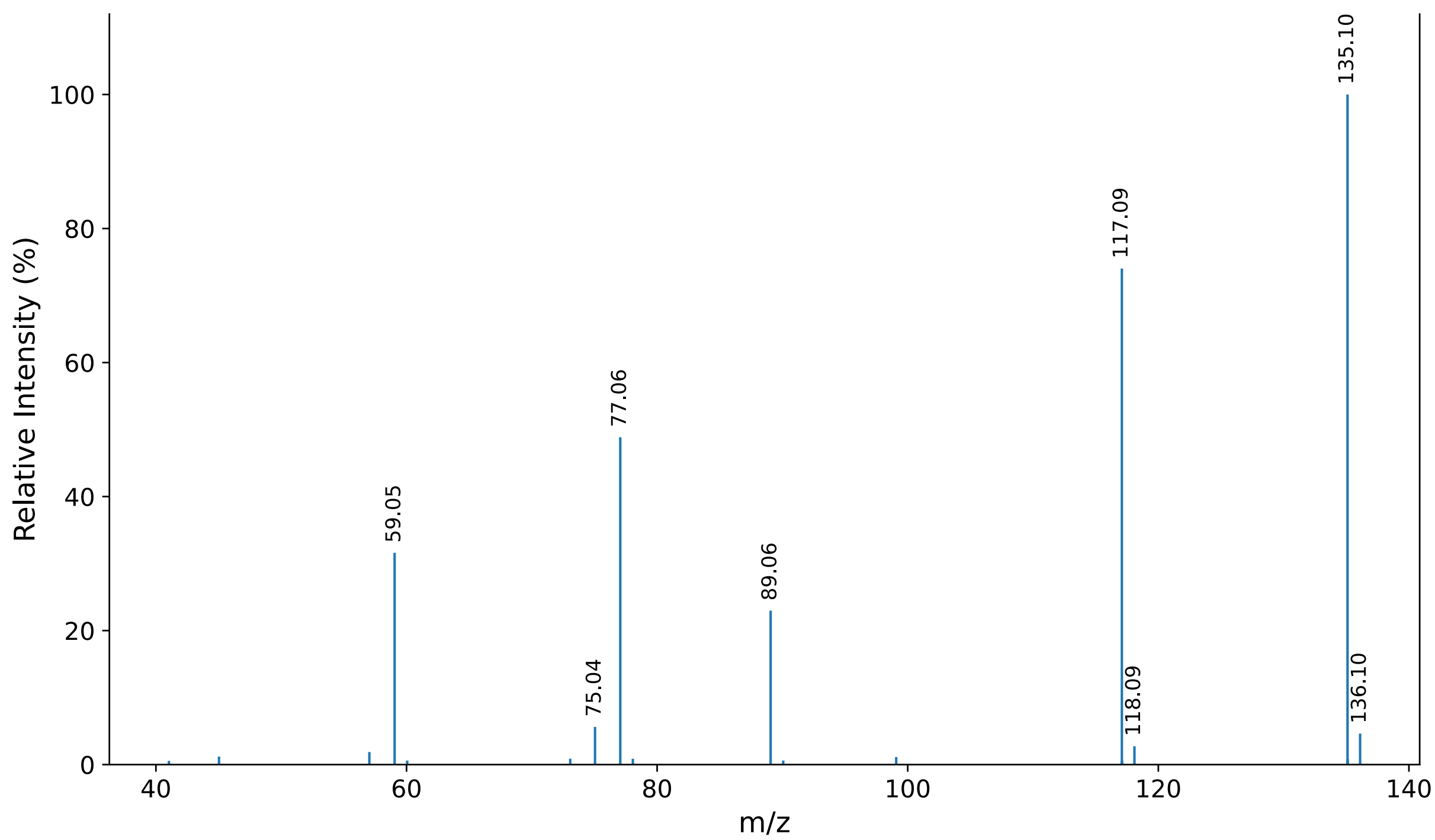

Figure S5: Energy-resolved MS/MS spectrum acquired at an absolute HCD collision voltage of 3 V. Intensities are reported relative to the base peak (100%).

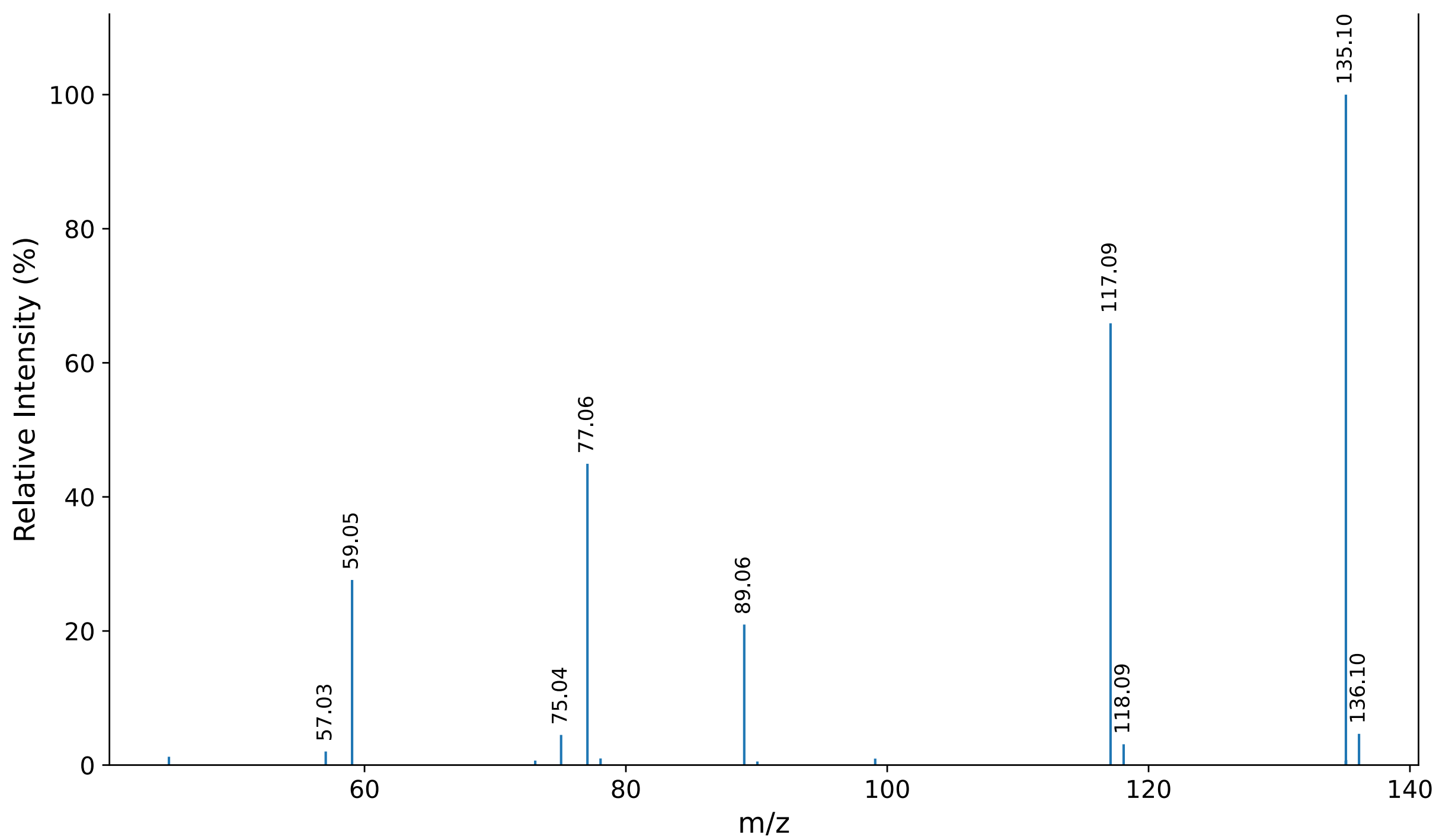

Figure S6: Energy-resolved MS/MS spectrum acquired at an absolute HCD collision voltage of 4 V. Intensities are reported relative to the base peak (100%).

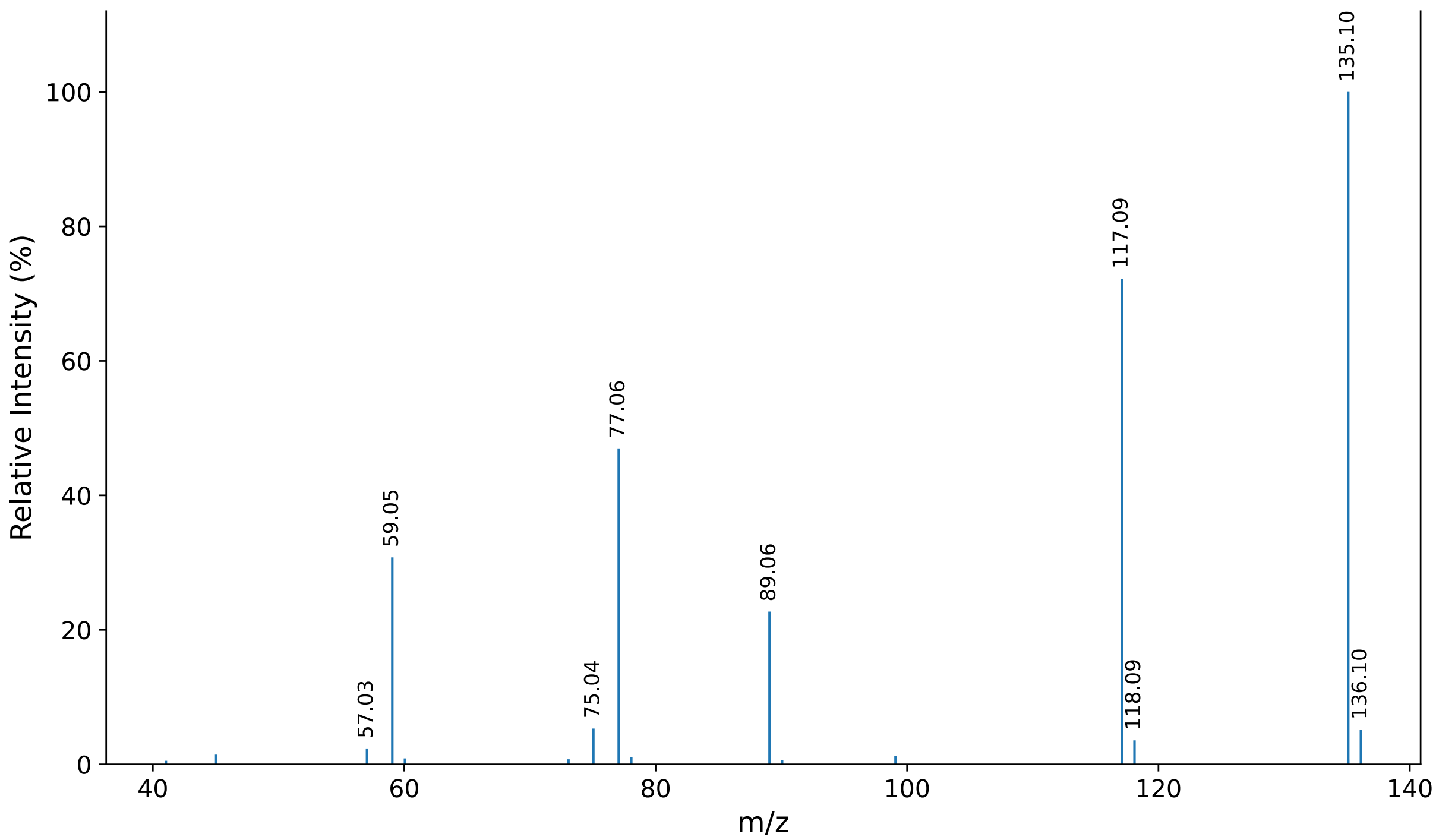

Figure S7: Energy-resolved MS/MS spectrum acquired at an absolute HCD collision voltage of 5 V. Intensities are reported relative to the base peak (100%).

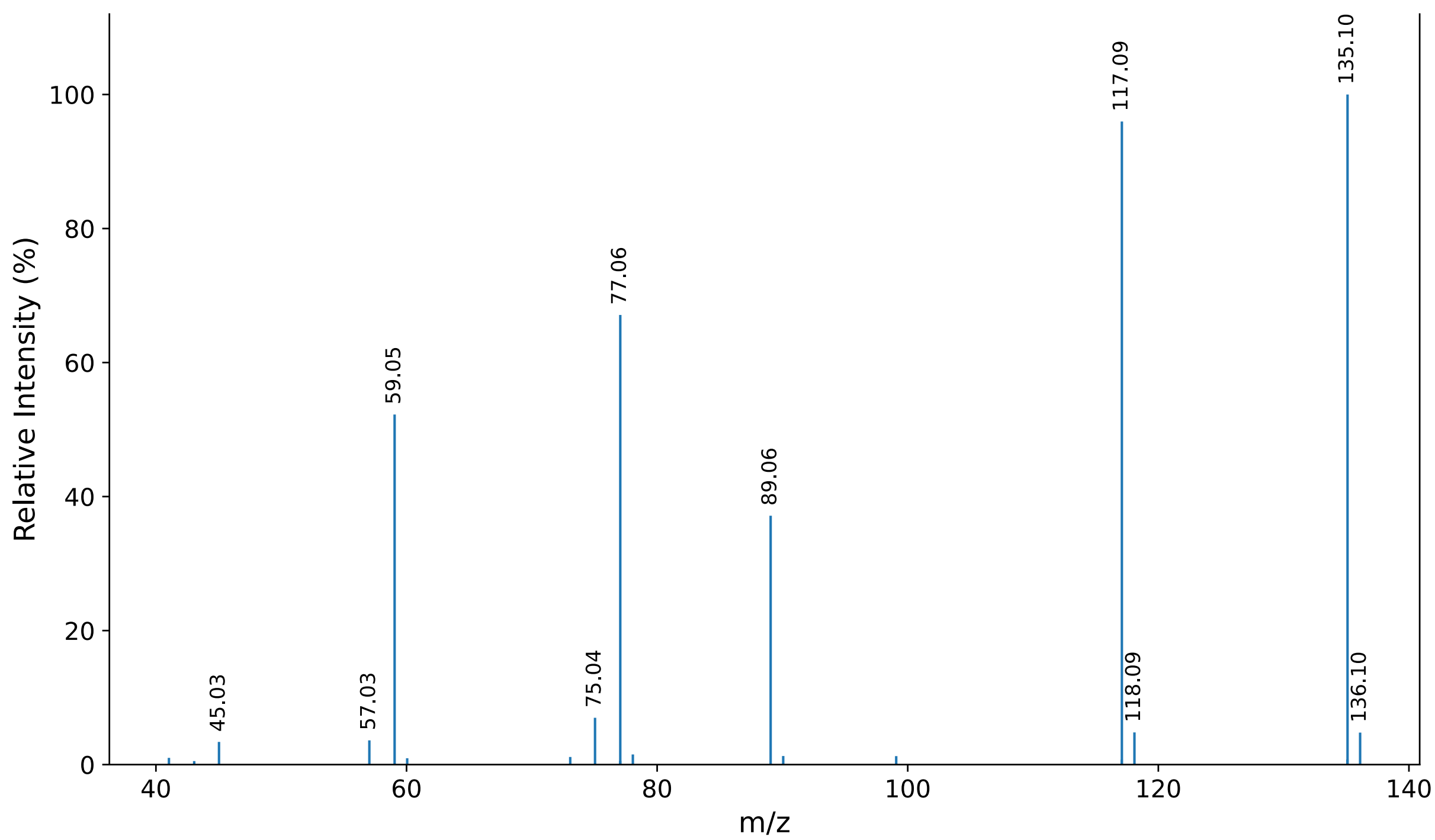

Figure S8: Energy-resolved MS/MS spectrum acquired at an absolute HCD collision voltage of 6 V. Intensities are reported relative to the base peak (100%).

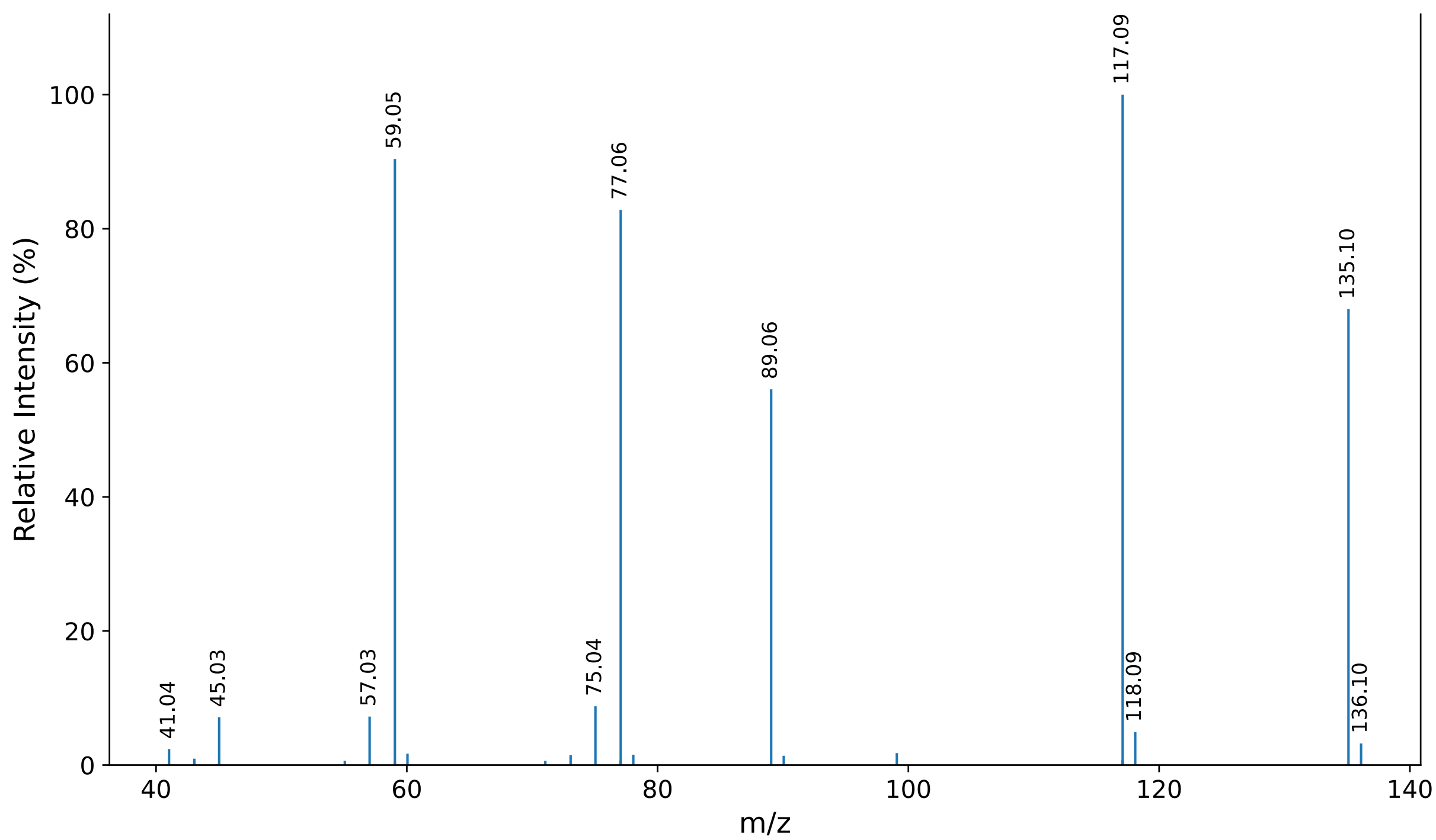

Figure S9: Energy-resolved MS/MS spectrum acquired at an absolute HCD collision voltage of 7 V. Intensities are reported relative to the base peak (100%).

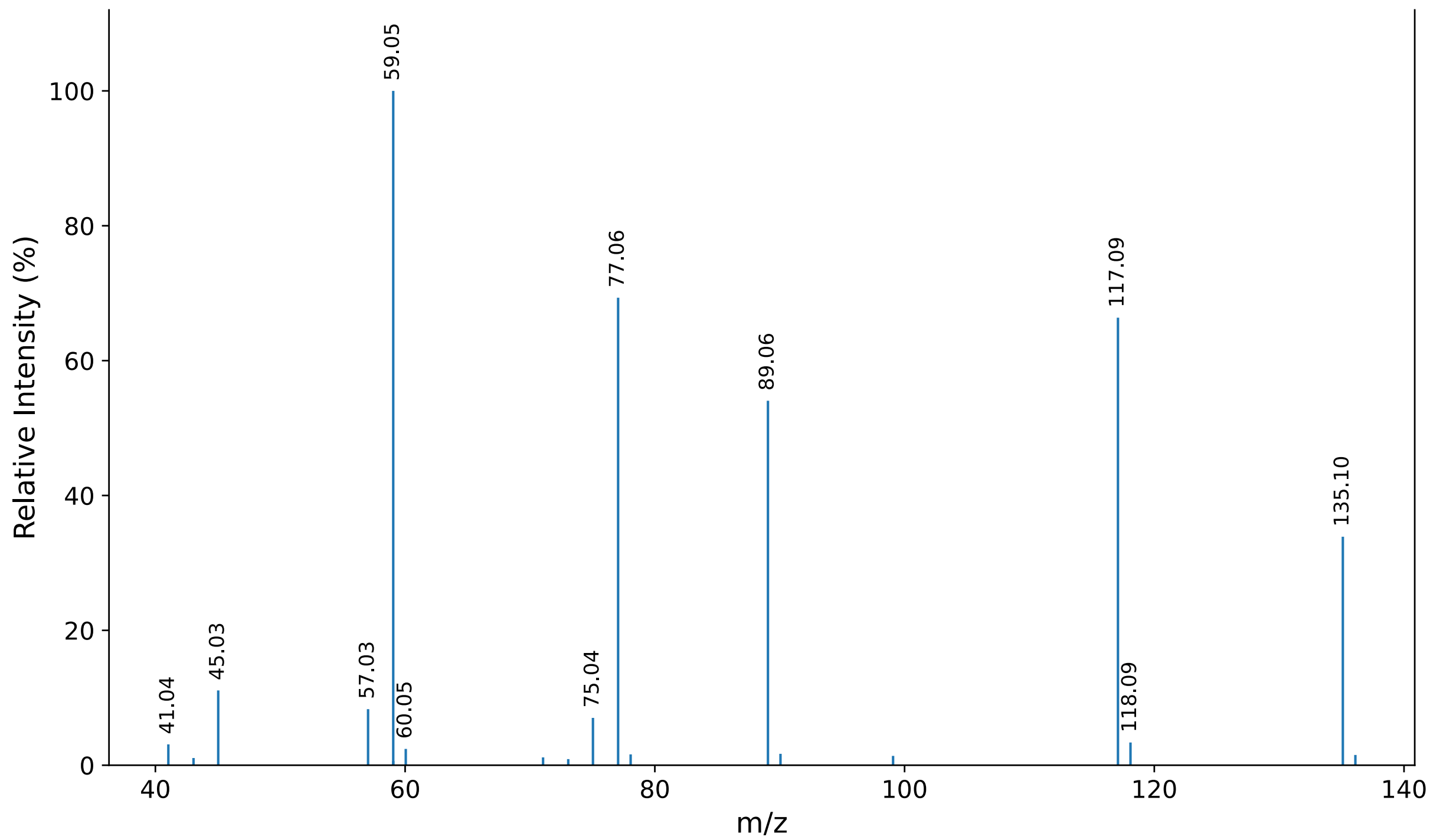

Figure S10: Energy-resolved MS/MS spectrum acquired at an absolute HCD collision voltage of 8 V. Intensities are reported relative to the base peak (100%).

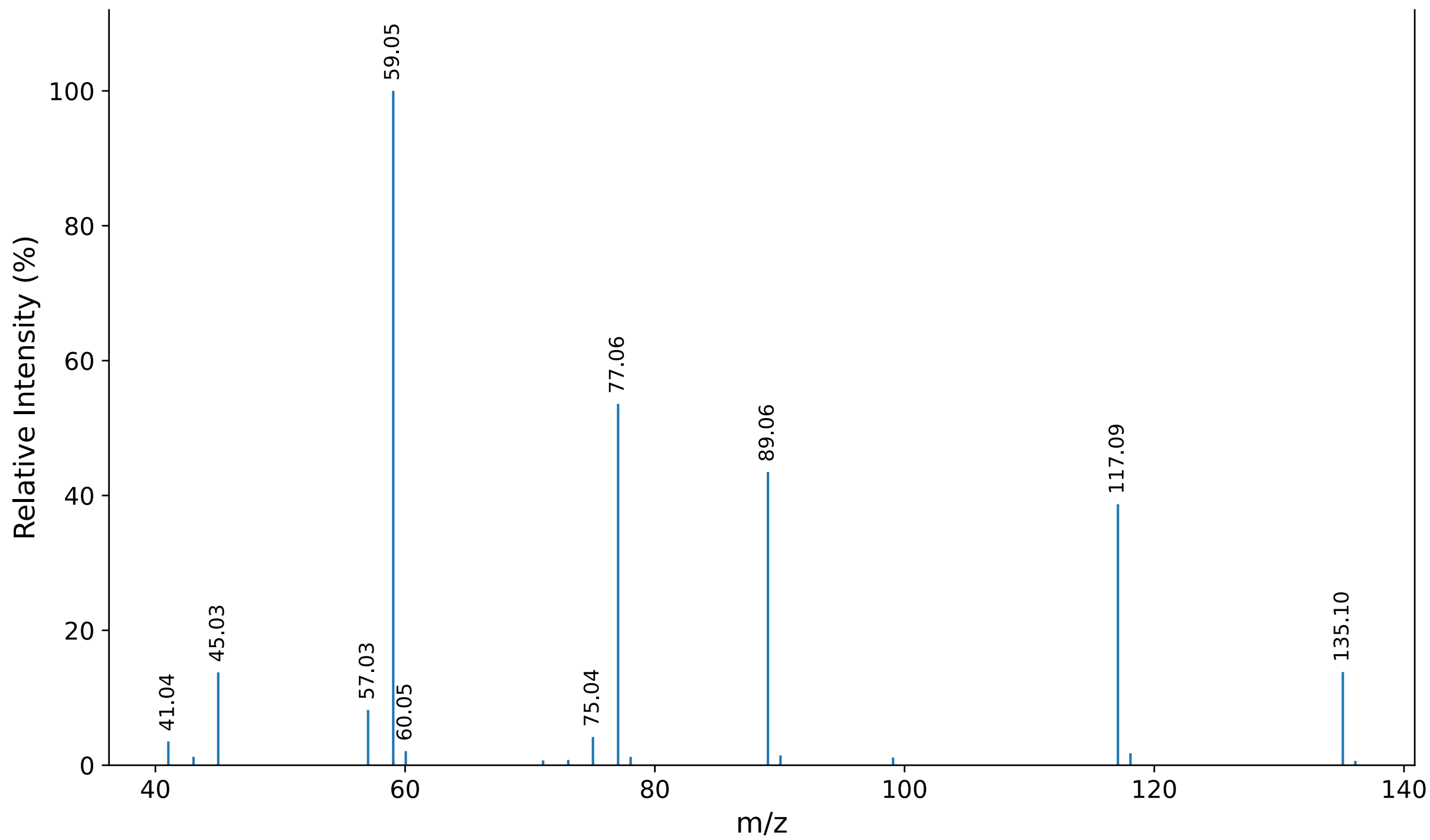

Figure S11: Energy-resolved MS/MS spectrum acquired at an absolute HCD collision voltage of 9 V. Intensities are reported relative to the base peak (100%).

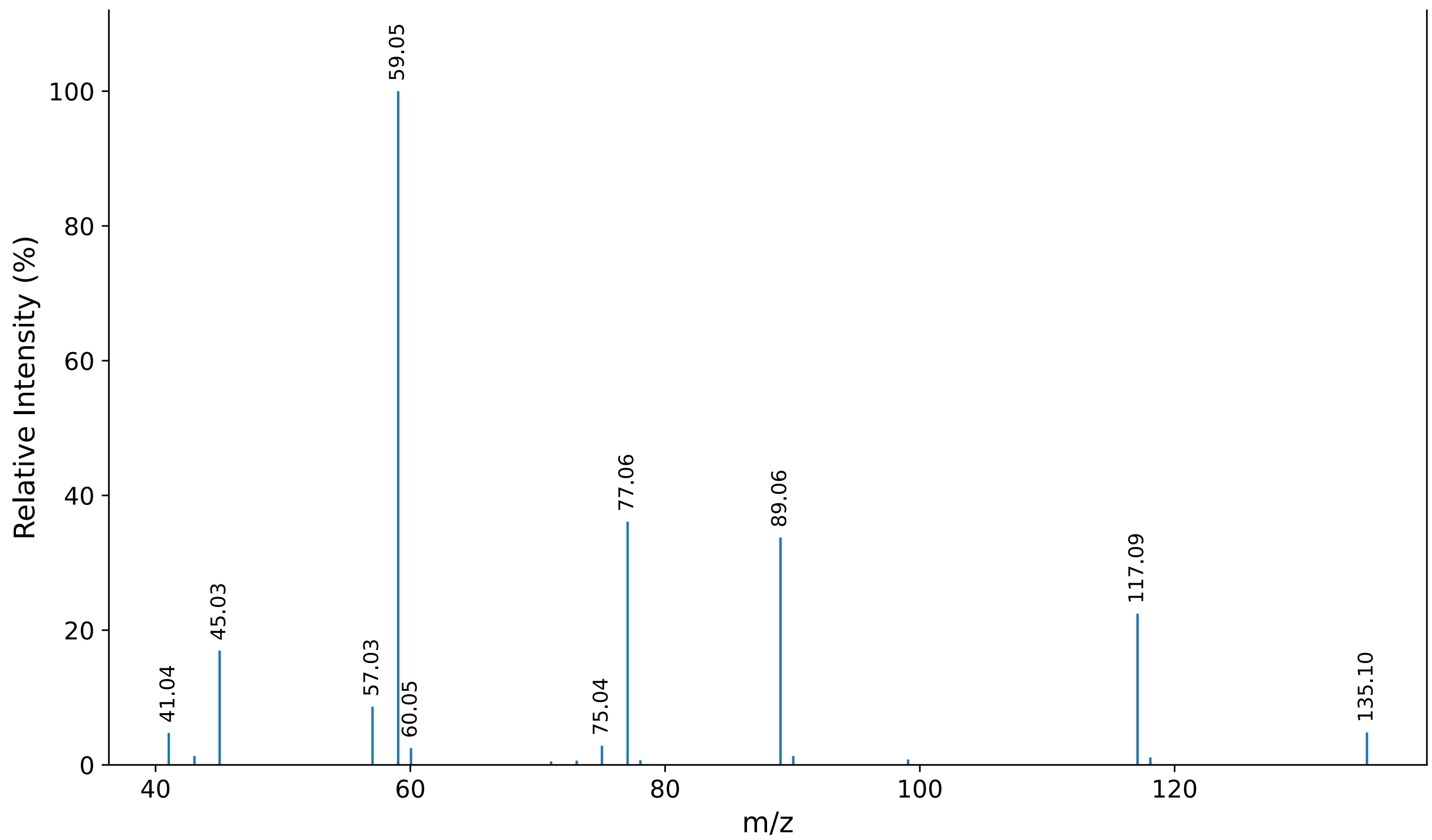

Figure S12: Energy-resolved MS/MS spectrum acquired at an absolute HCD collision voltage of 10 V. Intensities are reported relative to the base peak (100%).

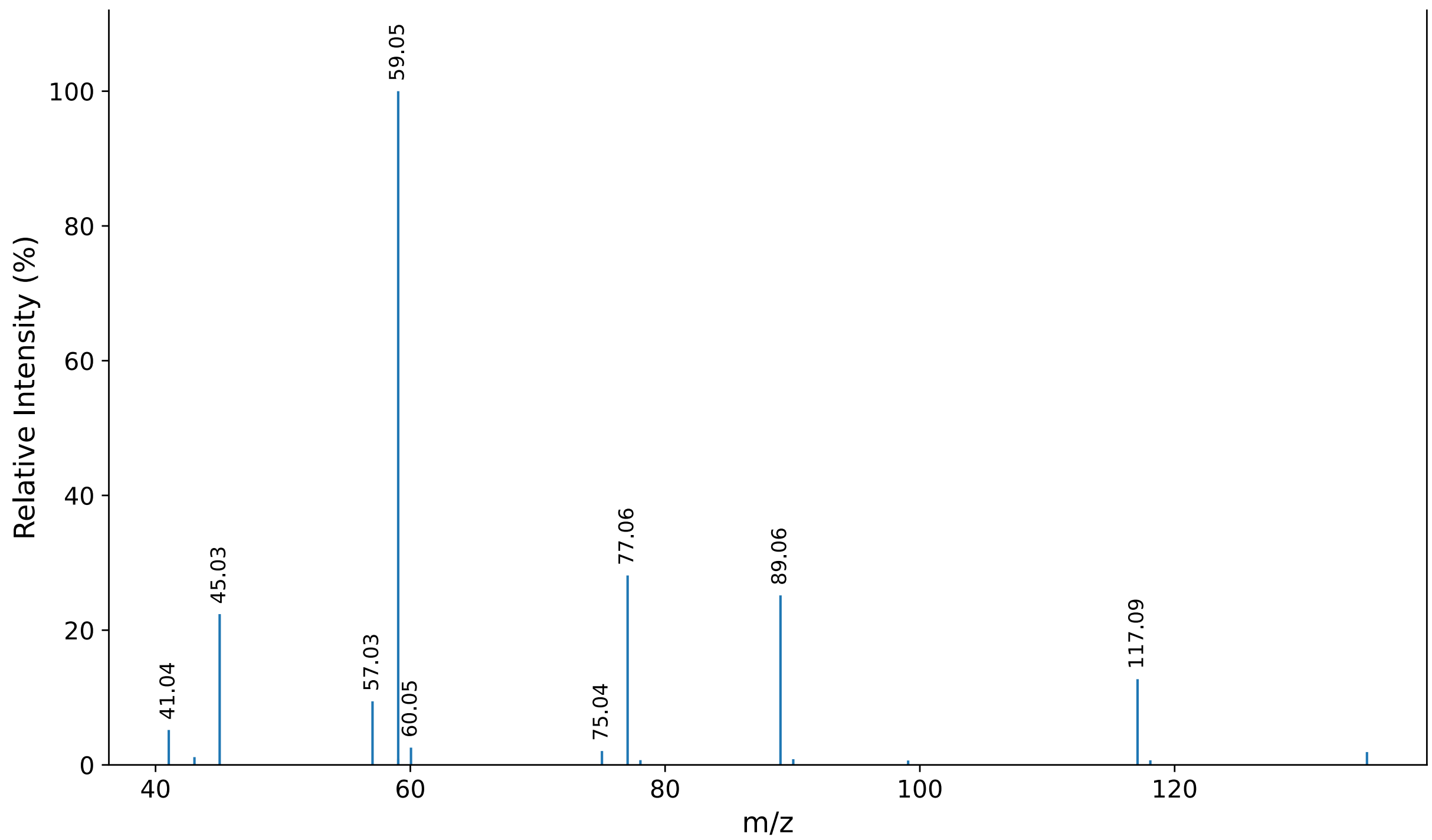

Figure S13: Energy-resolved MS/MS spectrum acquired at an absolute HCD collision voltage of 11 V. Intensities are reported relative to the base peak (100%).

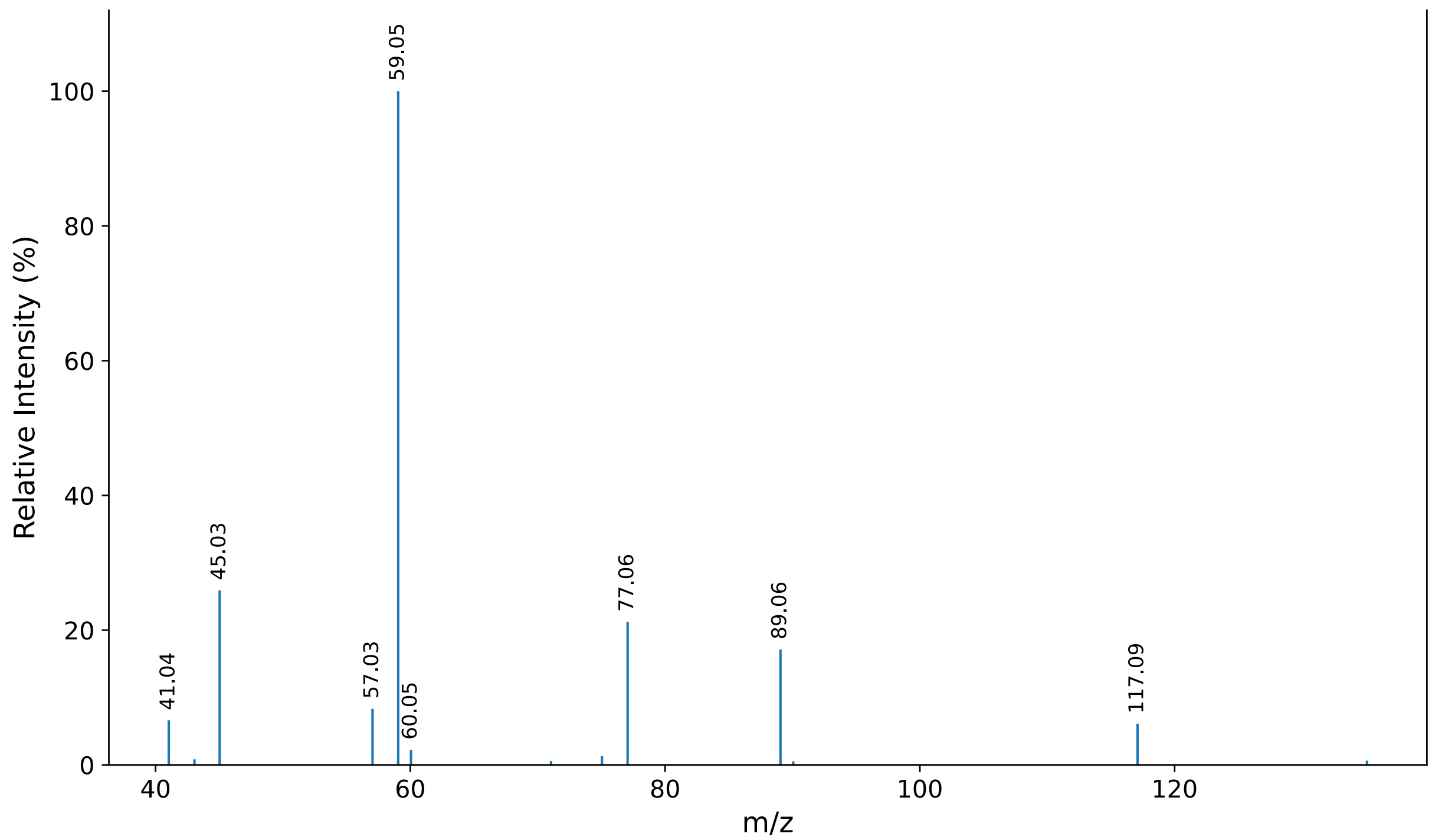

Figure S14: Energy-resolved MS/MS spectrum acquired at an absolute HCD collision voltage of 12 V. Intensities are reported relative to the base peak (100%).

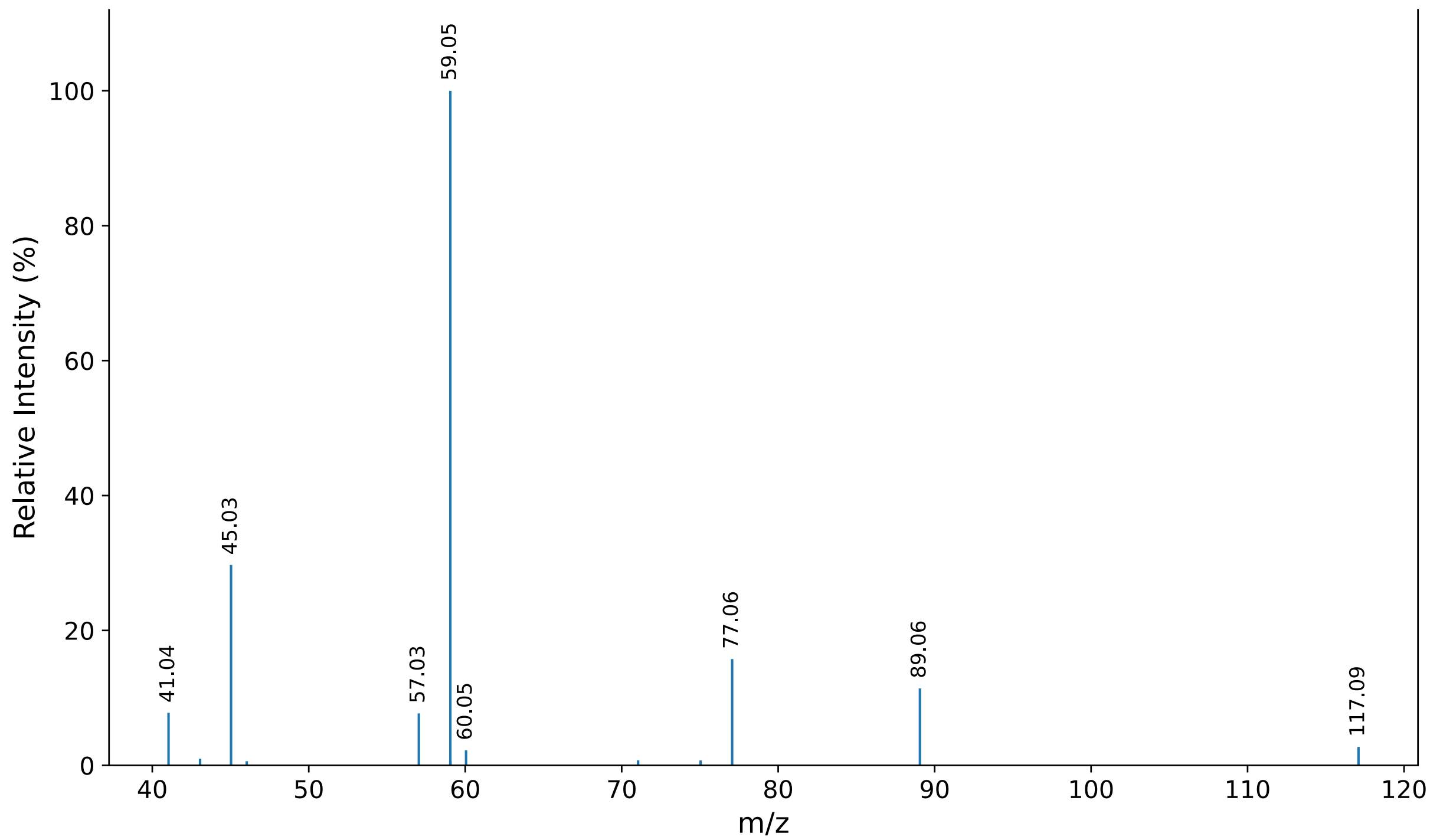

Figure S15: Energy-resolved MS/MS spectrum acquired at an absolute HCD collision voltage of 13 V. Intensities are reported relative to the base peak (100%).

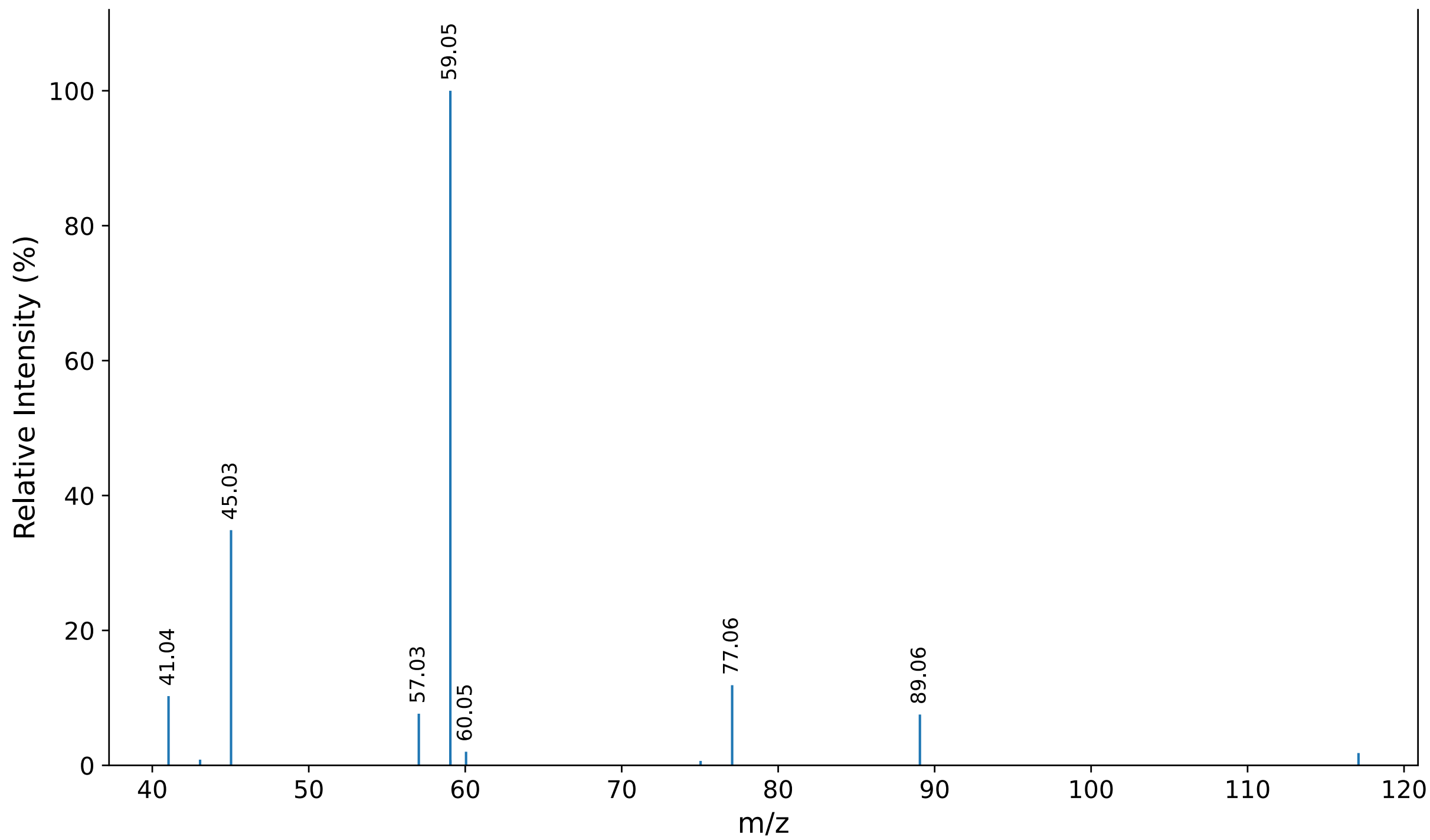

Figure S16: Energy-resolved MS/MS spectrum acquired at an absolute HCD collision voltage of 14 V. Intensities are reported relative to the base peak (100%).

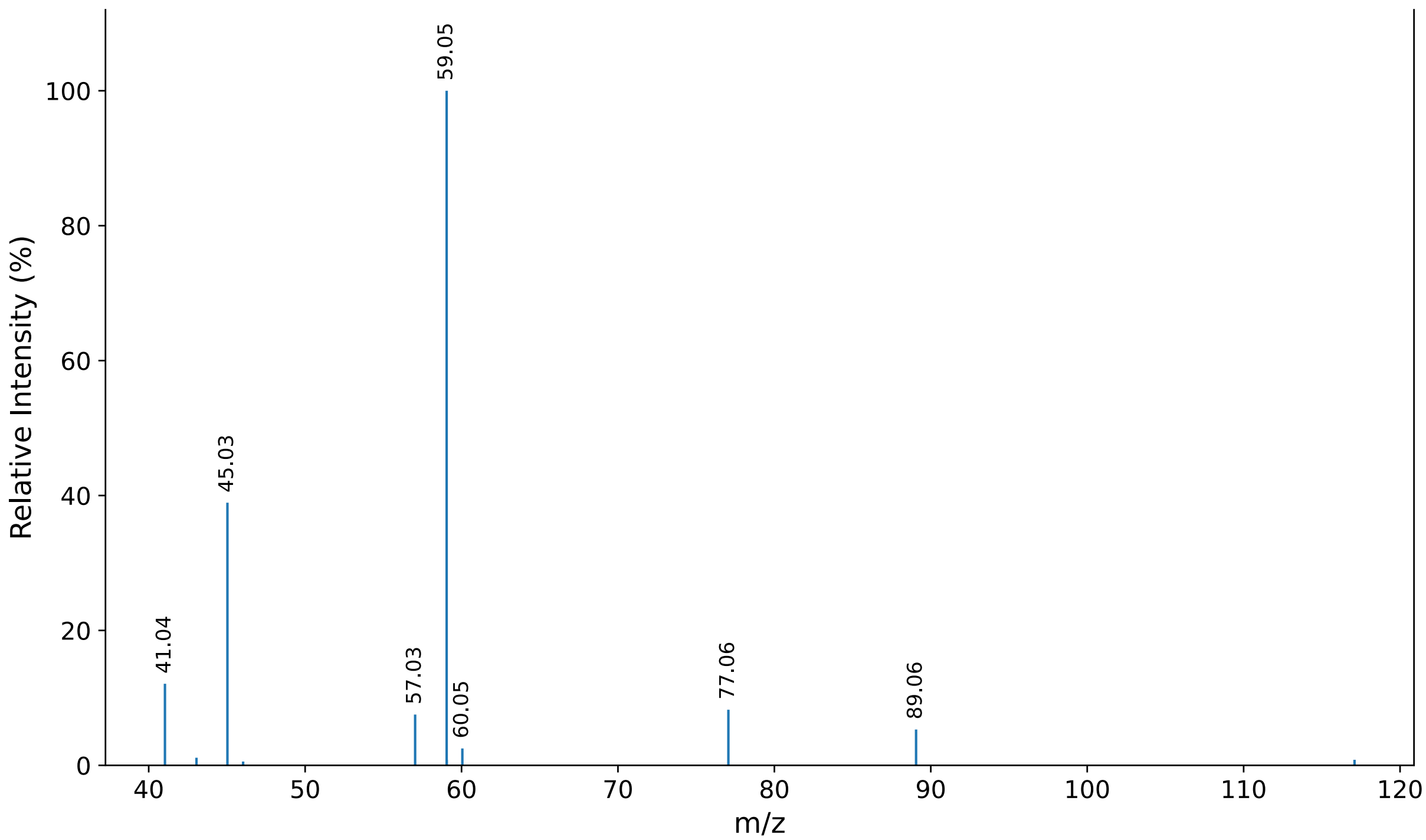

Figure S17: Energy-resolved MS/MS spectrum acquired at an absolute HCD collision voltage of 15 V. Intensities are reported relative to the base peak (100%).

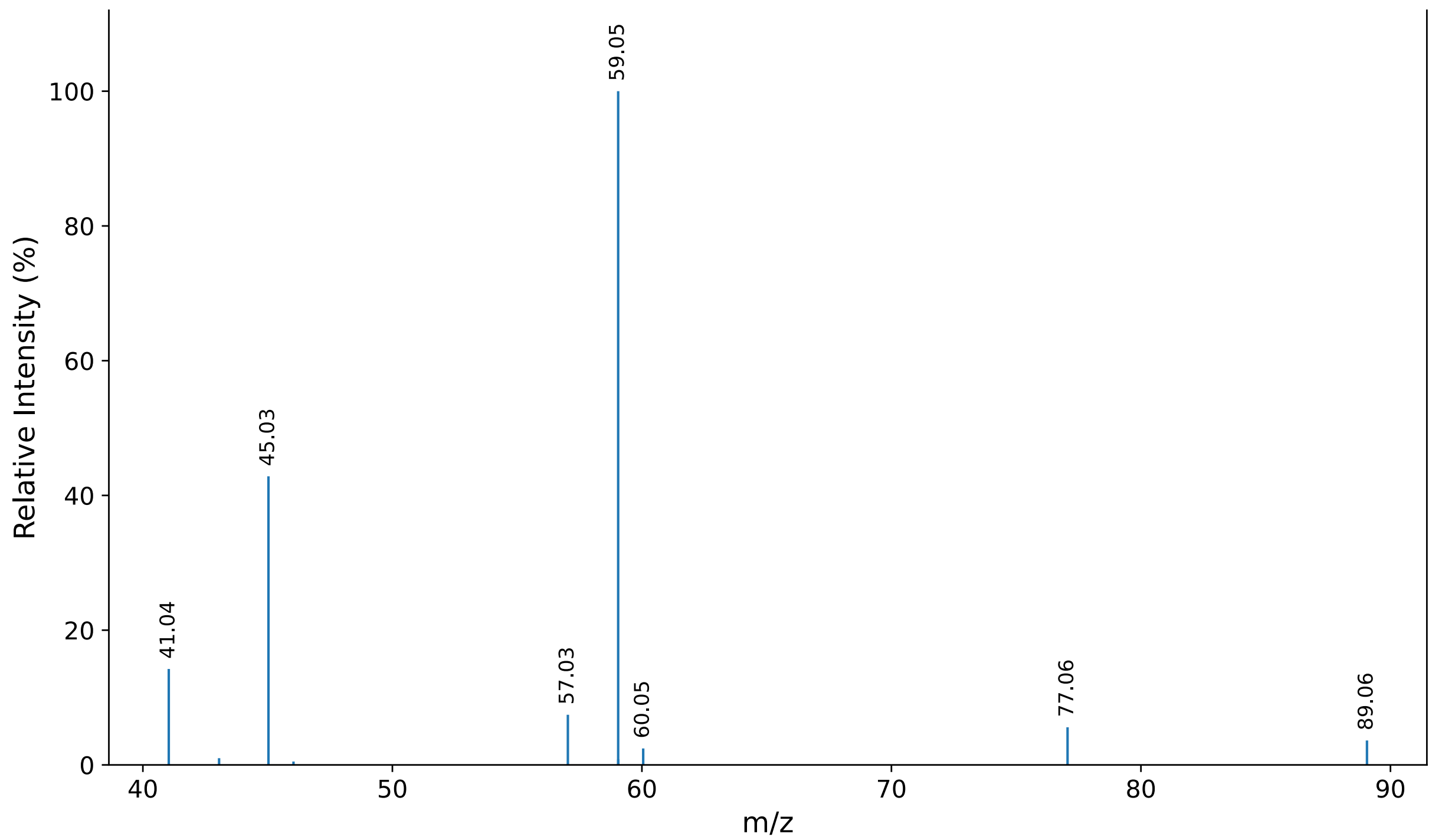

Figure S18: Energy-resolved MS/MS spectrum acquired at an absolute HCD collision voltage of 16 V. Intensities are reported relative to the base peak (100%).

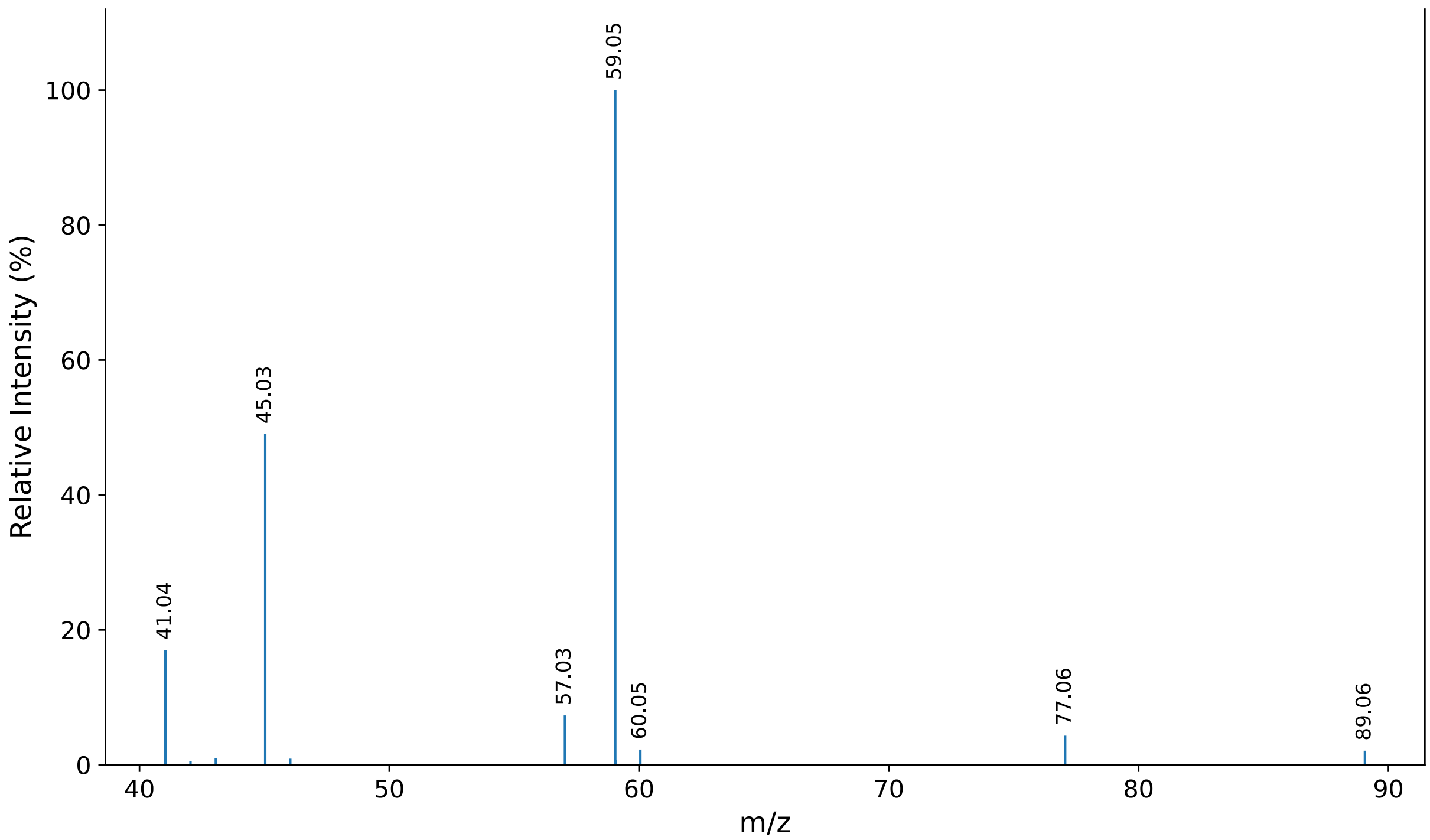

Figure S19: Energy-resolved MS/MS spectrum acquired at an absolute HCD collision voltage of 17 V. Intensities are reported relative to the base peak (100%).

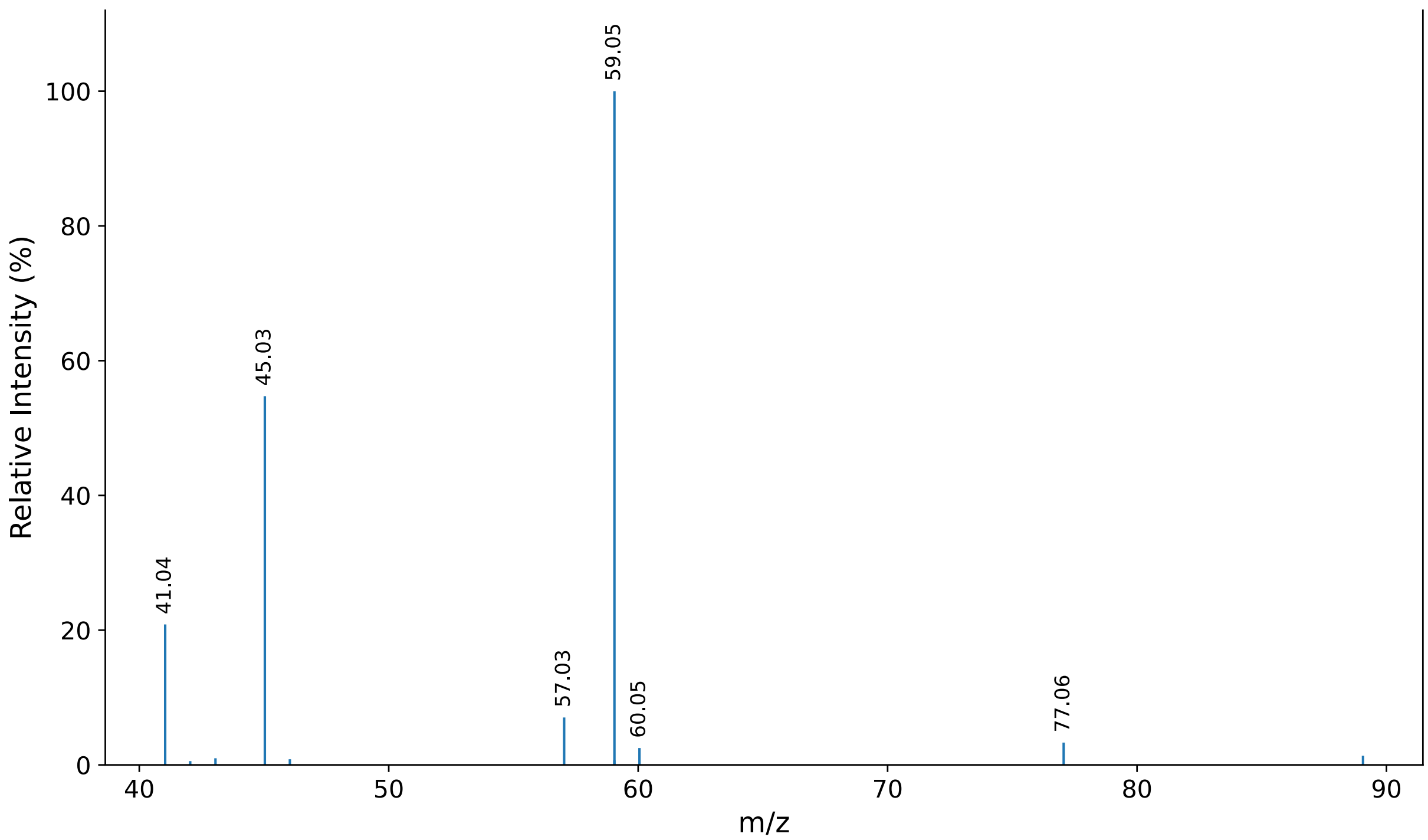

Figure S20: Energy-resolved MS/MS spectrum acquired at an absolute HCD collision voltage of 18 V. Intensities are reported relative to the base peak (100%).

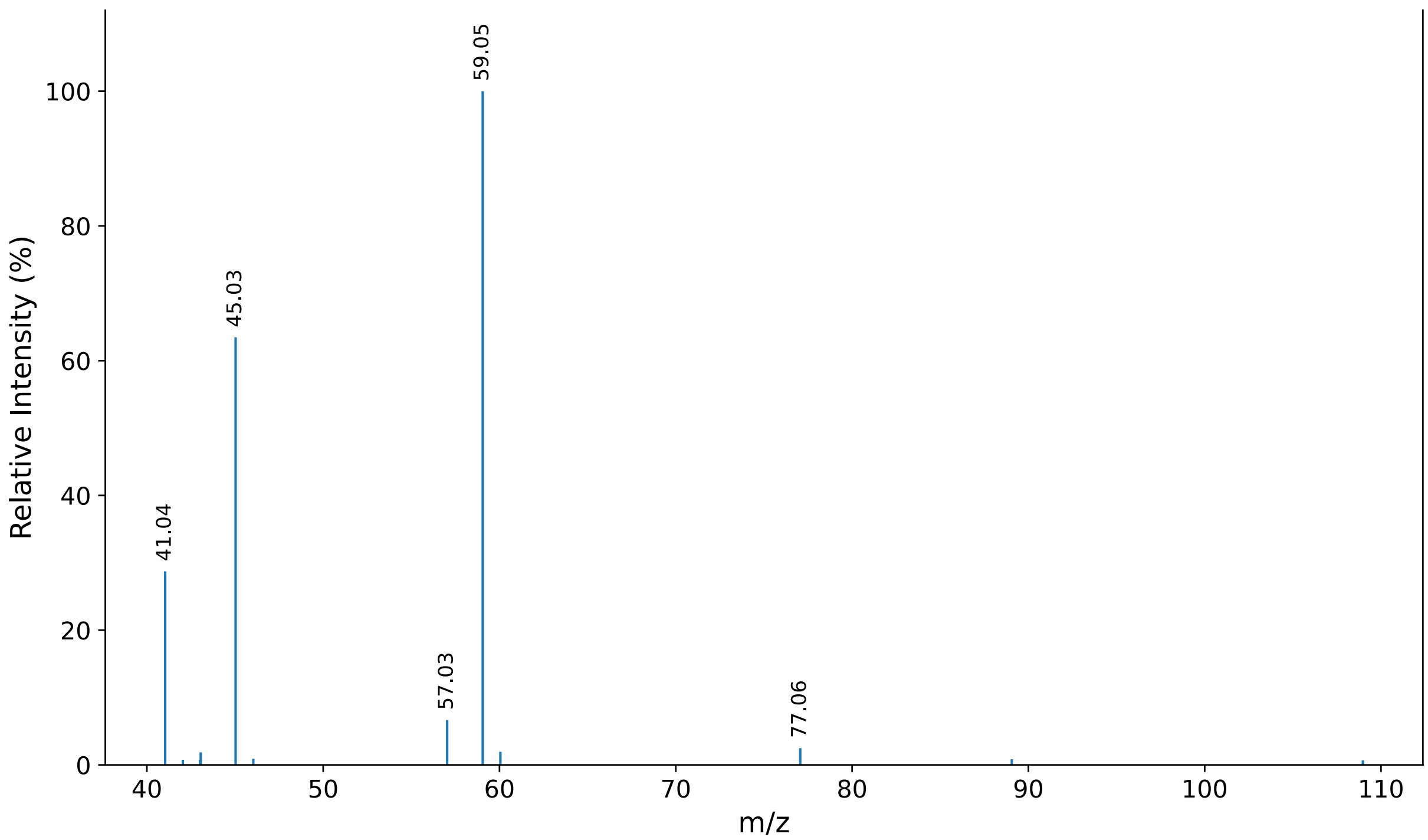

Figure S21: Energy-resolved MS/MS spectrum acquired at an absolute HCD collision voltage of 19 V. Intensities are reported relative to the base peak (100%).

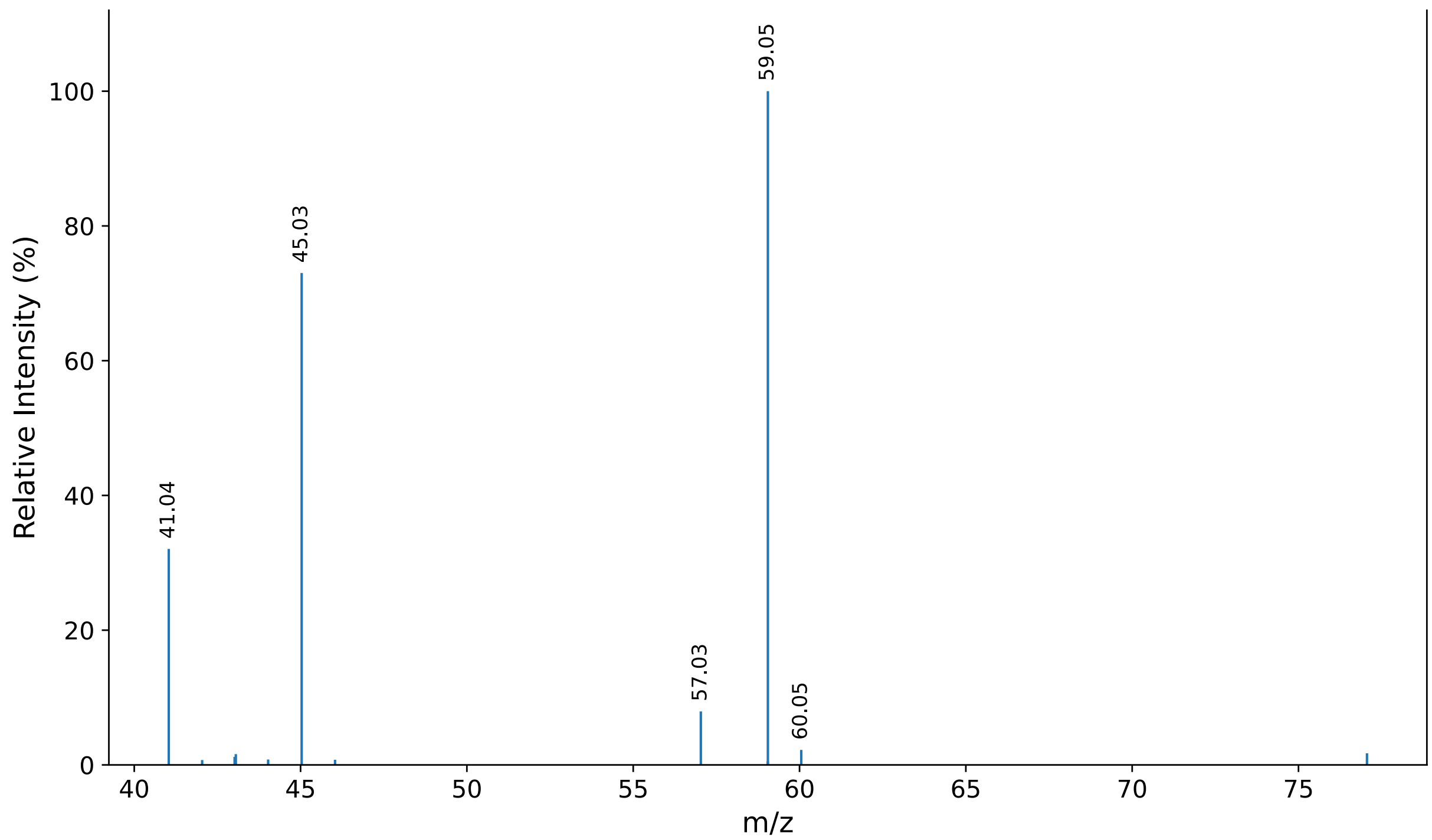

Figure S22: Energy-resolved MS/MS spectrum acquired at an absolute HCD collision voltage of 20 V. Intensities are reported relative to the base peak (100%).

S5 Full-scan MS1 and pseudo-MS3 spectra

Figure S23 shows the full-scan MS1 spectrum. Figures S24–S26 show the pseudo-MS3 experiments performed by isolating the selected ions at m/z 77, 89, and 117, respectively, followed by fragmentation at a collision energy of 4 eV. These experiments were used to evaluate the product-ion profile generated from each isolated ion. Spectral intensities are presented relative to the base peak (100%).

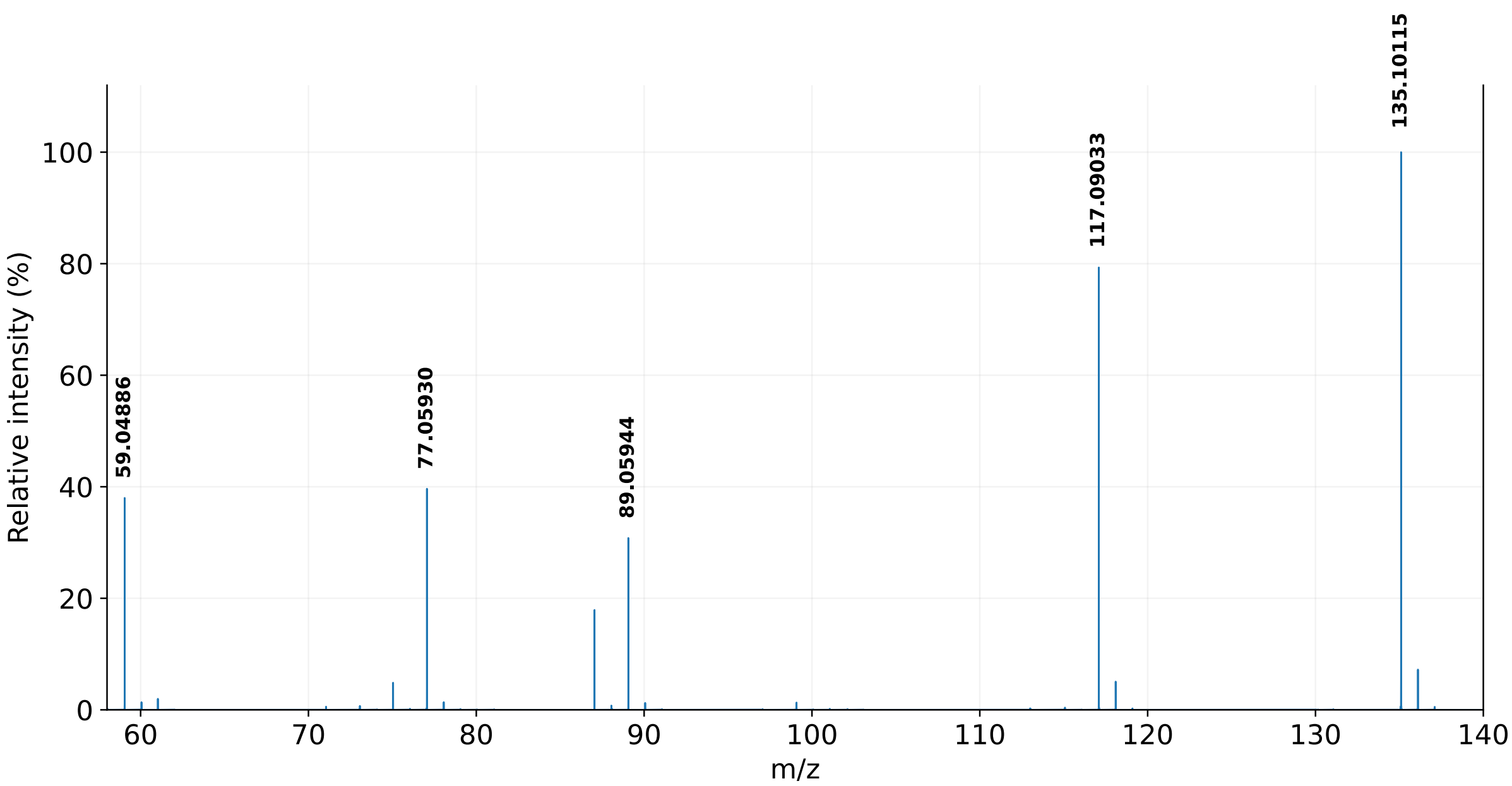

Figure S23: Full-scan MS1 spectrum. Intensities are reported relative to the base peak (100%).

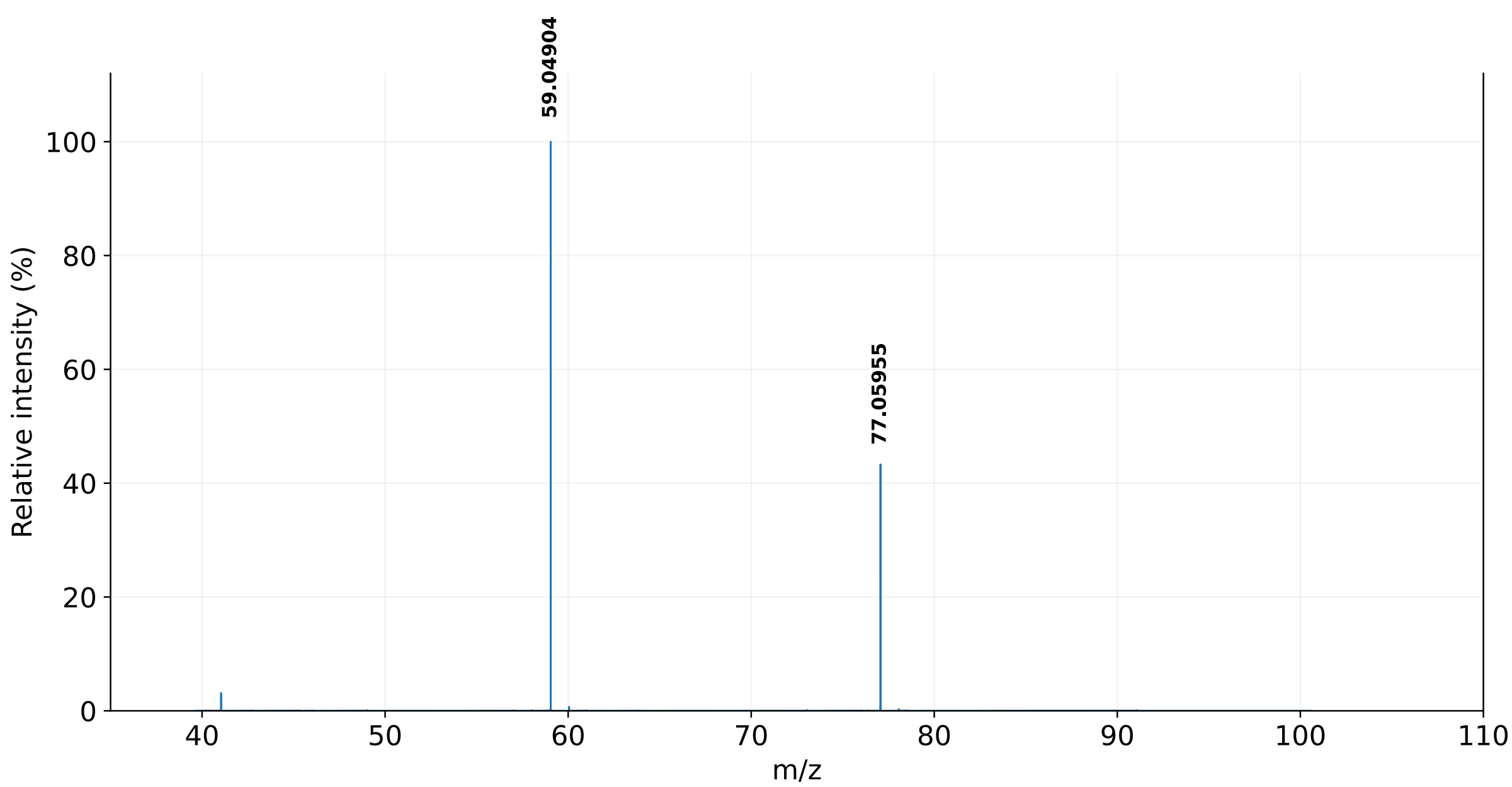

Figure S24: Pseudo-MS3 spectrum obtained by isolation of the ion at m/z 77 followed by fragmentation at a collision energy of 4 eV. The experiment was performed to evaluate the product-ion profile generated from the isolated m/z 77 ion. Intensities are reported relative to the base peak (100%).

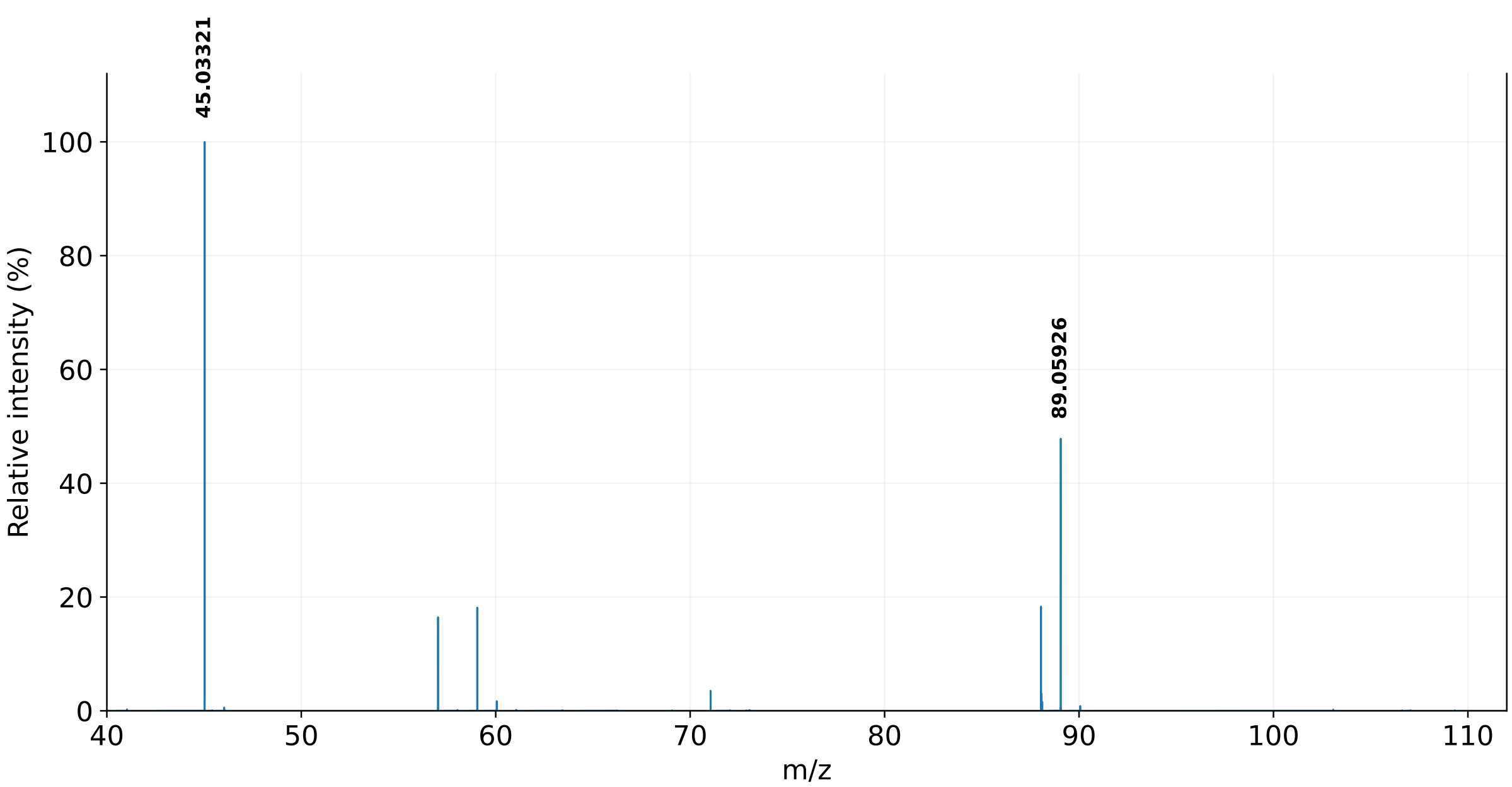

Figure S25: Pseudo-MS3 spectrum obtained by isolation of the ion at m/z 89 followed by fragmentation at a collision energy of 4 eV. The experiment was performed to evaluate the product-ion profile generated from the isolated m/z 89 ion. Intensities are reported relative to the base peak (100%).

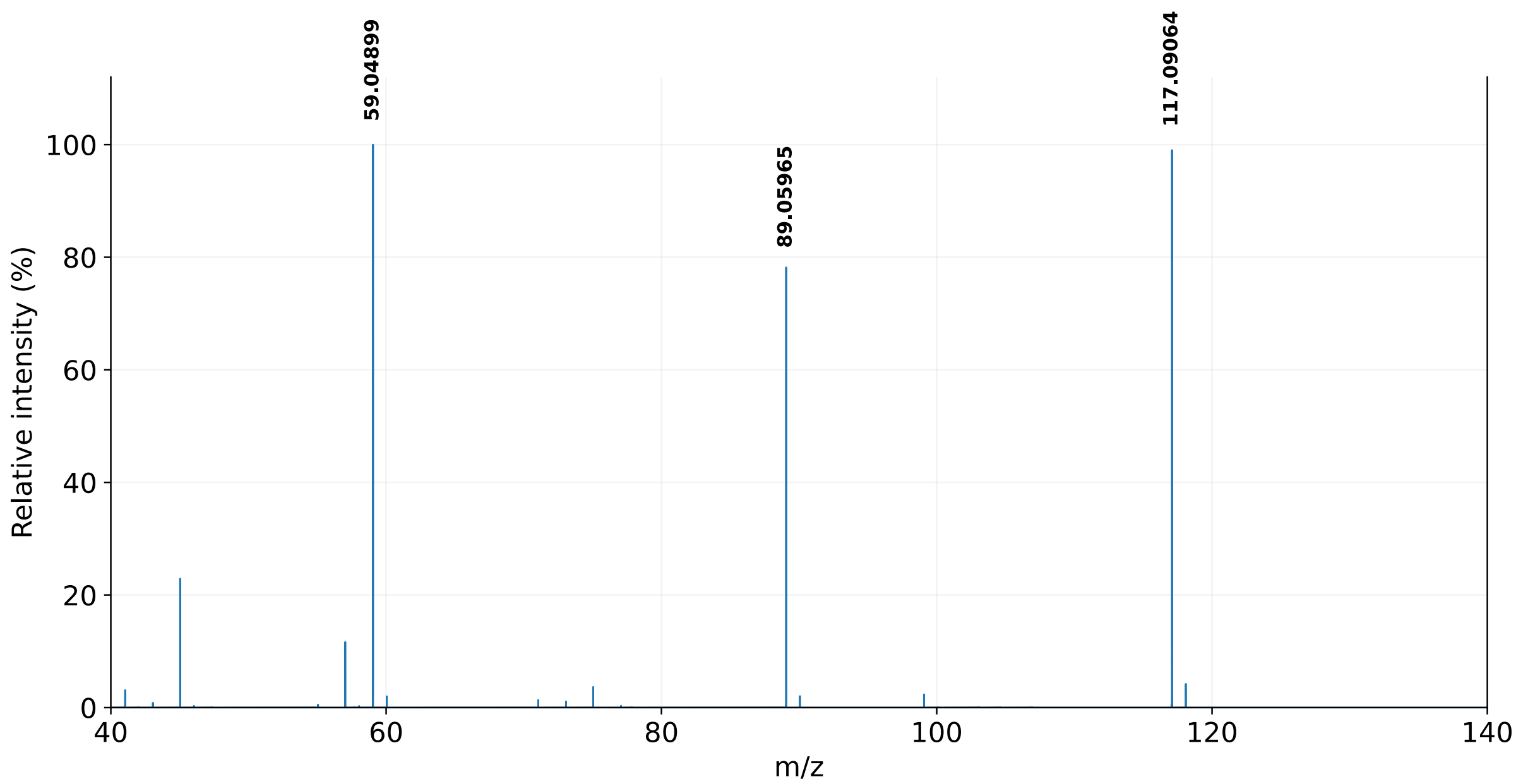

Figure S26: Pseudo-MS3 spectrum obtained by isolation of the ion at m/z 117 followed by fragmentation at a collision energy of 4 eV. The experiment was performed to evaluate the product-ion profile generated from the isolated m/z 117 ion. Intensities are reported relative to the base peak (100%).

S6 Basis-set superposition error (BSSE) analysis

The basis-set superposition error (BSSE) was evaluated using the counterpoise procedure of Boys and Bernardi.[1] For each stationary point, the molecular complex was partitioned into fragments A and B. The geometry of each fragment was kept frozen at the geometry adopted in the corresponding full complex. Fragment energies were calculated both in their own basis sets and in the full basis set of the complex by retaining the basis functions of the absent fragment as ghost functions.

The uncorrected interaction energy was calculated as

$$\Delta E_{\text{int}} = E_{AB}^{AB} - E_{A}^{A} - E_{B}^{B}. \tag{S1}$$

Here, the subscript denotes the real system present in the calculation and the superscript denotes the basis set employed.

The BSSE was obtained from

$$E_{\text{BSSE}} = \left(E_{A}^{A} - E_{A}^{AB}\right) + \left(E_{B}^{B} - E_{B}^{AB}\right), \tag{S2}$$

where E_{A}^{AB} is the energy of fragment A computed with the basis functions of fragment B retained as ghost functions, and analogously for E_{B}^{AB}.

The counterpoise-corrected interaction energy was calculated as

$$\Delta E_{\text{int}}^{\text{CP}} = E_{AB}^{AB} - E_{A}^{AB} - E_{B}^{AB}. \tag{S3}$$

S6.1 Raw electronic energies

Table S11: Electronic energies used in the Boys–Bernardi counterpoise analysis.

Quantity	PC (post-TS complex) / E_h	TS / E_h
E_{A}^{AB}	-269.292683147565	-269.294595106057
E_{A}^{A}	-269.292442654284	-269.294503071921
E_{B}^{AB}	-193.190089620510	-193.162669639271
E_{B}^{B}	-193.190089912075	-193.162654280706
E_{AB}^{AB}	-462.553851554211	-462.502195635633

E_{A}^{AB}: fragment A with fragment B represented only by ghost basis functions.
E_{B}^{AB}: fragment B with fragment A represented only by ghost basis functions.
E_{AB}^{AB}: full complex calculated in the complete basis of fragments $A + B$.

S6.2 BSSE and counterpoise-corrected interaction energies

For the post-transition-state complex (PC), the calculated BSSE was 0.631 kJ mol^{-1}, whereas the transition state showed a BSSE of 0.282 kJ mol^{-1}. The differential BSSE between the TS and PC was therefore

$$\Delta E_{\text{BSSE}}^{\text{TS}-\text{PC}} = E_{\text{BSSE}}(\text{TS}) - E_{\text{BSSE}}(\text{PC}) = -0.349 \text{ kJ mol}^{-1}. \tag{S4}$$

[1] S. F. Boys and F. Bernardi, *Molecular Physics* **1970**, *19*, 553–566. DOI: 10.1080/00268977000101561.

Table S12: BSSE contributions and interaction energies in kJ mol^{-1}.

Quantity	PC (post-TS complex)	TS
$E_A^A - E_A^{AB}$	0.6314	0.2416
$E_B^B - E_B^{AB}$	-0.0008	0.0403
E_{BSSE}	**0.6306**	**0.2820**
ΔE_{int}	-187.2480	-118.2480
$\Delta E_{\mathrm{int}}^{\mathrm{CP}}$	-186.6173	-117.9660

Positive E_{BSSE} values denote the magnitude of the artificial stabilization caused by basis-set superposition.
The counterpoise correction makes the interaction energy less negative by E_{BSSE} under the sign convention adopted here.

Thus, the BSSE contribution to the relative TS–PC energy is small (0.349 kJ mol^{-1} in magnitude).

S6.3 Reference

S. F. Boys and F. Bernardi, "The calculation of small molecular interactions by the differences of separate total energies. Some procedures with reduced errors," *Molecular Physics* **19** (1970) 553–566. DOI: 10.1080/00268977000101561.

Coordinate-record note. Cartesian coordinates are reported in XYZ format exactly as provided for the ORCA calculations. The first line gives the number of atoms, and the second line gives the ORCA job label and the electronic energy when present. Energies are reported in Hartree (E_h). ForMileS run-summary outputs supplied for molecular graphic structure generation are reported after the coordinate records.

S7 Overview of coordinate files

Table S13: Overview of Cartesian-coordinate files and associated ORCA electronic energies.

File/structure label	Atoms	Energy / E_h
135-117A_concerted_117A(RC)	24	-463.004283888775
135-117A_concerted_p1c1	24	-463.018158844484
135-117A_concerted_p2c1	24	-463.025184294047
135-117A_concerted_TS1	24	-462.958450664551
135-117A_concerted_117A	21	-386.560719601418
135-117A_stepwise_int1(RC)	24	-462.980811447554
135-117A_stepwise_p1c2	24	-463.025195306902
135-117A_stepwise_p2c1	24	-463.025184294047
135-117A_stepwise_int1	21	-386.538760308744
135-117A_stepwise_TS1	24	-462.951005655243
135-117A_stepwise_TS2	21	-386.512786421583
135-117A_stepwise_117A	21	-386.562601776783
135-117B_concerted_int1(RC)	24	-463.004283888775
135-117B_concerted_p1c1	24	-463.018158844484
135-117B_concerted_p2c1	24	-463.025184294047
135-117B_concerted_int1	21	-386.560719601418
135-117B_concerted_TS1	24	-462.958450664551
135-117B_concerted_TS2	21	-386.432311883566
135-117B_concerted_117B	21	-386.564267314034
135-117B_stepwise_int1(RC)	24	-462.980811447554
135-117B_stepwise_p1c2	24	-463.025195306902
135-117B_stepwise_p2c1	24	-463.025184294047
135-117B_stepwise_int1	21	-386.538760308744
135-117B_stepwise_int2	21	-386.562601776783
135-117B_stepwise_TS1	24	-462.951005655243
135-117B_stepwise_TS2	21	-386.512786421583
135-117B_stepwise_TS3	21	-386.432311883566
135-117B_stepwise_117B	21	-386.564267314034
135-59A_int1(RC)	24	-462.975941718788
135-59A_p2c1	24	-463.025184294047
135-59A_p2c2	24	-462.990235729145
135-59A_int1	11	-193.394998130252
135-59A_int2	11	-193.384971288740
135-59A_TS1	24	-462.950352782203
135-59A_TS2	11	-193.374979799613
135-59A_TS3	11	-193.353280120203
135-59A_neutral_loss	13	-269.531321228443
135-59A_59A	11	-193.394212184481
135-59B_int1(RC)	24	-462.975941718788
135-59B_p2c1	24	-463.025184294047
135-59B_p2c2	24	-462.990235729145
135-59B_int1	11	-193.386985009085
135-59B_TS1	24	-462.950352782203
135-59B_TS2	11	-193.337004021695
135-59B_neutral_loss	13	-269.531321228443

Table S13 continued

File/structure label	Atoms	Energy / E_h
135-59B_59B	11	-193.443855492979
135-77_77(RC)	24	-462.977929806134
135-77_TS1	24	-462.931259022403
135-77_neutral_loss	10	-193.082680792235
135-77_77	14	-269.867863166411

S8 Cartesian coordinates used in ORCA calculations

S8.1 Reaction pathway: 135-117A concerted

S8.1.1 135-117A_concerted_117A(RC)

Source file: 135-117A_concerted_117A(RC).xyz

ORCA record: Coordinates from ORCA-job opt_TS13foward E -463.004283888775

```
24
Coordinates from ORCA-job opt_TS13foward E -463.004283888775
  O         -2.10868193288225      0.24758591519282     -0.31183924173526
  C         -0.54093023531876      2.44368562242774      1.21915690097781
  H          0.10237062853745      3.62713093010235     -0.38299467306234
  H         -1.65426202246851     -0.73121865859236      0.19365906576549
  O          0.62665516011293      0.34476387778565     -0.23514691980422
  C          0.15417122829704      2.65811352031037      0.10302410373591
  H         -1.15776240991526      3.22077719903495      1.65091932394944
  H         -0.45517034035387      1.51486168677255      1.77112596364776
  C          1.05730869653162      1.66056855805979     -0.55358178438190
  H         -1.58283361118142      1.05088202179767     -0.08206250401834
  H          2.09151889934297      1.82182216058596     -0.21761983089464
  H          1.04701410241960      1.80138495964792     -1.64136851981438
  C          1.45451311338578     -0.68219986906665     -0.76713664342079
  C          0.67686686188146     -1.98788634782210     -0.78579850260131
  H          1.76670650008446     -0.43044870438846     -1.78606725171352
  H          2.35857709223642     -0.77760376664989     -0.15323868333398
  C          0.06470193998095     -2.38126848130815      0.54397504658793
  H         -0.10467493192504     -1.96411812676080     -1.54966541756770
  H          1.36433593409566     -2.78502039223035     -1.07891126431811
  O         -1.28262322727921     -1.78238938350874      0.68508837061635
  H         -0.09723350738389     -3.45553028490955      0.60399084490993
  H          0.66576267737820     -2.04741795989431      1.38970656078216
  H         -1.66374229321221     -1.98543965020945      1.54945636864500
  H         -3.05347981579712      0.41609656213000     -0.18794290239263
```

S8.1.2 135-117A_concerted_p1c1

Source file: 135-117A_concerted_p1c1.xyz

ORCA record: Coordinates from ORCA-job opt_TS13backward E -463.018158844484

```
24
Coordinates from ORCA-job opt_TS13backward E -463.018158844484
  O         -1.86378790991889      0.73015320424164      0.48920287125158
  C         -1.04459424039693      1.90835512194072      0.91861232656433
  H          0.10669112326020      3.43222456644113      0.01112665259334
  H         -1.41563646411758     -0.28555243969697      0.49759283915790
```

```
 O          0.49421517148742      0.22476131608642     -0.66905365362424
 C         -0.23063364965356      2.42456402783769     -0.24624141528116
 H         -1.77395665104612      2.64059252564871      1.25682447798808
 H         -0.43432488108669      1.58062833451308      1.75933070554564
 C          0.97267187482335      1.55301619417679     -0.55833746517961
 H         -0.87847994576701      2.51736035151483     -1.12029484382032
 H          1.71687815768464      1.61660775978123      0.24744664431849
 H          1.45415253003823      1.87683052796089     -1.48723752651395
 C          1.48642747319146     -0.76626443061398     -0.88847894313390
 C          0.80282324569905     -2.11609588238747     -0.79577617514610
 H          1.95165850507191     -0.63163289232033     -1.87090612651236
 H          2.27344578157860     -0.67844156868205     -0.12713840496238
 C          0.09046902192694     -2.34400336626444      0.52227276016654
 H          0.09395892202456     -2.24382490776170     -1.61688665762266
 H          1.55861090229563     -2.89880772705936     -0.90231725849430
 O         -1.19149524133472     -1.61943292045131      0.56363433158442
 H         -0.15600350535668     -3.39730917526135      0.64697071030696
 H          0.69074022839783     -2.01615888491734      1.37289649535699
 H         -1.82097509269155     -2.11518060803331      1.10079503596519
 H         -2.73336095852390      0.73367176887169      0.91395539491563
```

S8.1.3 135-117A_concerted_p2c1

Source file: 135-117A_concerted_p2c1.xyz

ORCA record: Coordinates from ORCA-job opt_initial E -463.025184294047

```
24
Coordinates from ORCA-job opt_initial E -463.025184294047
 O         -0.51647974011240      0.01491989013375      1.60242890130935
 C         -1.00216490208560      1.14868405610081      0.76567552927398
 H         -0.69388547581682      1.94067668822624     -1.18795534440332
 H         -0.84451622782628     -0.88261232346461      1.21440563491742
 O          1.59281060864187     -0.00005971613618      0.36732380992682
 C         -0.38691046979534      1.05808914484676     -0.62073579852803
 H         -2.08679002247303      1.08199675658907      0.76029042196097
 H         -0.69785622837815      2.03986242253094      1.31090819547592
 C          1.13923022833417      1.00631478591719     -0.57813368010888
 H         -0.78924875959223      0.18856510394277     -1.14344262843699
 H          1.55462744927826      1.95199910319302     -0.22677732651837
 H          1.55606292798421      0.79375311870283     -1.56265680752260
 C          2.16989619251365     -1.21038843644337     -0.16794257857372
 C          1.19383704499781     -2.19754848128880     -0.80326843787424
 H          2.92418460284641     -0.91647514839336     -0.90016220345171
 H          2.68625145845100     -1.66745161221599      0.67692604800228
 C          0.25081898828120     -2.95867189017868      0.11906711680076
 H          0.62329933388815     -1.73310081568469     -1.61122357586413
 H          1.82241907924816     -2.95257000435136     -1.28619311167964
 O         -0.94108629133336     -2.17485482480176      0.43348846177926
 H         -0.08708932368542     -3.86529066670732     -0.38165979168985
 H          0.74620593815235     -3.24226177943428      1.05157388804973
 H         -1.65309620532253     -2.77102310013364      0.69691324915015
 H          0.49339089916463     -0.04740998487264      1.34131119813647
```

S8.1.4 135-117A_concerted_TS1

Source file: 135-117A_concerted_TS1.xyz

ORCA record: Coordinates from ORCA-job opt_TS13 E -462.958450664551

```
24
```

```
Coordinates from ORCA-job opt_TS13 E -462.958450664551
  O         -2.30984880632553      0.65122544548731      0.03905123836773
  C         -0.71419690362599      2.15908540048895      1.09345483037543
  H         -0.04131255136562      3.56215496204953     -0.36558082069510
  H         -1.87456449206920     -0.22070177905590      0.18638189364751
  O          0.70415665094526      0.32576535812131     -0.26629134487967
  C         -0.02034794127003      2.51786391535885     -0.06359611836023
  H         -1.46962113369853      2.81298996395667      1.51362893673563
  H         -0.54333019328689      1.20100545981293      1.56648876539519
  C          1.11086471108595      1.63266805171452     -0.56041440904786
  H         -1.00656276523956      2.04773492833055     -0.50753361921181
  H          2.04488415344774      1.91019250578197     -0.05221104772933
  H          1.25559518762049      1.78676562186760     -1.63566113965166
  C          1.54689573956949     -0.73776458502306     -0.71289387804203
  C          0.71986261300586     -2.00924957271901     -0.77087244820055
  H          1.94134457045623     -0.50461938642043     -1.70757856630628
  H          2.39439496425542     -0.84432464349481     -0.02563103058530
  C          0.11155251042619     -2.46334797383594      0.54463750801322
  H         -0.07166044863906     -1.90109756967038     -1.51697559041350
  H          1.37607705565794     -2.81030116672138     -1.12259438286650
  O         -1.00782599537733     -1.62341447326833      0.91231042565082
  H         -0.24074923955743     -3.49166912482897      0.43849324554546
  H          0.85773631794708     -2.43654005074772      1.34532001493388
  H         -1.49981683781890     -2.06965511248938      1.61090734343418
  H         -3.26352716614359      0.50523382530517      0.05716019389074
```

S8.1.5 135-117A_concerted_117A

Source file: 135-117A_concerted_117A.xyz

ORCA record: Coordinates from ORCA-job opt_end13_NoH2O E -386.560719601418

```
21
Coordinates from ORCA-job opt_end13_NoH2O E -386.560719601418
  H          0.30424562356188     -2.89460177398402      1.02439452582128
  C         -0.33538561459208      2.58284802011043      1.33964518320853
  H         -0.75285800510945      3.11451988841788     -0.64486970733898
  H          0.02098871072256     -0.14073747855977      0.58787534370111
  O          0.90033151118749      0.29986630787515      0.23659111590887
  C         -0.22522611765802      2.44475744742369      0.02603268361596
  H         -0.95809237333758      3.36047485669323      1.75992410003481
  H          0.18641183620662      1.94673115551268      2.04389925106508
  C          0.59862580960567      1.42257415044423     -0.68139571215101
  H         -1.52535943197181     -1.46473791052417      1.41332888537315
  H          1.57359467706773      1.80522964822028     -0.98616028485762
  H          0.08111478154880      1.01609699556922     -1.55208693318385
  C          1.69556352658471     -0.84098813226491     -0.31321405028956
  C          0.76169712715447     -1.90672933306982     -0.85641227358281
  H          2.35499153361424     -0.42007742304825     -1.06750769929199
  H          2.28464619205236     -1.18452334071795      0.53516633293854
  C         -0.21654477721882     -2.41658498015364      0.19131092994342
  H          0.21493939336436     -1.53007711585369     -1.72396811080291
  H          1.37382841892797     -2.74061303987021     -1.20840292594123
  O         -0.93867186971346     -1.24949696744529      0.67766220952714
  H         -0.92473708569766     -3.12136408537509     -0.24323980509793
```

S8.2 Reaction pathway: 135-117A stepwise

S8.2.1 135-117A_stepwise_int1(RC)

Source file: 135-117A_stepwise_int1(RC) .xyz

ORCA record: Coordinates from ORCA-job opt__TS1foward E -462.980811447554

```
24
Coordinates from ORCA-job opt_TS1foward E -462.980811447554
  O         -0.57454884166269    -0.02934523534677     2.32839386596982
  C         -1.03281296700550     2.49246328862479     0.25450817554918
  H         -1.96319749511804     2.81220582905199    -0.22321472374119
  H         -1.17278518795106    -0.64244777193367     1.87674318134859
  O          0.97267339355067     1.03135653846223    -0.18105844190246
  C         -0.47845395329274     1.36775935764157    -0.54125973074885
  H         -1.26276676798018     2.14943655290408     1.26207951890700
  H         -0.35254339080457     3.34167538518458     0.29242574809889
  C          0.59568696704774     1.53075394104190    -1.52385352613403
  H         -1.05889089340816     0.45531755542346    -0.60392046204404
  H          1.03338351282491     2.50580478244814    -1.69733391591692
  H          0.71816271109305     0.80461516763107    -2.31603603596627
  C          1.54555947042134    -0.34153099890485     0.06263172493492
  C          0.96795110060382    -1.50412573263852    -0.72798451432678
  H          2.59283951838645    -0.17193485417650    -0.17637302253626
  H          1.40632590569747    -0.44465460807832     1.13567700100060
  C         -0.13689963358875    -2.31551660289080    -0.06606076712639
  H          0.66741485495623    -1.20610300875177    -1.73542649238341
  H          1.80793505139471    -2.18931302343622    -0.87801457942182
  O         -1.34157221744458    -1.54136626063791     0.05358457009433
  H         -0.32485900399335    -3.20326577076571    -0.67247813915744
  H          0.18597396645374    -2.64409647292087     0.92631869887006
  H         -2.10604981126395    -2.12460181156701    -0.01301986925071
  H         -0.75755245512553    -0.09730934230047     3.27145938864438
```

S8.2.2 `135-117A_stepwise_p1c2`

Source file: `135-117A_stepwise_p1c2 .xyz`

ORCA record: Coordinates from ORCA-job opt__TS1backward E -463.025195306902

```
24
Coordinates from ORCA-job opt_TS1backward E -463.025195306902
  O         -0.85874639719984     0.68230047675860     1.60989875760894
  C         -1.36459344490779     1.79907223133408     0.76248229507636
  H         -1.09639430355335     2.55617626807399    -1.21101956379659
  H         -1.19438570124527    -0.22200958312083     1.24639572509107
  O          1.22463898483291     0.64896730720705     0.33495351801291
  C         -0.77630115349956     1.68469975608444    -0.63398570564673
  H         -2.44892040697503     1.72948995812755     0.77948596032776
  H         -1.05215706965872     2.70102971348169     1.28485534335231
  C          0.75055240952203     1.63720935822910    -0.61989459745713
  H         -1.18620535955915     0.80483638074624    -1.13290453335499
  H          1.17024622466668     2.58985408627045    -0.29338578354057
  H          1.14934523109822     1.40791377017552    -1.60812478810236
  C          1.79318893555935    -0.57066925170395    -0.18878446482077
  C          0.80726679509809    -1.56876464497065    -0.79108085020530
  H          2.53539751142797    -0.28909696734649    -0.93796960299433
  H          2.32311343267489    -1.01306146447768     0.65546027084188
  C         -0.11842800975445    -2.31620590628194     0.15938821864994
  H          0.22286588175915    -1.11796085839468    -1.59681244212819
  H          1.42855967473041    -2.33089627075901    -1.27232097175834
  O         -1.30400610569517    -1.52784358603300     0.48551199808277
  H         -0.46561686141861    -3.22961226235745    -0.32233113089814
  H          0.39457238649227    -2.58713754365261     1.08614659799062
  H         -2.01113526220094    -2.12031238780122     0.76955235589120
  H          0.14616877485556     0.61624451469301     1.32669574023822
```

S8.2.3 135-117A_stepwise_p2c1

Source file: 135-117A_stepwise_p2c1.xyz

ORCA record: Coordinates from ORCA-job opt_initial E -463.025184294047

```
24
Coordinates from ORCA-job opt_initial E -463.025184294047
  O         -0.51647974011240      0.01491989013375      1.60242890130935
  C         -1.00216490208560      1.14868405610081      0.76567552927398
  H         -0.69388547581682      1.94067668822624     -1.18795534440332
  H         -0.84451622782628     -0.88261232346461      1.21440563491742
  O          1.59281060864187     -0.00005971613618      0.36732380992682
  C         -0.38691046979534      1.05808914484676     -0.62073579852803
  H         -2.08679002247303      1.08199675658907      0.76029042196097
  H         -0.69785622837815      2.03986242253094      1.31090819547592
  C          1.13923022833417      1.00631478591719     -0.57813368010888
  H         -0.78924875959223      0.18856510394277     -1.14344262843699
  H          1.55462744927826      1.95199910319302     -0.22677732651837
  H          1.55606292798421      0.79375311870283     -1.56265680752260
  C          2.16989619251365     -1.21038843644337     -0.16794257857372
  C          1.19383704499781     -2.19754848128880     -0.80326843787424
  H          2.92418460284641     -0.91647514839336     -0.90016220345171
  H          2.68625145845100     -1.66745161221599      0.67692604800228
  C          0.25081898828120     -2.95867189017868      0.11906711680076
  H          0.62329933388815     -1.73310081568469     -1.61122357586413
  H          1.82241907924816     -2.95257000435136     -1.28619311167964
  O         -0.94108629133336     -2.17485482480176      0.43348846177926
  H         -0.08708932368542     -3.86529066670732     -0.38165979168985
  H          0.74620593815235     -3.24226177943428      1.05157388804973
  H         -1.65309620532253     -2.77102310013364      0.69691324915015
  H          0.49339089916463     -0.04740998487264      1.34131119813647
```

S8.2.4 135-117A_stepwise_int1

Source file: 135-117A_stepwise_int1 .xyz

ORCA record: Coordinates from ORCA-job opt_TS2foward_step2 E -386.538760308744

```
21
Coordinates from ORCA-job opt_TS2foward_step2 E -386.538760308744
  H          0.49128105356310     -2.24712313899819      1.70430558087065
  C         -1.54294597418128      1.84614049011768     -0.10501702842308
  H         -2.00174860315673      2.60669724084318     -0.74317307689694
  H         -1.72507195618255     -1.41568550890250      1.98533270679685
  O          0.67668947395022      0.67306662722958      0.20377250112843
  C         -0.25366932314547      1.45060442607644     -0.72692696365261
  H         -2.21251479500529      0.99028546608462     -0.04388967063980
  H         -1.39744078085694      2.26492405509019      0.88956185627507
  C          1.04465493680241      2.00792325350186     -0.34214007933183
  H         -0.29334227580919      0.96154660196543     -1.69402048170541
  H          1.10567150732286      2.73847211951396      0.45405486736891
  H          1.87619772729062      1.97510884133721     -1.03465016187084
  C          1.38558650074722     -0.55094770166893     -0.29513358637810
  C          0.43619923089723     -1.71954511364276     -0.39217633420754
  H          1.82804853055054     -0.29758351735757     -1.25896578865960
  H          2.16658813927038     -0.69209573855384      0.45039567200943
  C         -0.24766469655544     -2.12878266373323      0.90220611282184
  H         -0.31937272487537     -1.53751795486776     -1.16091744223230
  H          1.04556753694228     -2.55479848119503     -0.75252314892611
  O         -1.20185092832503     -1.12344626525195      1.23057545652490
  H         -0.73563790519153     -3.09557423045459      0.74867439197989
```

S8.2.5 135-117A_stepwise_TS1

Source file: 135-117A_stepwise_TS1 .xyz

ORCA record: Coordinates from ORCA-job opt__TS1__step1 E -462.951005655243

```
24
Coordinates from ORCA-job opt_TS1_step1 E -462.951005655243
  O        -1.30733051012109      0.73471742174489      2.32954712673441
  C        -1.39818836899444      2.18262633475856      0.31195180439889
  H        -1.34412858880945      2.57331736169808     -0.92232707724529
  H        -0.92698962196341     -0.06481476216501      1.92781640308123
  O         1.26770685487942      0.50851539192431      0.15438885316793
  C        -0.69314384763197      1.40437572575904     -0.58888678536653
  H        -2.44596255714225      1.99761989814029      0.50566513434669
  H        -0.89429202694891      2.95149913760875      0.88390197869626
  C         0.80359143304150      1.49115784202187     -0.74202638744890
  H        -1.22821895537152      0.61304192952172     -1.10275838276468
  H         1.17515524149079      2.47116819898939     -0.43126619012214
  H         1.11192143881234      1.30335015343298     -1.77307858273551
  C         1.88645367345511     -0.65709837009780     -0.42088703772562
  C         0.91455158963992     -1.68068072710669     -0.98810800061184
  H         2.59752144124515     -0.33972306575856     -1.18952430675723
  H         2.45291038244299     -1.08756132769837      0.40548772886971
  C        -0.02531541362280     -2.32733385415041      0.01482663411504
  H         0.33498016387157     -1.25635306226220     -1.81485531564650
  H         1.52167506694555     -2.47612579282284     -1.43159848461708
  O        -1.02182978794595     -1.37790234631130      0.45158025786602
  H        -0.52201427841787     -3.17854047253947     -0.45517594536885
  H         0.53351698402147     -2.69538766805797      0.88113039771874
  H        -1.79671145520237     -1.86591627657413      0.75429038385705
  H        -0.99585885767373      0.77604832994486      3.23990579355819
```

S8.2.6 135-117A_stepwise_TS2

Source file: 135-117A_stepwise_TS2 .xyz

ORCA record: Coordinates from ORCA-job opt__TS2__step2 E -386.512786421583

```
21
Coordinates from ORCA-job opt_TS2_step2 E -386.512786421583
  H         0.03960565781562     -2.02278409596704      1.67464870735053
  C        -1.39766116691943      1.75665734759498      0.37160493147772
  H        -2.42620127063784      2.09585743058905      0.24406104788078
  H        -2.21709439617624     -1.44445180041011      1.14989405578784
  O         1.02443985847806      0.81561437848122      0.34761997006118
  C        -0.65099801829271      1.70834870155871     -0.81447613170314
  H        -1.49654013444720      0.65654040486669      0.56965207897454
  H        -0.87624466896325      2.12617613510705      1.25187281504542
  C         0.84443963349620      1.65262714274598     -0.79457968541330
  H        -1.18169398798839      1.59657811084111     -1.76027189355719
  H         1.26954820287947      2.65203473994549     -0.62779669360360
  H         1.25979161579332      1.23756227493617     -1.71473873939452
  C         1.64838546919020     -0.46024937978870      0.11585373124581
  C         0.69229817053202     -1.55563482057563     -0.33821221611876
  H         2.46372015349464     -0.34317515112928     -0.60241580966918
  H         2.08899120451580     -0.71832335412357      1.08002539390029
  C        -0.37209761915193     -1.98025353332383      0.66216116406726
  H         0.21460259640752     -1.29489719843497     -1.28965104686807
  H         1.30968155525153     -2.43369509488058     -0.54988481118642
  O        -1.49641842523755     -1.07097466510593      0.62734588250446
  H        -0.74055443003982     -2.97355757292683      0.39728724921834
```

S8.2.7 135-117A_stepwise_117A

Source file: 135-117A_stepwise_117A.xyz

ORCA record: Coordinates from ORCA-job opt__TS2backward__step2 E -386.562601776783

```
21
Coordinates from ORCA-job opt_TS2backward_step2 E -386.562601776783
  H         0.45828010289914     -2.69404913503855      1.40102977412164
  C        -1.69617004190042      2.29669667590248     -0.68579061945512
  H        -2.51820299773039      2.28714570072905     -1.38940406146584
  H        -1.06915552192371     -1.24435630639667      2.41266861988569
  O         0.94248510590809      0.45549537326961      0.57595428057578
  C        -0.52602459032757      1.74113730348017     -0.97863283288234
  H         0.16051519340923      0.05930487284134      1.13158843497726
  H        -1.86968476710966      2.80037073377854      0.25862536377284
  C         0.62957366487463      1.79169333993643     -0.04886011316845
  H        -0.36978524517448      1.27263188815786     -1.94463957257174
  H         0.45298676918440      2.43091257164607      0.81272094059961
  H         1.56751182075346      2.07095325924908     -0.52486156371938
  C         1.41135817607742     -0.66303157786852     -0.29537847660827
  C         0.29100293099487     -1.66569997718671     -0.50695574057170
  H         1.75761099292725     -0.21146416145783     -1.22148155776280
  H         2.25912267778446     -1.08480712481027      0.24176513961094
  C        -0.29543455259670     -2.18223831940348      0.79759781824811
  H        -0.50140561825432     -1.22721685665844     -1.11585740840851
  H         0.70138058853869     -2.50863499130544     -1.06849396735528
  O        -0.78424482840044     -1.01550869446563      1.51913057631930
  H        -1.12694453372281     -2.86100338188896      0.60992958263299
```

S8.3 Reaction pathway: 135-117B concerted

S8.3.1 135-117B_concerted_int1(RC)

Source file: 135-117B_concerted_int1(RC) .xyz

ORCA record: Coordinates from ORCA-job opt__TS13foward E -463.004283888775

```
24
Coordinates from ORCA-job opt_TS13foward E -463.004283888775
  O        -2.10868193288225      0.24758591519282     -0.31183924173526
  C        -0.54093023531876      2.44368562242774      1.21915690097781
  H         0.10237062853745      3.62713093010235     -0.38299467306234
  H        -1.65426202246851     -0.73121865859236      0.19365906576549
  O         0.62665516011293      0.34476387778565     -0.23514691980422
  C         0.15417122829704      2.65811352031037      0.10302410373591
  H        -1.15776240991526      3.22077719903495      1.65091932394944
  H        -0.45517034035387      1.51486168677255      1.77112596364776
  C         1.05730869653162      1.66056855805979     -0.55358178438190
  H        -1.58283361118142      1.05088202179767     -0.08206250401834
  H         2.09151889934297      1.82182216058596     -0.21761983089464
  H         1.04701410241960      1.80138495964792     -1.64136851981438
  C         1.45451311338578     -0.68219986906665     -0.76713664342079
  C         0.67686686188146     -1.98788634782210     -0.78579850260131
  H         1.76670650008446     -0.43044870438846     -1.78606725171352
  H         2.35857709223642     -0.77760376664989     -0.15323868333398
  C         0.06470193998095     -2.38126848130815      0.54397504658793
  H        -0.10467493192504     -1.96411812676080     -1.54966541756770
  H         1.36433593409566     -2.78502039223035     -1.07891126431811
  O        -1.28262322727921     -1.78238938350874      0.68508837061635
  H        -0.09723350738389     -3.45553028490955      0.60399084490993
  H         0.66576267737820     -2.04741795989431      1.38970656078216
  H        -1.66374229321221     -1.98543965020945      1.54945636864500
```

```
  H         -3.05347981579712      0.41609656213000     -0.18794290239263
```

S8.3.2 135-117B_concerted_p1c1

Source file: 135-117B_concerted_p1c1 .xyz

ORCA record: Coordinates from ORCA-job opt__TS13backward E -463.018158844484

```
24
Coordinates from ORCA-job opt_TS13backward E -463.018158844484
  O         -1.86378790991889      0.73015320424164      0.48920287125158
  C         -1.04459424039693      1.90835512194072      0.91861232656433
  H          0.10669112326020      3.43222456644113      0.01112665259334
  H         -1.41563646411758     -0.28555243969697      0.49759283915790
  O          0.49421517148742      0.22476131608642     -0.66905365362424
  C         -0.23063364965356      2.42456402783769     -0.24624141528116
  H         -1.77395665104612      2.64059252564871      1.25682447798808
  H         -0.43432488108669      1.58062833451308      1.75933070554564
  C          0.97267187482335      1.55301619417679     -0.55833746517961
  H         -0.87847994576701      2.51736035151483     -1.12029484382032
  H          1.71687815768464      1.61660775978123      0.24744664431849
  H          1.45415253003823      1.87683052796089     -1.48723752651395
  C          1.48642747319146     -0.76626443061398     -0.88847894313390
  C          0.80282324569905     -2.11609588238747     -0.79577617514610
  H          1.95165850507191     -0.63163289232033     -1.87090612651236
  H          2.27344578157860     -0.67844156868205     -0.12713840496238
  C          0.09046902192694     -2.34400336626444      0.52227276016654
  H          0.09395892202456     -2.24382490776170     -1.61688665762266
  H          1.55861090229563     -2.89880772705936     -0.90231725849430
  O         -1.19149524133472     -1.61943292045131      0.56363433158442
  H         -0.15600350535668     -3.39730917526135      0.64697071030696
  H          0.69074022839783     -2.01615888491734      1.37289649535699
  H         -1.82097509269155     -2.11518060803331      1.10079503596519
  H         -2.73336095852390      0.73367176887169      0.91395539491563
```

S8.3.3 135-117B_concerted_p2c1

Source file: 135-117B_concerted_p2c1.xyz

ORCA record: Coordinates from ORCA-job opt__initial E -463.025184294047

```
24
Coordinates from ORCA-job opt_initial E -463.025184294047
  O         -0.51647974011240      0.01491989013375      1.60242890130935
  C         -1.00216490208560      1.14868405610081      0.76567552927398
  H         -0.69388547581682      1.94067668822624     -1.18795534440332
  H         -0.84451622782628     -0.88261232346461      1.21440563491742
  O          1.59281060864187     -0.00005971613618      0.36732380992682
  C         -0.38691046979534      1.05808914484676     -0.62073579852803
  H         -2.08679002247303      1.08199675658907      0.76029042196097
  H         -0.69785622837815      2.03986242253094      1.31090819547592
  C          1.13923022833417      1.00631478591719     -0.57813368010888
  H         -0.78924875959223      0.18856510394277     -1.14344262843699
  H          1.55462744927826      1.95199910319302     -0.22677732651837
  H          1.55606292798421      0.79375311870283     -1.56265680752260
  C          2.16989619251365     -1.21038843644337     -0.16794257857372
  C          1.19383704499781     -2.19754848128880     -0.80326843787424
  H          2.92418460284641     -0.91647514839336     -0.90016220345171
  H          2.68625145845100     -1.66745161221599      0.67692604800228
  C          0.25081898828120     -2.95867189017868      0.11906711680076
  H          0.62329933388815     -1.73310081568469     -1.61122357586413
```

```
  H          1.82241907924816     -2.95257000435136     -1.28619311167964
  O         -0.94108629133336     -2.17485482480176      0.43348846177926
  H         -0.08708932368542     -3.86529066670732     -0.38165979168985
  H          0.74620593815235     -3.24226177943428      1.05157388804973
  H         -1.65309620532253     -2.77102310013364      0.69691324915015
  H          0.49339089916463     -0.04740998487264      1.34131119813647
```

S8.3.4 135-117B_concerted_int1

Source file: 135-117B_concerted_int1.xyz

ORCA record: Coordinates from ORCA-job opt_end13_NoH2O E -386.560719601418

```
21
Coordinates from ORCA-job opt_end13_NoH2O E -386.560719601418
  H          0.30424562356188     -2.89460177398402      1.02439452582128
  C         -0.33538561459208      2.58284802011043      1.33964518320853
  H         -0.75285800510945      3.11451988841788     -0.64486970733898
  H          0.02098871072256     -0.14073747855977      0.58787534370111
  O          0.90033151118749      0.29986630787515      0.23659111590887
  C         -0.22522611765802      2.44475744742369      0.02603268361596
  H         -0.95809237333758      3.36047485669323      1.75992410003481
  H          0.18641183620662      1.94673115551268      2.04389925106508
  C          0.59862580960567      1.42257415044423     -0.68139571215101
  H         -1.52535943197181     -1.46473791052417      1.41332888537315
  H          1.57359467706773      1.80522964822028     -0.98616028485762
  H          0.08111478154880      1.01609699556922     -1.55208693318385
  C          1.69556352658471     -0.84098813226491     -0.31321405028956
  C          0.76169712715447     -1.90672933306982     -0.85641227358281
  H          2.35499153361424     -0.42007742304825     -1.06750769929199
  H          2.28464619205236     -1.18452334071795      0.53516633293854
  C         -0.21654477721882     -2.41658498015364      0.19131092994342
  H          0.21493939336436     -1.53007711585369     -1.72396811080291
  H          1.37382841892797     -2.74061303987021     -1.20840292594123
  O         -0.93867186971346     -1.24949696744529      0.67766220952714
  H         -0.92473708569766     -3.12136408537509     -0.24323980509793
```

S8.3.5 135-117B_concerted_TS1

Source file: 135-117B_concerted_TS1 .xyz

ORCA record: Coordinates from ORCA-job opt_TS13 E -462.958450664551

```
24
Coordinates from ORCA-job opt_TS13 E -462.958450664551
  O         -2.30984880632553      0.65122544548731      0.03905123836773
  C         -0.71419690362599      2.15908540048895      1.09345483037543
  H         -0.04131255136562      3.56215496204953     -0.36558082069510
  H         -1.87456449206920     -0.22070177905590      0.18638189364751
  O          0.70415665094526      0.32576535812131     -0.26629134487967
  C         -0.02034794127003      2.51786391535885     -0.06359611836023
  H         -1.46962113369853      2.81298996395667      1.51362893673563
  H         -0.54333019328689      1.20100545981293      1.56648876539519
  C          1.11086471108595      1.63266805171452     -0.56041440904786
  H         -1.00656276523956      2.04773492833055     -0.50753361921181
  H          2.04488415344774      1.91019250578197     -0.05221104772933
  H          1.25559518762049      1.78676562186760     -1.63566113965166
  C          1.54689573956949     -0.73776458502306     -0.71289387804203
  C          0.71986261300586     -2.00924957271901     -0.77087244820055
  H          1.94134457045623     -0.50461938642043     -1.70757856630628
  H          2.39439496425542     -0.84432464349481     -0.02563103058530
```

```
  C          0.11155251042619     -2.46334797383594      0.54463750801322
  H         -0.07166044863906     -1.90109756967038     -1.51697559041350
  H          1.37607705565794     -2.81030116672138     -1.12259438286650
  O         -1.00782599537733     -1.62341447326833      0.91231042565082
  H         -0.24074923955743     -3.49166912482897      0.43849324554546
  H          0.85773631794708     -2.43654005074772      1.34532001493388
  H         -1.49981683781890     -2.06965511248938      1.61090734343418
  H         -3.26352716614359      0.50523382530517      0.05716019389074
```

S8.3.6 135-117B_concerted_TS2

Source file: 135-117B_concerted_TS2.xyz

ORCA record: Coordinates from ORCA-job opt_TS1_step3 E -386.432311883566

```
21
Coordinates from ORCA-job opt_TS1_step3 E -386.432311883566
  H          0.02773319419939     -2.32237627848729      2.01776161589863
  C         -1.23619968880866      2.83510996601560     -0.08738222857160
  H         -1.89952947941368      3.66903427069716     -0.25518874485563
  H         -1.94458721212705     -1.12874017257828      1.57090772412345
  O          0.44362447579543      0.40111915005782      0.09441639562953
  C         -0.53024490463692      2.28434012072656     -1.15253031718285
  H         -0.44569056628705     -0.03727793926517      0.47873574007929
  H         -1.09303112072251      2.50374661305145      0.93175278379865
  C          0.20564389092824      0.97054681910173     -1.21023997185773
  H         -0.75692070264762      2.64471070733644     -2.15261959619451
  H          0.55648792588905      2.59439611833663     -0.92609384288608
  H          1.07616631808957      0.89582149709658     -1.84939070629054
  C          1.45023186131664     -0.70350668272487      0.19428593502593
  C          0.75169514666311     -2.03407263214477     -0.01206110011421
  H          2.20918007037969     -0.49224779713704     -0.55353704391253
  H          1.87194736532628     -0.58725206983491      1.19103325864448
  C         -0.36104309273076     -2.26879080760610      0.99827660575212
  H          0.35452641490944     -2.09105284130323     -1.02782557338288
  H          1.49604316739611     -2.82748906511662      0.08901525745926
  O         -1.26195720712759     -1.12648410916337      0.88829297590044
  H         -0.91407585639107     -3.17953486705829      0.77239083293679
```

S8.3.7 135-117B_concerted_117B

Source file: 135-117B_concerted_117B.xyz

ORCA record: Coordinates from ORCA-job opt_TS1foward_step3 E -386.564267314034

```
21
Coordinates from ORCA-job opt_TS1foward_step3 E -386.564267314034
  H          0.25247680316486     -2.59214932375052      1.83251940808497
  C         -0.43146442035257      3.20083433719908     -0.06258879213389
  H         -1.32221528914920      3.82942173399671      0.00930438959403
  H         -1.94693350644376     -2.32296602387260      1.05986558759701
  O         -0.27478642922516      0.26756789675523      0.17062344500586
  C         -0.59901370905793      2.21669578152373     -1.16718719155673
  H         -0.92435969129145     -0.53312013152388      0.35426783015823
  H         -0.25818128555748      2.72958949773972      0.90254613154311
  C         -0.53379195580589      0.90102848657607     -1.10101948558508
  H         -0.80226751436701      2.60954037012262     -2.15877134277314
  H          0.40278853792878      3.87083506849790     -0.28514478496876
  H         -0.66298938218943      0.19699590143902     -1.90881275766984
  C          1.09535720383730     -0.32645700098918      0.38031957126546
  C          1.07937934145004     -1.79109841188048     -0.01077417562828
```

```
  H          1.77025427322870      0.27853513480972     -0.21778374999155
  H          1.28749440579503     -0.16804170466563      1.43996785000236
  C          0.04167288929967     -2.59261712765960      0.76061753033322
  H          0.91119248805792     -1.89555472638804     -1.08504688143397
  H          2.07049387553835     -2.20296511606265      0.19520967156640
  O         -1.24141676141818     -1.94229214037447      0.52179273401370
  H         -0.00813982489866     -3.62031819406578      0.40261586265625
```

S8.4 Reaction pathway: 135-117B stepwise

S8.4.1 135-117B_stepwise_int1(RC)

Source file: 135-117B_stepwise_int1(RC).xyz

ORCA record: Coordinates from ORCA-job opt__TS1foward E -462.980811447554

```
24
Coordinates from ORCA-job opt_TS1foward E -462.980811447554
  O         -0.57454884166269     -0.02934523534677      2.32839386596982
  C         -1.03281296700550      2.49246328862479      0.25450817554918
  H         -1.96319749511804      2.81220582905199     -0.22321472374119
  H         -1.17278518795106     -0.64244777193367      1.87674318134859
  O          0.97267339355067      1.03135653846223     -0.18105844190246
  C         -0.47845395329274      1.36775935764157     -0.54125973074885
  H         -1.26276676798018      2.14943655290408      1.26207951890700
  H         -0.35254339080457      3.34167538518458      0.29242574809889
  C          0.59568696704774      1.53075394104190     -1.52385352613403
  H         -1.05889089340816      0.45531755542346     -0.60392046204404
  H          1.03338351282491      2.50580478244814     -1.69733391591692
  H          0.71816271109305      0.80461516763107     -2.31603603596627
  C          1.54555947042134     -0.34153099890485      0.06263172493492
  C          0.96795110060382     -1.50412573263852     -0.72798451432678
  H          2.59283951838645     -0.17193485417650     -0.17637302253626
  H          1.40632590569747     -0.44465460807832      1.13567700100060
  C         -0.13689963358875     -2.31551660289080     -0.06606076712639
  H          0.66741485495623     -1.20610300875177     -1.73542649238341
  H          1.80793505139471     -2.18931302343622     -0.87801457942182
  O         -1.34157221744458     -1.54136626063791      0.05358457009433
  H         -0.32485900399335     -3.20326577076571     -0.67247813915744
  H          0.18597396645374     -2.64409647292087      0.92631869887006
  H         -2.10604981126395     -2.12460181156701     -0.01301986925071
  H         -0.75755245512553     -0.09730934230047      3.27145938864438
```

S8.4.2 135-117B_stepwise_p1c2

Source file: 135-117B_stepwise_p1c2.xyz

ORCA record: Coordinates from ORCA-job opt__TS1backward E -463.025195306902

```
24
Coordinates from ORCA-job opt_TS1backward E -463.025195306902
  O         -0.85874639719984      0.68230047675860      1.60989875760894
  C         -1.36459344490779      1.79907223133408      0.76248229507636
  H         -1.09639430355335      2.55617626807399     -1.21101956379659
  H         -1.19438570124527     -0.22200958312083      1.24639572509107
  O          1.22463898483291      0.64896730720705      0.33495351801291
  C         -0.77630115349956      1.68469975608444     -0.63398570564673
  H         -2.44892040697503      1.72948995812755      0.77948596032776
  H         -1.05215706965872      2.70102971348169      1.28485534335231
  C          0.75055240952203      1.63720935822910     -0.61989459745713
```

```
  H          -1.18620535955915      0.80483638074624     -1.13290453335499
  H           1.17024622466668      2.58985408627045     -0.29338578354057
  H           1.14934523109822      1.40791377017552     -1.60812478810236
  C           1.79318893555935     -0.57066925170395     -0.18878446482077
  C           0.80726679509809     -1.56876464497065     -0.79108085020530
  H           2.53539751142797     -0.28909696734649     -0.93796960299433
  H           2.32311343267489     -1.01306146447768      0.65546027084188
  C          -0.11842800975445     -2.31620590628194      0.15938821864994
  H           0.22286588175915     -1.11796085839468     -1.59681244212819
  H           1.42855967473041     -2.33089627075901     -1.27232097175834
  O          -1.30400610569517     -1.52784358603300      0.48551199808277
  H          -0.46561686141861     -3.22961226235745     -0.32233113089814
  H           0.39457238649227     -2.58713754365261      1.08614659799062
  H          -2.01113526220094     -2.12031238780122      0.76955235589120
  H           0.14616877485556      0.61624451469301      1.32669574023822
```

S8.4.3 135-117B_stepwise_p2c1

Source file: 135-117B_stepwise_p2c1.xyz

ORCA record: Coordinates from ORCA-job opt_initial E -463.025184294047

```
24
Coordinates from ORCA-job opt_initial E -463.025184294047
  O          -0.51647974011240      0.01491989013375      1.60242890130935
  C          -1.00216490208560      1.14868405610081      0.76567552927398
  H          -0.69388547581682      1.94067668822624     -1.18795534440332
  H          -0.84451622782628     -0.88261232346461      1.21440563491742
  O           1.59281060864187     -0.00005971613618      0.36732380992682
  C          -0.38691046979534      1.05808914484676     -0.62073579852803
  H          -2.08679002247303      1.08199675658907      0.76029042196097
  H          -0.69785622837815      2.03986242253094      1.31090819547592
  C           1.13923022833417      1.00631478591719     -0.57813368010888
  H          -0.78924875959223      0.18856510394277     -1.14344262843699
  H           1.55462744927826      1.95199910319302     -0.22677732651837
  H           1.55606292798421      0.79375311870283     -1.56265680752260
  C           2.16989619251365     -1.21038843644337     -0.16794257857372
  C           1.19383704499781     -2.19754848128880     -0.80326843787424
  H           2.92418460284641     -0.91647514839336     -0.90016220345171
  H           2.68625145845100     -1.66745161221599      0.67692604800228
  C           0.25081898828120     -2.95867189017868      0.11906711680076
  H           0.62329933388815     -1.73310081568469     -1.61122357586413
  H           1.82241907924816     -2.95257000435136     -1.28619311167964
  O          -0.94108629133336     -2.17485482480176      0.43348846177926
  H          -0.08708932368542     -3.86529066670732     -0.38165979168985
  H           0.74620593815235     -3.24226177943428      1.05157388804973
  H          -1.65309620532253     -2.77102310013364      0.69691324915015
  H           0.49339089916463     -0.04740998487264      1.34131119813647
```

S8.4.4 135-117B_stepwise_int1

Source file: 135-117B_stepwise_int1.xyz

ORCA record: Coordinates from ORCA-job opt_TS2foward_step2 E -386.538760308744

```
21
Coordinates from ORCA-job opt_TS2foward_step2 E -386.538760308744
  H           0.49128105356310     -2.24712313899819      1.70430558087065
  C          -1.54294597418128      1.84614049011768     -0.10501702842308
  H          -2.00174860315673      2.60669724084318     -0.74317307689694
  H          -1.72507195618255     -1.41568550890250      1.98533270679685
```

```
  O          0.67668947395022      0.67306662722958      0.20377250112843
  C         -0.25366932314547      1.45060442607644     -0.72692696365261
  H         -2.21251479500529      0.99028546608462     -0.04388967063980
  H         -1.39744078085694      2.26492405509019      0.88956185627507
  C          1.04465493680241      2.00792325350186     -0.34214007933183
  H         -0.29334227580919      0.96154660196543     -1.69402048170541
  H          1.10567150732286      2.73847211951396      0.45405486736891
  H          1.87619772729062      1.97510884133721     -1.03465016187084
  C          1.38558650074722     -0.55094770166893     -0.29513358637810
  C          0.43619923089723     -1.71954511364276     -0.39217633420754
  H          1.82804853055054     -0.29758351735757     -1.25896578865960
  H          2.16658813927038     -0.69209573855384      0.45039567200943
  C         -0.24766469655544     -2.12878266373323      0.90220611282184
  H         -0.31937272487537     -1.53751795486776     -1.16091744223230
  H          1.04556753694228     -2.55479848119503     -0.75252314892611
  O         -1.20185092832503     -1.12344626525195      1.23057545652490
  H         -0.73563790519153     -3.09557423045459      0.74867439197989
```

S8.4.5 135-117B_stepwise_int2

Source file: 135-117B_stepwise_int2.xyz

ORCA record: Coordinates from ORCA-job opt__TS2backward__step2 E -386.562601776783

```
21
Coordinates from ORCA-job opt_TS2backward_step2 E -386.562601776783
  H          0.45828010289914     -2.69404913503855      1.40102977412164
  C         -1.69617004190042      2.29669667590248     -0.68579061945512
  H         -2.51820299773039      2.28714570072905     -1.38940406146584
  H         -1.06915552192371     -1.24435630639667      2.41266861988569
  O          0.94248510590809      0.45549537326961      0.57595428057578
  C         -0.52602459032757      1.74113730348017     -0.97863283288234
  H          0.16051519340923      0.05930487284134      1.13158843497726
  H         -1.86968476710966      2.80037073377854      0.25862536377284
  C          0.62957366487463      1.79169333993643     -0.04886011316845
  H         -0.36978524517448      1.27263188815786     -1.94463957257174
  H          0.45298676918440      2.43091257164607      0.81272094059961
  H          1.56751182075346      2.07095325924908     -0.52486156371938
  C          1.41135817607742     -0.66303157786852     -0.29537847660827
  C          0.29100293099487     -1.66569997718671     -0.50695574057170
  H          1.75761099292725     -0.21146416145783     -1.22148155776280
  H          2.25912267778446     -1.08480712481027      0.24176513961094
  C         -0.29543455259670     -2.18223831940348      0.79759781824811
  H         -0.50140561825432     -1.22721685665844     -1.11585740840851
  H          0.70138058853869     -2.50863499130544     -1.06849396735528
  O         -0.78424482840044     -1.01550869446563      1.51913057631930
  H         -1.12694453372281     -2.86100338188896      0.60992958263299
```

S8.4.6 135-117B_stepwise_TS1

Source file: 135-117B_stepwise_TS1.xyz

ORCA record: Coordinates from ORCA-job opt__TS1__step1 E -462.951005655243

```
24
Coordinates from ORCA-job opt_TS1_step1 E -462.951005655243
  O         -1.30733051012109      0.73471742174489      2.32954712673441
  C         -1.39818836899444      2.18262633475856      0.31195180439889
  H         -1.34412858880945      2.57331736169808     -0.92232707724529
  H         -0.92698962196341     -0.06481476216501      1.92781640308123
  O          1.26770685487942      0.50851539192431      0.15438885316793
```

```
  C         -0.69314384763197      1.40437572575904     -0.58888678536653
  H         -2.44596255714225      1.99761989814029      0.50566513434669
  H         -0.89429202694891      2.95149913760875      0.88390197869626
  C          0.80359143304150      1.49115784202187     -0.74202638744890
  H         -1.22821895537152      0.61304192952172     -1.10275838276468
  H          1.17515524149079      2.47116819898939     -0.43126619012214
  H          1.11192143881234      1.30335015343298     -1.77307858273551
  C          1.88645367345511     -0.65709837009780     -0.42088703772562
  C          0.91455158963992     -1.68068072710669     -0.98810800061184
  H          2.59752144124515     -0.33972306575856     -1.18952430675723
  H          2.45291038244299     -1.08756132769837      0.40548772886971
  C         -0.02531541362280     -2.32733385415041      0.01482663411504
  H          0.33498016387157     -1.25635306226220     -1.81485531564650
  H          1.52167506694555     -2.47612579282284     -1.43159848461708
  O         -1.02182978794595     -1.37790234631130      0.45158025786602
  H         -0.52201427841787     -3.17854047253947     -0.45517594536885
  H          0.53351698402147     -2.69538766805797      0.88113039771874
  H         -1.79671145520237     -1.86591627657413      0.75429038385705
  H         -0.99585885767373      0.77604832994486      3.23990579355819
```

S8.4.7 135-117B_stepwise_TS2

Source file: 135-117B_stepwise_TS2.xyz

ORCA record: Coordinates from ORCA-job opt__TS2__step2 E -386.512786421583

```
21
Coordinates from ORCA-job opt_TS2_step2 E -386.512786421583
  H          0.03960565781562     -2.02278409596704      1.67464870735053
  C         -1.39766116691943      1.75665734759498      0.37160493147772
  H         -2.42620127063784      2.09585743058905      0.24406104788078
  H         -2.21709439617624     -1.44445180041011      1.14989405578784
  O          1.02443985847806      0.81561437848122      0.34761997006118
  C         -0.65099801829271      1.70834870155871     -0.81447613170314
  H         -1.49654013444720      0.65654040486669      0.56965207897454
  H         -0.87624466896325      2.12617613510705      1.25187281504542
  C          0.84443963349620      1.65262714274598     -0.79457968541330
  H         -1.18169398798839      1.59657811084111     -1.76027189355719
  H          1.26954820287947      2.65203473994549     -0.62779669360360
  H          1.25979161579332      1.23756227493617     -1.71473873939452
  C          1.64838546919020     -0.46024937978870      0.11585373124581
  C          0.69229817053202     -1.55563482057563     -0.33821221611876
  H          2.46372015349464     -0.34317515112928     -0.60241580966918
  H          2.08899120451580     -0.71832335412357      1.08002539390029
  C         -0.37209761915193     -1.98025353332383      0.66216116406726
  H          0.21460259640752     -1.29489719843497     -1.28965104686807
  H          1.30968155525153     -2.43369509488058     -0.54988481118642
  O         -1.49641842523755     -1.07097466510593      0.62734588250446
  H         -0.74055443003982     -2.97355757292683      0.39728724921834
```

S8.4.8 135-117B_stepwise_TS3

Source file: 135-117B_stepwise_TS3.xyz

ORCA record: Coordinates from ORCA-job opt__TS1__step3 E -386.432311883566

```
21
Coordinates from ORCA-job opt_TS1_step3 E -386.432311883566
  H          0.02773319419939     -2.32237627848729      2.01776161589863
  C         -1.23619968880866      2.83510996601560     -0.08738222857160
  H         -1.89952947941368      3.66903427069716     -0.25518874485563
```

```
  H          -1.94458721212705     -1.12874017257828      1.57090772412345
  O           0.44362447579543      0.40111915005782      0.09441639562953
  C          -0.53024490463692      2.28434012072656     -1.15253031718285
  H          -0.44569056628705     -0.03727793926517      0.47873574007929
  H          -1.09303112072251      2.50374661305145      0.93175278379865
  C           0.20564389092824      0.97054681910173     -1.21023997185773
  H          -0.75692070264762      2.64471070733644     -2.15261959619451
  H           0.55648792588905      2.59439611833663     -0.92609384288608
  H           1.07616631808957      0.89582149709658     -1.84939070629054
  C           1.45023186131664     -0.70350668272487      0.19428593502593
  C           0.75169514666311     -2.03407263214477     -0.01206110011421
  H           2.20918007037969     -0.49224779713704     -0.55353704391253
  H           1.87194736532628     -0.58725206983491      1.19103325864448
  C          -0.36104309273076     -2.26879080760610      0.99827660575212
  H           0.35452641490944     -2.09105284130323     -1.02782557338288
  H           1.49604316739611     -2.82748906511662      0.08901525745926
  O          -1.26195720712759     -1.12648410916337      0.88829297590044
  H          -0.91407585639107     -3.17953486705829      0.77239083293679
```

S8.4.9 135-117B_stepwise_117B

Source file: 135-117B_stepwise_117B.xyz

ORCA record: Coordinates from ORCA-job opt_TS1foward_step3 E -386.564267314034

```
21
Coordinates from ORCA-job opt_TS1foward_step3 E -386.564267314034
  H           0.25247680316486     -2.59214932375052      1.83251940808497
  C          -0.43146442035257      3.20083433719908     -0.06258879213389
  H          -1.32221528914920      3.82942173399671      0.00930438959403
  H          -1.94693350644376     -2.32296602387260      1.05986558759701
  O          -0.27478642922516      0.26756789675523      0.17062344500586
  C          -0.59901370905793      2.21669578152373     -1.16718719155673
  H          -0.92435969129145     -0.53312013152388      0.35426783015823
  H          -0.25818128555748      2.72958949773972      0.90254613154311
  C          -0.53379195580589      0.90102848657607     -1.10101948558508
  H          -0.80226751436701      2.60954037012262     -2.15877134277314
  H           0.40278853792878      3.87083506849790     -0.28514478496876
  H          -0.66298938218943      0.19699590143902     -1.90881275766984
  C           1.09535720383730     -0.32645700098918      0.38031957126546
  C           1.07937934145004     -1.79109841188048     -0.01077417562828
  H           1.77025427322870      0.27853513480972     -0.21778374999155
  H           1.28749440579503     -0.16804170466563      1.43996785000236
  C           0.04167288929967     -2.59261712765960      0.76061753033322
  H           0.91119248805792     -1.89555472638804     -1.08504688143397
  H           2.07049387553835     -2.20296511606265      0.19520967156640
  O          -1.24141676141818     -1.94229214037447      0.52179273401370
  H          -0.00813982489866     -3.62031819406578      0.40261586265625
```

S8.5 Reaction pathway: 135-59A

S8.5.1 135-59A_int1(RC)

Source file: 135-59A_int1(RC) .xyz

ORCA record: Coordinates from ORCA-job opt_TS1foward E -462.975941718788

```
24
Coordinates from ORCA-job opt_TS1foward E -462.975941718788
  O           0.58239500726814      2.77011045448552      0.49676095178678
```

```
  C          0.33290754209502      3.17966818015714     -0.86788776420666
  H         -0.65482102688161      2.36092247411688     -2.57643666301247
  H          1.14198940076882      3.41588665968560      0.94274243834592
  O          0.41844547455034      0.24980721735756     -0.14897575916295
  C         -0.47806544843151      2.07954847055336     -1.53490546245000
  H         -0.22075064317269      4.12082396220010     -0.87551622500400
  H          1.28444254461935      3.32855787907040     -1.38787287696991
  C          0.21579120406444      0.72601413868589     -1.51680661020101
  H         -1.45738991161448      2.00043235316917     -1.05506259359686
  H          1.19745013879675      0.77108370746257     -1.99252630580557
  H         -0.37888129341470     -0.03237281260581     -2.02475883333070
  C          0.73802431958442     -3.09348349143785      0.54357547711068
  C         -0.63431388673411     -2.75847314994810      1.02238200513227
  H          0.74098688812011     -3.28947017192535     -0.52897542874499
  H          1.46036468882686     -2.31064883371627      0.77239929744334
  C         -0.90187610944347     -1.82519691524232      2.11280746833243
  H         -1.42363490765168     -3.46010233029218      0.78052159216002
  H          1.05166243084062     -4.01170388100391      1.04687032947725
  O         -1.15940683811686     -1.36488099383742      0.72763809404511
  H         -1.81756516041052     -1.88779838787634      2.68474793631475
  H         -0.06969330508566     -1.31703067858854      2.58507034405188
  H         -0.45046233424487     -0.65621539133368      0.36000049117715
  H          0.61803385207672      1.05401808249430      0.38945402841203
```

S8.5.2 135-59A_p2c1

Source file: 135-59A_p2c1 .xyz

ORCA record: Coordinates from ORCA-job opt_initial E -463.025184294047

```
24
Coordinates from ORCA-job opt_initial E -463.025184294047
  O         -0.51647974011240      0.01491989013375      1.60242890130935
  C         -1.00216490208560      1.14868405610081      0.76567552927398
  H         -0.69388547581682      1.94067668822624     -1.18795534440332
  H         -0.84451622782628     -0.88261232346461      1.21440563491742
  O          1.59281060864187     -0.00005971613618      0.36732380992682
  C         -0.38691046979534      1.05808914484676     -0.62073579852803
  H         -2.08679002247303      1.08199675658907      0.76029042196097
  H         -0.69785622837815      2.03986242253094      1.31090819547592
  C          1.13923022833417      1.00631478591719     -0.57813368010888
  H         -0.78924875959223      0.18856510394277     -1.14344262843699
  H          1.55462744927826      1.95199910319302     -0.22677732651837
  H          1.55606292798421      0.79375311870283     -1.56265680752260
  C          2.16989619251365     -1.21038843644337     -0.16794257857372
  C          1.19383704499781     -2.19754848128880     -0.80326843787424
  H          2.92418460284641     -0.91647514839336     -0.90016220345171
  H          2.68625145845100     -1.66745161221599      0.67692604800228
  C          0.25081898828120     -2.95867189017868      0.11906711680076
  H          0.62329933388815     -1.73310081568469     -1.61122357586413
  H          1.82241907924816     -2.95257000435136     -1.28619311167964
  O         -0.94108629133336     -2.17485482480176      0.43348846177926
  H         -0.08708932368542     -3.86529066670732     -0.38165979168985
  H          0.74620593815235     -3.24226177943428      1.05157388804973
  H         -1.65309620532253     -2.77102310013364      0.69691324915015
  H          0.49339089916463     -0.04740998487264      1.34131119813647
```

S8.5.3 135-59A_p2c2

Source file: 135-59A_p2c2 .xyz

ORCA record: Coordinates from ORCA-job opt_TS1backward E -462.990235729145

```
24
Coordinates from ORCA-job opt_TS1backward E -462.990235729145
  O          1.50758071926560      2.32428863333633     -0.01986519477321
  C          0.77378860619531      2.64163383947469     -1.23516097753303
  H         -1.07687496063399      2.00913518902879     -2.10872666772005
  H          2.37287558836764      2.75018870360101      0.00115110240810
  O          0.38471086620651      0.14838955616775      0.28140586274927
  C         -0.52453200526280      1.85023135090636     -1.17941241644054
  H          0.57447865907922      3.71295693400491     -1.26615337605970
  H          1.37480303616249      2.36332468159578     -2.10483108800067
  C         -0.31882620383076      0.35565367834833     -1.01949044452377
  H         -1.15466811575427      2.21837617579504     -0.36686959997624
  H          0.31324253215628     -0.07262337700205     -1.80030439376977
  H         -1.25393045336902     -0.18688117246738     -0.92779084106635
  C          0.99957379284752     -1.17384028016481      0.57881208880758
  C         -0.00780682699323     -2.30378515569013      0.58277025659618
  H          1.78482367358340     -1.33672509788633     -0.16124659907339
  H          1.44280655457335     -1.02194379463266      1.56189572333673
  C         -1.16288808578921     -2.17436486606736      1.56545795521515
  H         -0.40141602378743     -2.48028685365748     -0.42102601928512
  H          0.57279640083272     -3.19570803162111      0.83920885864228
  O         -2.05861290961308     -1.17908819749850      1.07431561159797
  H         -1.66544699092001     -3.14290605979752      1.64172287392081
  H         -0.79080361505786     -1.90774476576064      2.56252657175523
  H         -2.86369894216456     -1.18234156224186      1.60380501516023
  H          1.04239207873877      0.93456393156544      0.36255976630052
```

S8.5.4 135-59A_int1

Source file: 135-59A_int1.xyz

ORCA record: Coordinates from ORCA-job opt__TS3foward__step2 E -193.394998130252

```
11
Coordinates from ORCA-job opt_TS3foward_step2 E -193.394998130252
  C         -1.37309777205435      0.29124450511884      0.08605448417813
  C         -0.00212680242607      0.11120270428184     -0.43814030533660
  H         -1.68910174132508      1.33169324438323      0.01495614995764
  H         -2.03716757758999     -0.30009647829022     -0.55490319539990
  H         -1.47485331291070     -0.06604997885136      1.10996276102571
  C          0.95595785741861     -0.84782187540461      0.10187835779273
  H          0.23320322690265      0.53847059319052     -1.40726590981674
  H          1.86768870494879      1.02249092878621      0.16337392870442
  O          1.12221596397583      0.56823437173359      0.60136724815493
  H          0.66975761451929     -1.48272017630826      0.93054330954196
  H          1.78949757730168     -1.18745607044888     -0.50005930914012
```

S8.5.5 135-59A_int2

Source file: 135-59A_int2.xyz

ORCA record: Coordinates from ORCA-job opt__TS1foward__step1 E -193.384971288740

```
11
Coordinates from ORCA-job opt_TS1foward_step1 E -193.384971288740
  C         -1.22237086612155      0.81739599667617     -0.07838681376529
  C         -0.43161828070360     -0.28676222475300     -0.53287338860613
  H         -0.62718299588700      1.62548756439851      0.35206692407920
  H         -1.99754051519499      1.13411319607684     -0.77322786357898
  H         -1.74911833119668      0.37742094602983      0.80510105273353
```

```
  C          0.87089374195623     -0.62001737436235     -0.02484079201626
  H         -0.81243439054283     -0.94824195641927     -1.31004182609414
  H          2.18951918060192     -0.03739132145645      1.28828159810528
  O          1.29343760236114      0.18716284014139      1.00480667486395
  H          0.90420719377469     -1.70960067103567      0.17870592774149
  H          1.48587211972862     -0.54598314250608     -0.96305777183195
```

S8.5.6 135-59A_TS1

Source file: 135-59A_TS1 .xyz

ORCA record: Coordinates from ORCA-job opt__TS1 E -462.950352782203

```
24
Coordinates from ORCA-job opt_TS1 E -462.950352782203
  O          1.05475460001042      2.62670015369200      0.29742840288130
  C          0.54307315043111      3.00137210302861     -0.99854484411831
  H         -0.88274581967793      2.23564793528770     -2.38972335945480
  H          1.72869570405202      3.25319121854192      0.58238894031283
  O          0.32924044017413      0.10895838899266     -0.05561257271227
  C         -0.52215395292389      1.98986529998785     -1.38736161669036
  H          0.11556355385490      4.00563538990815     -0.95465420634781
  H          1.35701604161890      3.00096725917816     -1.73122802937735
  C         -0.01266994922610      0.55594747975562     -1.39310894120567
  H         -1.37695599877366      2.07122881180353     -0.71056548710338
  H          0.86866112711682      0.45946325201529     -2.03497024728575
  H         -0.78022346210559     -0.12645584548315     -1.75904337599000
  C          0.95850657095548     -2.30810929231942      0.48922527664226
  C         -0.34229134563860     -2.69193941725722      0.77061624126230
  H          1.44709414297016     -2.60510531358494     -0.43217105657619
  H          1.53071988531007     -1.74776169818427      1.21723155116829
  C         -1.14028915760530     -2.06357001933335      1.89004152220837
  H         -0.89200362574117     -3.24933587609014      0.01728473141038
  H          0.61716711433188     -3.47714542819295      1.13771182605451
  O         -1.85067569107929     -1.05147403110362      1.22150063024362
  H         -1.85923346901931     -2.76123346793554      2.31487642336763
  H         -0.48733605381326     -1.68907992920437      2.68561814415239
  H         -1.20317163121028     -0.40498901915518      0.86869490671786
  H          0.79925782598848      0.86722204565266      0.35436514044016
```

S8.5.7 135-59A_TS2

Source file: 135-59A_TS2.xyz

ORCA record: Coordinates from ORCA-job opt__TS3__step2 E -193.374979799613

```
11
Coordinates from ORCA-job opt_TS3_step2 E -193.374979799613
  C         -1.35681158287730      0.36423419519317      0.07951161787290
  C         -0.19304276458170     -0.07755413743713     -0.61682568100675
  H         -1.73694999379699      1.31742053562727     -0.31639044400310
  H         -2.14049122769064     -0.33772190149145     -0.29828039786814
  H         -1.30065554635685      0.34020273310744      1.16289006136991
  C          1.00957699674247     -0.61674461945257      0.06440962472581
  H         -0.19381186276380      0.01299655106405     -1.70497172868436
  H          2.01383398697544      0.97655260267596      0.65830523258987
  O          1.32140314556701      0.39820525905184      1.00786024474092
  H          0.75425068664130     -1.54590356502994      0.59312815216842
  H          1.82269816214107     -0.83168765330864     -0.62963668190547
```

S8.5.8 135-59A_TS3

Source file: 135-59A_TS3.xyz

ORCA record: Coordinates from ORCA-job TS E -193.353280120203

```
11
Coordinates from ORCA-job TS E -193.353280120203
  C         -1.26726080270974      0.58741425641884     -0.04803784941059
  C         -0.49493983641441     -0.31054960172819     -0.71842359611669
  H         -0.06369901838355      1.03006166525985      0.44741340477445
  H         -2.05286503470669      1.13099241214726     -0.55477033390171
  H         -1.38005513675063      0.54576309457252      1.04223947137694
  C          0.78128045141829     -0.73629687445572     -0.07358034214598
  H         -0.67223644729857     -0.57202111902212     -1.75684621727702
  H          1.87482178152043      0.52654580523106      1.10426659006145
  O          0.95390839830909      0.24946106942282      0.97344901890060
  H          0.71754366886695     -1.73320845384955      0.37490956444588
  H          1.60350197614883     -0.71816225399676     -0.79061971070732
```

S8.5.9 135-59A_neutral_loss

Source file: 135-59A_neutral_loss.xyz

ORCA record: Coordinates from ORCA-job opt_neutral_loss E -269.531321228443

```
13
Coordinates from ORCA-job opt_neutral_loss E -269.531321228443
  O          2.23534989476492      3.15032249523017     -2.11522120601698
  C          1.05620915042223      3.89282906886781     -2.46329229334715
  H         -0.98097933506253      4.00213350393543     -1.86670354473643
  H          2.95103283868408      3.39134716669256     -2.71209147051886
  O          0.53770659679491      1.05474420307730     -1.22937802930254
  C         -0.10057163880841      3.40410749479194     -1.60788719387498
  H          1.22476701030979      4.96304533677220     -2.29727736582644
  H          0.82154615402956      3.74564602086397     -3.52518111557323
  C         -0.42921452660859      1.92373882614680     -1.79808581420303
  H          0.11917167043094      3.58939085127675     -0.55240525508331
  H         -0.56075679062887      1.71340616342449     -2.87092308879700
  H         -1.37517440484119      1.68965792786487     -1.30524796267618
  H          1.40728938181313      1.38833083715568     -1.48964516054386
```

S8.5.10 135-59A_59A

Source file: 135-59A_59A.xyz

ORCA record: Coordinates from ORCA-job opt_TS1backward_step1 E -193.394212184481

```
11
Coordinates from ORCA-job opt_TS1backward_step1 E -193.394212184481
  C         -1.71559242188018      0.17896988024360     -0.26166526404536
  C         -0.45802102796025      0.01987433137404     -0.68008193142776
  H          1.14347623521046      1.16017012903766      1.11922186677186
  H         -2.45873815431012      0.65865239807146     -0.88573055211129
  H         -2.05141609695789     -0.18726368997909      0.70246564386079
  C          0.52907867427230     -0.68986621423126      0.13478022773766
  H         -0.15204701040245      0.36073380192771     -1.66334021552043
  H          2.29536643865624      0.63390109277293      0.16025616119476
  O          1.59241389953880      0.36228449119585      0.77929941036626
```

```
  H          0.15024875326739     -1.15107829678258      1.04089593420507
  H          1.22156625208104     -1.33996177644591     -0.39263500321341
```

S8.6 Reaction pathway: 135-59B

S8.6.1 135-59B_int1(RC)

Source file: 135-59B_int1(RC).xyz

ORCA record: Coordinates from ORCA-job opt__TS1foward E -462.975941718788

```
24
Coordinates from ORCA-job opt_TS1foward E -462.975941718788
  O          0.58239500726814      2.77011045448552      0.49676095178678
  C          0.33290754209502      3.17966818015714     -0.86788776420666
  H         -0.65482102688161      2.36092247411688     -2.57643666301247
  H          1.14198940076882      3.41588665968560      0.94274243834592
  O          0.41844547455034      0.24980721735756     -0.14897575916295
  C         -0.47806544843151      2.07954847055336     -1.53490546245000
  H         -0.22075064317269      4.12082396220010     -0.87551622500400
  H          1.28444254461935      3.32855787907040     -1.38787287696991
  C          0.21579120406444      0.72601413868589     -1.51680661020101
  H         -1.45738991161448      2.00043235316917     -1.05506259359686
  H          1.19745013879675      0.77108370746257     -1.99252630580557
  H         -0.37888129341470     -0.03237281260581     -2.02475883333070
  C          0.73802431958442     -3.09348349143785      0.54357547711068
  C         -0.63431388673411     -2.75847314994810      1.02238200513227
  H          0.74098688812011     -3.28947017192535     -0.52897542874499
  H          1.46036468882686     -2.31064883371627      0.77239929744334
  C         -0.90187610944347     -1.82519691524232      2.11280746833243
  H         -1.42363490765168     -3.46010233029218      0.78052159216002
  H          1.05166243084062     -4.01170388100391      1.04687032947725
  O         -1.15940683811686     -1.36488099383742      0.72763809404511
  H         -1.81756516041052     -1.88779838787634      2.68474793631475
  H         -0.06969330508566     -1.31703067858854      2.58507034405188
  H         -0.45046233424487     -0.65621539133368      0.36000049117715
  H          0.61803385207672      1.05401808249430      0.38945402841203
```

S8.6.2 135-59B_p2c1

Source file: 135-59B_p2c1.xyz

ORCA record: Coordinates from ORCA-job opt__initial E -463.025184294047

```
24
Coordinates from ORCA-job opt_initial E -463.025184294047
  O         -0.51647974011240      0.01491989013375      1.60242890130935
  C         -1.00216490208560      1.14868405610081      0.76567552927398
  H         -0.69388547581682      1.94067668822624     -1.18795534440332
  H         -0.84451622782628     -0.88261232346461      1.21440563491742
  O          1.59281060864187     -0.00005971613618      0.36732380992682
  C         -0.38691046979534      1.05808914484676     -0.62073579852803
  H         -2.08679002247303      1.08199675658907      0.76029042196097
  H         -0.69785622837815      2.03986242253094      1.31090819547592
  C          1.13923022833417      1.00631478591719     -0.57813368010888
  H         -0.78924875959223      0.18856510394277     -1.14344262843699
  H          1.55462744927826      1.95199910319302     -0.22677732651837
  H          1.55606292798421      0.79375311870283     -1.56265680752260
  C          2.16989619251365     -1.21038843644337     -0.16794257857372
  C          1.19383704499781     -2.19754848128880     -0.80326843787424
```

```
  H          2.92418460284641     -0.91647514839336     -0.90016220345171
  H          2.68625145845100     -1.66745161221599      0.67692604800228
  C          0.25081898828120     -2.95867189017868      0.11906711680076
  H          0.62329933388815     -1.73310081568469     -1.61122357586413
  H          1.82241907924816     -2.95257000435136     -1.28619311167964
  O         -0.94108629133336     -2.17485482480176      0.43348846177926
  H         -0.08708932368542     -3.86529066670732     -0.38165979168985
  H          0.74620593815235     -3.24226177943428      1.05157388804973
  H         -1.65309620532253     -2.77102310013364      0.69691324915015
  H          0.49339089916463     -0.04740998487264      1.34131119813647
```

S8.6.3 135-59B_p2c2

Source file: 135-59B_p2c2.xyz

ORCA record: Coordinates from ORCA-job opt_TS1backward E -462.990235729145

```
24
Coordinates from ORCA-job opt_TS1backward E -462.990235729145
  O          1.50758071926560      2.32428863333633     -0.01986519477321
  C          0.77378860619531      2.64163383947469     -1.23516097753303
  H         -1.07687496063399      2.00913518902879     -2.10872666772005
  H          2.37287558836764      2.75018870360101      0.00115110240810
  O          0.38471086620651      0.14838955616775      0.28140586274927
  C         -0.52453200526280      1.85023135090636     -1.17941241644054
  H          0.57447865907922      3.71295693400491     -1.26615337605970
  H          1.37480303616249      2.36332468159578     -2.10483108800067
  C         -0.31882620383076      0.35565367834833     -1.01949044452377
  H         -1.15466811575427      2.21837617579504     -0.36686959997624
  H          0.31324253215628     -0.07262337700205     -1.80030439376977
  H         -1.25393045336902     -0.18688117246738     -0.92779084106635
  C          0.99957379284752     -1.17384028016481      0.57881208880758
  C         -0.00780682699323     -2.30378515569013      0.58277025659618
  H          1.78482367358340     -1.33672509788633     -0.16124659907339
  H          1.44280655457335     -1.02194379463266      1.56189572333673
  C         -1.16288808578921     -2.17436486606736      1.56545795521515
  H         -0.40141602378743     -2.48028685365748     -0.42102601928512
  H          0.57279640083272     -3.19570803162111      0.83920885864228
  O         -2.05861290961308     -1.17908819749850      1.07431561159797
  H         -1.66544699092001     -3.14290605979752      1.64172287392081
  H         -0.79080361505786     -1.90774476576064      2.56252657175523
  H         -2.86369894216456     -1.18234156224186      1.60380501516023
  H          1.04239207873877      0.93456393156544      0.36255976630052
```

S8.6.4 135-59B_int1

Source file: 135-59B_int1.xyz

ORCA record: Coordinates from ORCA-job opt_TS1foward_step1 E -193.386985009085

```
11
Coordinates from ORCA-job opt_TS1foward_step1 E -193.386985009085
  C         -1.28123791916727      0.18470096104749      0.32767984197816
  C         -0.10604508293830      0.00464802581026     -0.55837654264524
  H         -1.76468054514751      1.14664074486624      0.15456524656366
  H         -2.00397464588833     -0.59336971037440      0.05795887821488
  H         -1.03945432445249      0.06203534438005      1.38465554886510
  C          1.11598473509596     -0.68698476079726     -0.16137268173622
  H         -0.23995964272569      0.18103415809935     -1.61934230310463
  H          1.13023000526075      1.19406040240755      0.72720990827022
  O          1.19787971950513      0.82400069804356     -0.17472317973278
```

```
  H          1.19369632370181     -1.10412497245941      0.83561394584262
  H          1.79003136863681     -1.08687894924893     -0.90709468466069
```

S8.6.5 135-59B_TS1

Source file: 135-59B_TS1.xyz

ORCA record: Coordinates from ORCA-job opt_TS1 E -462.950352782203

```
24
Coordinates from ORCA-job opt_TS1 E -462.950352782203
  O          1.05475460001042      2.62670015369200      0.29742840288130
  C          0.54307315043111      3.00137210302861     -0.99854484411831
  H         -0.88274581967793      2.23564793528770     -2.38972335945480
  H          1.72869570405202      3.25319121854192      0.58238894031283
  O          0.32924044017413      0.10895838899266     -0.05561257271227
  C         -0.52215395292389      1.98986529998785     -1.38736161669036
  H          0.11556355385490      4.00563538990815     -0.95465420634781
  H          1.35701604161890      3.00096725917816     -1.73122802937735
  C         -0.01266994922610      0.55594747975562     -1.39310894120567
  H         -1.37695599877366      2.07122881180353     -0.71056548710338
  H          0.86866112711682      0.45946325201529     -2.03497024728575
  H         -0.78022346210559     -0.12645584548315     -1.75904337599000
  C          0.95850657095548     -2.30810929231942      0.48922527664226
  C         -0.34229134563860     -2.69193941725722      0.77061624126230
  H          1.44709414297016     -2.60510531358494     -0.43217105657619
  H          1.53071988531007     -1.74776169818427      1.21723155116829
  C         -1.14028915760530     -2.06357001933335      1.89004152220837
  H         -0.89200362574117     -3.24933587609014      0.01728473141038
  H          0.61716711433188     -3.47714542819295      1.13771182605451
  O         -1.85067569107929     -1.05147403110362      1.22150063024362
  H         -1.85923346901931     -2.76123346793554      2.31487642336763
  H         -0.48733605381326     -1.68907992920437      2.68561814415239
  H         -1.20317163121028     -0.40498901915518      0.86869490671786
  H          0.79925782598848      0.86722204565266      0.35436514044016
```

S8.6.6 135-59B_TS2

Source file: 135-59B_TS2.xyz

ORCA record: Coordinates from ORCA-job opt_TS E -193.337004021695

```
11
Coordinates from ORCA-job opt_TS E -193.337004021695
  C         -1.19363876378192      0.20777875689434      0.37289903869946
  C          0.10311092682930      0.13259750478559     -0.43706534205554
  H         -1.76348985170733      1.07794795845481      0.05352115468151
  H         -1.78953010862972     -0.68848467691632      0.19357264631928
  H         -0.98389727365390      0.29258306063431      1.44041227067435
  C          0.98725509947232     -0.99081911397915     -0.21135262159935
  H         -0.17047287805091      0.06232646183638     -1.51515015787175
  H          1.11570821669529      1.44924246983868      0.63535442844443
  O          0.97374867153330      1.24942331121459     -0.30344405132058
  H          0.73161233782297     -1.85547436679774      0.40340455451822
  H          1.98959362347060     -0.93712136596550     -0.63215192049004
```

S8.6.7 135-59B_neutral_loss

Source file: 135-59B_neutral_loss.xyz

ORCA record: Coordinates from ORCA-job opt__neutral__loss E -269.531321228443

```
13
Coordinates from ORCA-job opt_neutral_loss E -269.531321228443
  O          2.23534989476492      3.15032249523017     -2.11522120601698
  C          1.05620915042223      3.89282906886781     -2.46329229334715
  H         -0.98097933506253      4.00213350393543     -1.86670354473643
  H          2.95103283868408      3.39134716669256     -2.71209147051886
  O          0.53770659679491      1.05474420307730     -1.22937802930254
  C         -0.10057163880841      3.40410749479194     -1.60788719387498
  H          1.22476701030979      4.96304533677220     -2.29727736582644
  H          0.82154615402956      3.74564602086397     -3.52518111557323
  C         -0.42921452660859      1.92373882614680     -1.79808581420303
  H          0.11917167043094      3.58939085127675     -0.55240525508331
  H         -0.56075679062887      1.71340616342449     -2.87092308879700
  H         -1.37517440484119      1.68965792786487     -1.30524796267618
  H          1.40728938181313      1.38833083715568     -1.48964516054386
```

S8.6.8 135-59B_59B

Source file: 135-59B_59B.xyz

ORCA record: Coordinates from ORCA-job opt__backwards E -193.443855492979

```
11
Coordinates from ORCA-job opt_backwards E -193.443855492979
  C         -1.29889812053070      0.26273458064369      0.44585157408497
  C          0.10509217697648      0.32736947775882      0.02777683086596
  H         -1.76069970189198      1.24680853746860      0.48012182571060
  H         -1.85885258982009     -0.43032780658007     -0.18503288147364
  H         -1.31155152281838     -0.16956706425424      1.45779605398300
  C          0.85104576113466     -0.85088824263609     -0.44102228975243
  H          0.53430237939351     -1.04167818824688     -1.47679453137800
  H          1.59498821538988      1.49712745339438     -0.16562960797935
  O          0.65105507285939      1.47942208936336      0.08754312169916
  H          0.56926618380924     -1.73745153276426      0.12994556307284
  H          1.93178215340284     -0.70931124626535     -0.42732963609739
```

S8.7 Reaction pathway: 135-77

S8.7.1 135-77_77(RC)

Source file: 135-77_77(RC).xyz

ORCA record: Coordinates from ORCA-job opt__TS1backward E -462.977929806134

```
24
Coordinates from ORCA-job opt_TS1backward E -462.977929806134
  O         -1.78623399055982      0.97877527840577      1.03119899829651
  C         -1.52536036603004      2.39158154028908      0.81512007670550
  H         -0.76621486038525      3.57494056191189     -0.80258804093745
  H         -1.99735205517742      0.79241283853508      1.95362777112521
  O         -0.19022532304052      0.25871108588895     -0.69490165135585
  C         -1.02783204641011      2.53066096682368     -0.61655744789348
  H         -2.44624616779397      2.95663153619375      0.96135666772311
  H         -0.77519527484531      2.73844379290108      1.53085070904150
  C          0.19793223484715      1.68579422085448     -0.90343588473172
  H         -1.82448190663233      2.27033038907226     -1.31717493518735
  H          1.02692527414333      1.90122448803554     -0.22901251503538
```

```
  H          0.52429784713158      1.75291568649566     -1.93759746099250
  C          2.29447008988620     -1.36538600776097     -0.03396611908041
  C          1.58329438974711     -2.35453976672811     -0.58020782617946
  H          3.16982754362311     -0.96858216319745     -0.53212524000762
  H          2.07041509854965     -0.99492211701308      0.96007715663240
  C          0.38863969680139     -3.00413839275618      0.04542092462916
  H          0.58489442989271     -0.36714001493954     -0.59168256016147
  H          1.87562706676331     -2.75898196405208     -1.54479912907345
  O         -0.14983154737492     -2.14966111786045      1.05467067908410
  H         -0.36316809306199     -3.21433504592430     -0.72464486058122
  H          0.69499857546565     -3.96945702861875      0.46697047270479
  H         -0.70943880433747     -2.66978027502705      1.64130524437452
  H         -0.81879988625280      0.23498543466246      0.12745624264579
```

S8.7.2 135-77_TS1

Source file: 135-77_TS1.xyz

ORCA record: Coordinates from ORCA-job opt__TS1 E -462.931259022403

```
24
Coordinates from ORCA-job opt_TS1 E -462.931259022403
  O         -1.71729289584695      1.46869750419695      1.31239037532058
  C         -1.63055516050697      2.73721329257502      0.62943885101712
  H         -0.90263156285111      3.44753092627440     -1.24798494807463
  H         -2.09104138956245      1.59017631964738      2.19204717389382
  O          0.25782524964292      0.46769755825826     -0.15012216907278
  C         -1.01181546822147      2.48727470523465     -0.73661572428228
  H         -2.62732486245065      3.17132756674770      0.52113958236727
  H         -1.01128029560042      3.42844491639133      1.21085073720314
  C          0.35619351971796      1.82032548942036     -0.66670343545981
  H         -1.68406513804174      1.87418651714233     -1.34247483012033
  H          1.03552075286347      2.39365942753183     -0.02790765888647
  H          0.80154803540497      1.74006852572918     -1.65831205520457
  C          1.90339534045203     -1.30613462834290      0.06802556024985
  C          1.25354681731730     -2.24632068727588     -0.73318886777824
  H          2.59853857165877     -0.59279340873794     -0.35484778771063
  H          1.69467557126227     -1.25414360311744      1.12899009093562
  C          0.48052678359491     -3.39823351111510     -0.11883095558035
  H          0.54303167543721     -1.30233280971566     -0.82234170166465
  H          1.63539288685827     -2.39805517751191     -1.74022680553406
  O          0.18749580399535     -3.04967164027805      1.21569651685300
  H         -0.42233419844709     -3.58763583144342     -0.70772882894226
  H          1.11420373205494     -4.29175826876164     -0.18134163707000
  H         -0.37590451617309     -3.71947812193495      1.61963959082643
  H         -0.38764925255840      0.51995493908550      0.59040892671421
```

S8.7.3 135-77_neutral_loss

Source file: 135-77_neutral_loss.xyz

ORCA record: Coordinates from ORCA-job opt__neutral__loss E -193.082680792235

```
10
Coordinates from ORCA-job opt_neutral_loss E -193.082680792235
  H          3.36450408477196     -5.01279537544220     -0.92899016970502
  H          3.68131234331857     -4.08824076032428      0.55286889385435
  C          5.39082601471012     -2.60407035867255     -0.77651946357974
  C          4.26422477758097     -3.11356168942338     -1.25614295459921
  H          6.03830228751088     -1.98472381972555     -1.38438075106033
  H          5.70967176533892     -2.78970481480772      0.24349770001327
```

```
  C          3.34463839979673     -4.00622325895023     -0.48779771868689
  H          1.40377197199925     -4.12327696028700     -0.22415694256741
  H          3.95712568100658     -2.90228922633700     -2.27648679213911
  O          2.02257558616600     -3.46676142773007     -0.56083636652990
```

S8.7.4 135-77_77

Source file: 135-77_77.xyz

ORCA record: Coordinates from ORCA-job opt_77mz E -269.867863166411

```
14
Coordinates from ORCA-job opt_77mz E -269.867863166411
  O         -0.43133420568196     -0.49170133496133      1.40635988692338
  C         -0.47721190587157      0.67973616694709      0.51397653176239
  H          0.39906993110427      1.17578774442137     -1.37422235467494
  H         -0.76960677846553     -0.30055642835047      2.29256460246453
  O          1.73723389010948     -1.16288789505160      0.69607862819749
  C          0.35573173293326      0.31256223775337     -0.70532237355328
  H         -1.51697603667956      0.86556590837275      0.25249406947521
  H         -0.06896690941525      1.54117889290501      1.04518210026614
  C          1.77880397287707     -0.07811231590926     -0.34325538551624
  H         -0.13133013842608     -0.49392078939234     -1.25898283799576
  H          2.33494356530313      0.72277062720705      0.13900374213591
  H          2.34845630869401     -0.46220851243938     -1.18512375480160
  H          0.74684059874519     -1.01068310897683      1.21086604044136
  H          1.84320291427353     -2.05076563942541      0.31960396947541
```

S9 ForMileS output used to generate molecular graphic structures

The ForMileS outputs below are reported as supplied. These run summaries document the formula, charge state, target mass tolerance, timing/memory snapshots, number of generated nSMILES entries, number of filtered charged SMILES entries, and output filenames used in the molecular-structure generation workflow.

Table S14: Summary of ForMileS molecular-graph generation records used for the selected product-ion formulas.

ForMileS record	Formula	Target mass	nSMILES count	Filtered charged SMILES count
run_summary_ion59mz(1)	C3O	59.05+/-0.5	36	17
run_summary_ion77mz(1)	C3O2	77.06+/-0.5	138	18
run_summary_ion117mz(1)	C6O2	117.092+/-0.5	10	18

S9.1 ForMileS run summary: C3O (59.05 +/- 0.5)

Source file: run_summary_ion59mz(1).txt

```
ForMileS Run Summary
====================

Formula: C3O
Charge: 1
Target mass: 59.05 +/- 0.5

Timings & Memory
-----------------
Molecular graph generation:   time = 0.021 s | RAM ~ 70.2 MB
Charge generation:            time = 0.053 s | RAM ~ 71.0 MB
Total wall-time:              time = 0.367 s | RAM ~ 74.2 MB

Counts
------
nSMILES file count:                 36
filteredchargedSMILES file count:   17

Files
-----
nSMILES file:               nSMILES_C3O.txt
charged file:               chargedSMILES_C3O.txt
filtered charged file:      filteredchargedSMILES_C3O.txt

Notes
-----
RAM values are instantaneous RSS snapshots taken right after each step.
```

S9.2 ForMileS run summary: C3O2 (77.06 +/- 0.5)

Source file: `run_summary_ion77mz(1).txt`

```
ForMileS Run Summary
====================

Formula: C3O2
Charge: 1
Target mass: 77.06 +/- 0.5

Timings & Memory
-----------------
Molecular graph generation:   time = 0.126 s | RAM ~ 71.4 MB
Charge generation:            time = 0.227 s | RAM ~ 72.2 MB
Total wall-time:              time = 0.618 s | RAM ~ 74.9 MB

Counts
------
nSMILES file count:                 138
filteredchargedSMILES file count:   18

Files
-----
nSMILES file:               nSMILES_C3O2.txt
charged file:               chargedSMILES_C3O2.txt
filtered charged file:      filteredchargedSMILES_C3O2.txt

Notes
-----
RAM values are instantaneous RSS snapshots taken right after each step.
```

S9.3 ForMileS run summary: C6O2 (117.092 +/- 0.5)

Source file: `run_summary_ion117mz(1).txt`

```
ForMileS Run Summary
====================

Formula: C6O2
Charge: 1
Target mass: 117.092 +/- 0.5

Timings & Memory
-----------------
Molecular graph generation:   time = 0.050 s | RAM ~ 62.9 MB
Charge generation:            time = 0.041 s | RAM ~ 63.4 MB
Total wall-time:              time = 0.551 s | RAM ~ 66.1 MB

Counts
------
nSMILES file count:                 10
filteredchargedSMILES file count:   18

Files
-----
nSMILES file:               nSMILES_C6O2.txt
charged file:               chargedSMILES_C6O2.txt
filtered charged file:      filteredchargedSMILES_C6O2.txt

Notes
-----
RAM values are instantaneous RSS snapshots taken right after each step.
```
